# PTEN counteracts FBXL2 to promote IP3R3– and Ca^2+^–mediated apoptosis limiting tumour growth

**DOI:** 10.1038/nature22965

**Published:** 2017-06-14

**Authors:** Shafi Kuchay, Carlotta Giorgi, Daniele Simoneschi, julia Pagan, Sonia Missiroli, Anita Saraf, Laurence Florens, Michael P. Washburn, Ana Collazo-Lorduy, Mireia Castillo-Martin, Carlos Cordon-Cardo, Said M. Sebti, Paolo Pinton, Michele Pagano

**Affiliations:** 1 Department of Biochemistry and Molecular Pharmacology, New York University School of Medicine, 522 First Avenue, SRB 1107, New York, New York 10016, USA; 2 NYU Perlmutter Cancer Center, New York University School of Medicine, 522 First Avenue, SRB 1107, New York, New York 10016, USA; 3 Howard Hughes Medical Institute, New York University School of Medicine, 522 First Avenue, SRB 1107, New York, New York 10016, USA; 4 Department of Morphology, Surgery and Experimental Medicine, Section of Pathology, Oncology and Experimental Biology, Laboratory for Technologies of Advanced Therapies (LTTA), University of Ferrara, Ferrara, Italy; 5 The Stowers Institute for Medical Research, 1000 East 50th Street, Kansas City, Missouri 64110, USA; 6 Department of Pathology and Laboratory Medicine, The University of Kansas Medical Center, 3901 Rainbow Boulevard, Kansas City, Kansas 66160, USA; 7 Department of Pathology at Icahn School of Medicine at Mount Sinai, New York, New York 10029 USA; 8 Spanish Society of Medical Oncology, Madrid, Spain; 9 Department of Pathology at Champalimaud Centre for the Unknown, Lisbon, Portugal; 10 Drug Discovery Department, Moffitt Cancer Center, and Department of Oncologic Sciences, University of South Florida, Tampa, Florida 33612, USA

## Abstract

In response to environmental cues that promote IP3 (inositol 1,4,5-trisphosphate) generation, IP3 receptors (IP3Rs) located on the endoplasmic reticulum allow the ‘quasisynaptical’ feeding of calcium to the mitochondria to promote oxidative phosphorylation^1^. However, persistent Ca^2+^ release results in mitochondrial Ca^2+^ overload and consequent apoptosis^2^. Among the three mammalian IP3Rs, IP3R3 appears to be the major player in Ca^2+^-dependent apoptosis. Here we show that the F-box protein FBXL2 (the receptor subunit of one of 69 human SCF (SKP1, CUL1, F-box protein) ubiquitin ligase complexes^3^) binds IP3R3 and targets it for ubiquitin-, p97- and proteasome-mediated degradation to limit Ca^2+^ influx into mitochondria. FBXL2-knockdown cells and FBXL2-insensitive IP3R3 mutant knock-in clones display increased cytosolic Ca^2+^ release from the endoplasmic reticulum and sensitization to Ca^2+^-dependent apoptotic stimuli. The phosphatase and tensin homologue (*PTEN*) gene is frequently mutated or lost in human tumours and syndromes that predispose individuals to cancer^4^. We found that PTEN competes with FBXL2 for IP3R3 binding, and the FBXL2-dependent degradation of IP3R3 is accelerated in *Pten*^−/−^ mouse embryonic fibroblasts and *PTEN*-null cancer cells. Reconstitution of *PTEN*-null cells with either wild-type PTEN or a catalytically dead mutant stabilizes IP3R3 and induces persistent Ca^2+^ mobilization and apoptosis. IP3R3 and PTEN protein levels directly correlate in human prostate cancer. Both in cell culture and xenograft models, a non-degradable IP3R3 mutant sensitizes tumour cells with low or no PTEN expression to photodynamic therapy, which is based on the ability of photosensitizer drugs to cause Ca^2+^-dependent cytotoxicity after irradiation with visible light^5,6^. Similarly, disruption of FBXL2 localization with GGTi-2418, a geranylgeranyl transferase inhibitor^7^, sensitizes xenotransplanted tumours to photodynamic therapy. In summary, we identify a novel molecular mechanism that limits mitochondrial Ca^2+^ overload to prevent cell death. Notably, we provide proof-of-principle that inhibiting IP3R3 degradation in PTEN-deregulated cancers represents a valid therapeutic strategy.

To identify FBXL2 substrates, FBXL2 was expressed in HEK293T cells, immunopurified and analysed for co-purifying proteins by mass spectrometry, which revealed the presence of two unique peptides corresponding to IP3R3. To confirm these results, we screened a panel of human F-box proteins and found that the only F-box protein that co-immunoprecipitated IP3R3 was FBXL2 (Extended Data Fig. 1a). FBXL2 co-immunoprecipitated SKP1, CUL1 and IP3R3 from the membrane fraction (Extended Data Fig. 1b). FBXL2 contains a C-terminal CaaX domain that is required for its geranylgeranylation and localization at cell membranes^8^. In contrast to wild-type FBXL2, FBXL2(CaaX/SaaX), a geranylgeranylation-deficient mutant in which a cysteine in the CaaX domain has been mutated to serine^9^, did not fractionate with cellular membranes and did not interact with IP3R3 and neddylated CUL1 (Extended Data Fig. 1c, d).

We also observed that expression of wild-type FBXL2, but not FBXL2(ΔF-box), an inactive mutant, induced a decrease in the levels of IP3R3 (this decrease was rescued by MG132 treatment) and FBXL2(ΔF-box) bound more IP3R3 than wild-type FBXL2 (this difference was abolished by MG132 treatment) (Extended Data Fig. 1d, e).

Since IP3-mediated Ca^2+^ release is stimulated by mitogens, we examined the impact of serum on IP3R3 levels. The growth of normal human fibroblasts (NHFs) was arrested by serum deprivation after which serum was reintroduced. The levels of IP3R3 decreased in control cells, but much less in cells treated with MG132 or lactacystin, or in which *FBXL2* was silenced (Extended Data Fig. 1f–i). In hTERT RPE-1 cells, elimination of one *FBXL2* allele resulted in IP3R3 stabilization (Extended Data Fig. 1j–l). GGTi-2418 treatment delocalized FBXL2 and stabilized IP3R3 (Extended Data Fig. 2a–c). Eer1, an inhibitor of p97 (also known as VCP or Cdc48), a segregase that extracts ubiquitinated proteins from the cellular membranes to facilitate their proteasomal degradation^10^, blocked IP3R3 degradation (Extended Data Fig. 2d). Silencing of p97 inhibited the serum-mediated degradation of IP3R3, and both FBXL2 and IP3R3 co-immunoprecipitated with p97 (Extended Data Fig. 2e, f). Finally, immunopurified FBXL2, but not FBXL2(ΔF-box), promoted the *in vitro* ubiquitination of IP3R3 (Extended Data Fig. 2g, h).

To investigate the role of FBXL2 in Ca^2+^ homeostasis, we measured the changes in Ca^2+^ concentration in both the cytosol and mitochondria of NHFs in response to ATP, a purinergic GPCR agonist that induces IP3 production and rapid flow of Ca^2+^ from the endoplasmic reticulum to the mitochondria^11^. Serum starvation caused an increase and serum re-addition induced a decrease in Ca^2+^ mobilization (Fig. 1a and Extended Data Fig. 3a). *FBXL2* silencing or treatment with MG132 or GGTi-2418 inhibited the serum-mediated decrease in Ca^2+^ mobilization (Fig. 1a and Extended Data Fig. 3b, c). Conversely, cells engineered to express FBXL2, but not FBXL2(CaaX/SaaX), displayed low IP3R3 levels and a decrease in Ca^2+^ mobilization (Extended Data Figs 1c and 3d, e).

Serum starvation sensitized NHFs to treatment with H_2_O_2_, an oxidizing agent that induces persistent release of Ca^2+^ from the endoplasmic reticulum and consequent apoptosis, but serum re-addition alleviated this sensitivity (Fig. 1b). Compared to cells re-stimulated with serum, serum-starved cells displayed an increase in cleaved PARP, cleaved caspase-3, and cytochrome *c* release (Fig. 1c, d), all signatures of apoptosis. In cells re-stimulated with serum, FBXL2 knockdown caused IP3R3 accumulation (Extended Data Fig. 1h, i), sensitization to H_2_O_2_, and an increase in the apoptotic signature and in mitochondrial Ca^2+^ uptake (Fig. 1b–d and Extended Data Fig. 3f). Conversely, expression of wild-type FBXL2, but not FBXL2(CaaX/SaaX), induced resistance to H_2_O_2_, but not to etoposide (Extended Data Fig. 3g). Inhibition of mitochondrial Ca^2+^ overload by silencing *MCUA* (mitochondrial calcium uniporter, isoform a) or preventing the PTP opening using cyclosporin-A abolished the sensitization to H_2_O_2_ by *FBXL2* silencing (Fig. 1b).

Next, we mapped the FBXL2 binding domain (that is, the degron) in IP3R3 and narrowed it to a region located between amino acids 436– 587 (Extended Data Fig. 4a, b). Fragments encoding IP3R3(436–587) and IP3R3(227–602) interacted with FBXL2 more stably than IP3R3 (1–602), suggesting that the N-terminal suppressor domain of IP3R3 inhibits the FBXL2–IP3R3 interaction. Treatment of cells with ATP, which induces IP3 production and repositioning of the N-terminal suppressor domain^11^, increased the binding between FBXL2 and IP3R3, particularly upon proteasome inhibition (Extended Data Fig. 4c, d). This suggests that once IP3 unmasks the IP3R3 degron, FBXL2 binds IP3R3 and this interaction is maintained, particularly if the degradation of IP3R3 is inhibited. Finally, ATP promoted the degradation of IP3R3 in cells cultured in the presence, but not in the absence, of serum (Extended Data Fig. 4e).

We then fine-mapped the degron to a region between amino acids 545 and 566 and found that Phe553 (which is highly conserved in IP3R3 orthologues, but not in its paralogues), and to a lesser extent Gln550 and Arg554, were necessary for efficient binding of IP3R3 to FBXL2 (Extended Data Fig. 4f–i). IP3R3(Q-FR/A-AA), a mutant in which Gln550, Phe553 and Arg554 were mutated to Ala, displayed a longer half-life than wild-type IP3R3, and it was not degraded when serum-starved cells were re-stimulated with serum (Fig. 1e, f). Expression of FBXL2 resulted in increased ubiquitination of IP3R3, but IP3R3(Q-FR/A-AA) remained poorly ubiquitinated (Extended Data Fig. 4j).

Importantly, expression of IP3R3(Q-FR/A-AA) recapitulated the phenotypes observed upon *FBXL2* silencing: enhanced Ca^2+^ release from the endoplasmic reticulum following serum treatment and agonist stimulation, and sensitization to H_2_O_2_ (Fig. 1g–i and Extended Data Fig. 3h, i). Moreover, in cells expressing IP3R3(Q-FR/A-AA), MG132 did not produce an increase of the Ca^2+^ response (Fig. 1g and Extended Data Fig. 3h).

Certain tumour suppressors and oncoproteins with roles in Ca^2+^ homeostasis localize to the endoplasmic reticulum (for example, AKT1, BCL2, p53, PML4, PTEN and KRAS4B)^12^. When these six proteins were expressed in HEK293T cells, PTEN was the only one able to robustly interact with endogenous IP3R3, and the interaction between PTEN and IP3R3 was confirmed at the endogenous level (Extended Data Fig. 5a–c). Compared to *Pten*^+/+^ mouse embryonic fibroblasts (MEFs)^13^, the steady-state levels of IP3R3 were lower and its half-life was shorter in *Pten*^−/−^ MEFs (Extended Data Fig. 5d, e). Importantly, in response to mitogens, IP3R3 was degraded faster in *Pten*^−/−^ than in *Pten*^+/+^ MEFs, and this phenotype was reverted by silencing *FBXL2* (Fig. 2a). Finally, after serum re-addition, IP3R3 was ubiquitinated to a greater extent in *Pten*^−/−^ than in *Pten*^+/+^ MEFs (Extended Data Fig. 5f).

PTEN reconstitution in *Pten*^−/−^ MEFs and cell lines expressing no or low levels of PTEN induced an increase in the levels of IP3R3 (Fig. 2b and Extended Data Fig. 5g). PTEN(C124S), a catalytically dead mutant^4^, was also able to enhance IP3R3 levels and induce apoptotic cleavage of caspase-3, although not as efficiently as wild-type PTEN, (Fig. 2b and Extended Data Fig. 5g). Silencing of IP3R3 counteracted the apoptotic cleavage of caspase-3 induced by PTEN(C124S) (Fig. 2b). Expression of wild-type PTEN or, to a lesser extent, PTEN(C124S) in *Pten*^−/−^ MEFs augmented Ca^2+^ mobilization from the endoplasmic reticulum in an IP3R3-dependent manner (Fig. 2c and Extended Data Fig. 5h). PTEN(G129E), a mutant displaying a greatly reduced lipid phosphatase activity, but retaining protein phosphatase activity^4^, induced an increase in the Ca^2+^ response identical to that induced by wild-type PTEN (Fig. 2c and Extended Data Fig. 5h). Finally, PTEN, PTEN(C124S) and PTEN(G129E) bound comparable amounts of IP3R3 (Extended Data Fig. 5i).

Like FBXL2, PTEN interacted more stably with IP3R3(436–587) and accordingly IP3R3(Q-FR/A-AA), which is impaired in its binding to FBXL2, interacted less with PTEN (Extended Data Fig. 6a, b), suggesting that PTEN and FBXL2 compete for the same region in IP3R3. Indeed, increasing amounts of FBXL2 interfered with the binding between PTEN and IP3R3, and increasing amounts of PTEN interfered with the binding between FBXL2 and IP3R3 (Extended Data Fig. 6c–e). Conversely, PTEN silencing, in addition to reducing IP3R3 levels, resulted in an increased interaction between IP3R3 and FBXL2 (Extended Data Fig. 6f).

Compared to cells expressing PTEN, IP3R3 levels were significantly lower in cell lines expressing no or low levels of PTEN, although all cell lines expressed similar levels of FBXL2 mRNA (Extended Data Fig. 6g, h). The half-life of IP3R3 was significantly shorter in A549 cells compared to H460 (the former expressing low PTEN levels), and IP3R3 levels increased in U87 and A549 cells after reconstitution of PTEN expression or upon FBXL2 inhibition by GGTi-2418 (Extended Data Figs 5g and 6i, j). We also assessed the expression of PTEN and IP3R3 in tissue microarrays generated from human prostate adenocarcinomas and found that there was a significant, direct correlation between IP3R3 and PTEN expression levels (Fig. 2d and Extended Data Fig. 6k).

PDT-induced Ca^2+^ mobilization was significantly reduced when PTEN or IP3R3 were depleted, and, compared to cell lines expressing PTEN and high levels of IP3R3, cancer cell lines expressing no or low levels of PTEN and low levels of IP3R3 displayed less Ca^2+^ mobilization and less apoptotic markers upon photodynamic therapy (PDT) treatment (Extended Data Fig. 7a, b). Re-expression of either wild-type PTEN or PTEN(C124S) increased PDT-induced Ca^2+^ mobilization and apoptotic cleavage of caspase-3 and PARP (Extended Data Fig. 7c, d). Depletion of FBXL2 resulted in increased PDT-induced Ca^2+^ mobilization and apoptosis in cells with no or low levels of PTEN, which depended on the presence of IP3R3 (Fig. 3a and Extended Data Fig. 8a). Similarly, expression of IP3R3(Q-FR/A-AA) increased PDT-induced Ca^2+^ mobilization and apoptosis (Extended Data Fig. 8b). We also generated A549 and PC3 knock-in clones expressing IP3R3(Q-FR/A-AA) (Extended Data Fig. 9a–c). Compared to parental cells, these clones displayed the stabilization of IP3R3, released more Ca^2+^ from the endoplasmic reticulum, and were more prone to apoptosis when treated with PDT (Fig. 3b and Extended Data Fig. 9d–g).

We then stably transfected A549 and PC3 cells with either an empty vector or IP3R3(Q-FR/A-AA) for xenograft transplantation experiments in NOD/SCID gamma mice. These xenografts did not show any significant difference in growth curves (Fig. 4a and Extended Data Fig. 10a). However, PDT significantly reduced the tumour weight and growth rate of IP3R3(Q-FR/A-AA) expressing xenografts, whereas empty vector xenografts were unaffected (Fig. 4a and Extended Data Fig. 10a). Accordingly, in response to PDT, increased apoptosis was detected in IP3R3(Q-FR/A-AA) xenografts compared to empty vector xenografts (Fig. 4b and Extended Data Fig. 10b). Virtually identical results were obtained using a A549 knock-in clone expressing endogenous IP3R3(Q-FR/A-AA) (Extended Data Fig. 10c, d).

We also administered intratumoural injections of GGTi-2418, which has reached phase I clinical trial^7^, delocalizes FBXL2 from membranes, and stabilizes IP3R3 (see above). GGTi-2418, by itself, only modestly affected the growth rate of xenografts. However, GGTi-2418 significantly sensitized tumour xenografts to PDT (Fig. 4c, d).

Recent studies suggest that non-catalytic activities of PTEN contribute to its tumour suppressor function through poorly defined mechanisms. Our study reveals a phosphatase-independent mechanism by which PTEN functions as a tumour suppressor (Extended Data Fig. 10e). Moreover, we show that when IP3R3 degradation is inhibited, tumours with no or low levels of PTEN expression become sensitive to PDT. We propose that such tumours should not only be treated with inhibitors targeting the PI3K signalling cascade (which is hyperactive in these cancers), but also with drugs that result in IP3R3 stabilization, thereby abrogating both arms of PTEN function.

## Methods

No statistical methods were used to predetermine sample size and the investigators were not blinded to allocation during experiments and outcome assessment.

### Antibodies, reagents, and biochemical methods

Immunoprecipitation and immunoblotting experiments were performed as previously described^9,14,15^. In some experiments, 2–4% of whole-cell lysate inputs (depending on the protein of interest) were run together with immunoprecipitates. The following antibodies were used: IP3R1(Bethyl no. A302-157A), IP3R-2(Millipore no. AB3000), IP3R3 (BD-Pharmingen no. 610312), PTEN (Cell Signaling Technology no. 9559S), calnexin (Santa Cruz no. sc11397), cleaved PARP (Cell Signaling Technology no. 5625S), cleaved caspase-3 (Cell Signaling Technology #9661S), cytochrome *c* (BD no. 556433), AKT (Cell Signaling Technology no. 2920S), pAKT-S473 (Cell Signaling Technology no. 4058S), p97 (Thermo Scientific no. PA5-22257), p85|3 (AbCam no. ab28356), GFP (Cell Signaling Technology no. 2956S), CUL1 (Invitrogen no. 718700), SKP1 (generated in-house), Flag (Sigma), HA (Covance), α-tubulin (Sigma), and β-actin (Sigma). Isotype-specific horseradish peroxidase conjugated secondary antibodies were used for detection by enhanced chemiluminescence (Pierce). All cDNA constructs were N-terminally tagged either with Flag, HA, GFP or GST (specific details provided on request).

### Cell culture and transfections

Cell lines and primary fibroblasts were purchased from ATCC, except where indicated. All cell lines were treated with Plasmocin and tested with Universal Mycoplasma Detection Kit from ATCC. NHFs were grown in Dulbecco's modified Eagle's medium (DMEM) supplemented with 10% FBS. MEFs (*Pten*^+/+^ and *Pten*^−/−^, provided by R. Parsons' laboratory)^13^, HeLa, and COS-7 cells (African Green Monkey SV40-transformed kidney fibroblasts) were grown in DMEM supplemented with 10% FBS and transfected as described^9^. All cells grown in culture were periodically monitored for mycoplasma contamination. Cells were starved for the indicated time periods in 0.1% FBS. Cells were serum-stimulated with DMEM supplemented with 10% FBS. Where indicated, cells were treated with 10 μM MG132 (Peptides International), 100 μM cycloheximide (Sigma), 10-15 μM GGTi-2418 (Moffit Cancer Center), 10 μM Eeyarestatin 1 (Eer1, TOCRIS bioscience), 100 μM histamine (Sigma), 10 μM MRS2578 (Sigma), 100 μM ATP (Sigma), and 1 μM cyclosporin-A (Sigma).

### Tandem affinity purification and mass spectrometry

HEK293T cells were transiently transfected with Flag-HA-tagged FBXL2 or control plasmids using polyethylenimine (PEI). Twenty-four hours after transfection, cells were treated with MG132 (10 μM) for 3 h before harvesting. Immunoprecipitation and subsequent mass spectrometry was carried out as previously described^9,14,15^. The original mass spectrometry data can be accessed through the Stowers Original Data Repository at ftp://odr.stowers.org/LIBPB-484.

### Gene silencing

For gene silencing, cells were seeded approximately 24 h before transfection. The following ON-TARGET*plus* siRNA oligos from Dharmacon were transfected (5–15 nM) with HiPerfect for 24–48 h, according to the manufacturer's instructions (Qiagen): ON-TARGET*plus* human FBXL2 (oligo 1 GCACAGAACUGCCGAAACA, oligo 2 GCUCGGAAUUGCCACGAAU, oligo 3 J-0113562-07-0005); ON-TARGET*plus* human ITPR3 (L-006209-00-0005); ON-TARGET*plus* mouse ITPR3 (L-065715-01-0005) siRNA, ON-TARGET*plus* mouse PTEN (L-040700-02-0005); ON-TARGET*plus* human PTEN (L-003023-00-0005); and ON-TARGET non-targeting siRNA 1 (D-001810-01-05). To validate gene silencing by RT–PCR, total RNA was isolated using Qiagen's RNeasy kit (cat. no. 74104). The reverse transcription reaction was carried out in triplicate using 5 μg of total RNA using Oligo-dT primers with Superscript III RT polymerase (Invitrogen) according to the manufacturer's instructions. The real-time qPCR reaction was carried out using 250 ng cDNA using SYBR Green method with Roche Light Cycler 480II machine in a 96-well format. Data were analysed using 2nd derivative maximum with high confidence software for RT values according to the manufacturer's guidelines (Roche). Bar graphs represent the relative ratio of FBXL2 to GAPDH values. The following RT-primers were used: human FBXL2, forward: 5′-ATTTGACTGACGCAGGTTT-3′, reverse: 5′-GAGCTG GATGAGTGTGCTGT-3′; human GAPDH, forward: 5′-TGCACCACCAACT GCTTAGC-3′, reverse: 5′-GGCATGGACTGTGGTCATGAG-3′.

### Ubiquitination assays

Briefly, HEK-293T cells were co-transfected with HA- or GFP-tagged IP3R3 (either wild-type or an N-terminal fragment) and either Flag-tagged FBXL2 or an FBXL2(ΔF-box) mutant. Twenty-four hours after transfection, cells were incubated with MG132 for three hours before harvesting. FBXL2 (wild-type and mutant) was immunoprecipitated with anti-Flag M2 agarose beads (Sigma) and *in vitro* ubiquitination assays were carried out as previously described^9,16^. Flag-tagged trypsin-resistant tandem ubiquitin-binding entity (TR-TUBE), which directly binds polyubiquitin chains and protects them from proteasome-mediated degradation was used for cell-based assays, as previously described^17,18^.

### Fura-2 measurements

The cytosolic Ca^2+^ response was evaluated using the fluorescent Ca^2+^ indicator Fura-2/AM (Thermo Fischer Scientific). In brief, cells were grown on 24-mm coverslips and incubated at 37 °C for 30 min in 1 mM Ca^2+^ in Krebs-Ringer buffer (KRB: 135 mM NaCl, 5 mM KCl, 1 mM MgSO_4_, 0.4 mM K2HPO4, 5.5 mM glucose, 20 mM HEPES) supplemented with 2.5 mM Fura-2/AM, 0.02% Pluronic F-68 (Sigma-Aldrich), and 0.1 mM sulfinpyrazone (Sigma-Aldrich). Cells were then washed and supplied with 1 mM Ca^2+^/KRB. Next, cells were placed in an open Leyden chamber on a 37 °C thermostated stage and exposed to 340/380 nm wavelength light using the Olympus xcellence (Olympus) multiple wavelength high-resolution fluorescence microscopy system equipped with an Hamamatsu ORCA ER CCD camera (Hamamatsu Photonics) and a Upl FLN 40× oil objective (Olympus) to determine the cytosolic Ca^2+^ response. The photo-activation of aluminium phthalocyanine chloride was obtained using an excitation filter ET576/25 (Semrock), with 500 ms of excitation every cycle. Cytosolic Ca^2+^ concentration was calculated as previously described^19,20^.

### FRET-based measurements of mitochondrial Ca^2+^

Single-cell measurements of mitochondrial Ca^2+^ were performed in A549 cells or A549 knock-in clones transfected with 4mtD3cpv (ref. 6). After 36 h, cells were imaged using a Zeiss Axiovert 200 M microscope with a cooled CCD camera (Photometrics), which was equipped with a C-apochromatic 40 × /1.2 W CORR objective and controlled by MetaFluor 7.0 software (Universal Imaging). Emission ratio imaging of the 4mtD3cpv was achieved using a 436DF20 excitation filter, a 450 nm dichroic mirror, and two emission filters (475/40 for ECFP and 535/25 for citrine) that were controlled by a Lambda 10-2 filter changer (Sutter Instruments). The acquired fluorescence images were corrected for the background. The exposure times were typically 100-200 ms, and images were collected every second per wavelength. The photo-activation of aluminium phthalocyanine chloride was achieved using an excitation filter ET650/50 (Chroma Technology) with 500 ms of excitation every FRET ratio cycle.

### Aequorin measurements

Cells were transfected with the mtAEQ chimaera alone or together with constructs expressing FBXL2 or IP3R3. All aequorin measurements were carried out in KRB buffer supplemented with 1 mM CaCl_2_. Agonists and other drugs were added to the same medium, as specified in the figure legends. The experiments were terminated by lysing cells with 100 μM digitonin in a hypotonic Ca^2+^-rich solution (10 mM CaCl_2_), thus discharging the remaining aequorin pool. The light signal was collected and calibrated into [Ca^2+^] values, as previously described^21^.

### Experimental animals

Procedures involving animals and their care conformed with institutional guidelines, and all experimental protocols were approved by the animal ethics committee. NOD/SCID gamma (NSG) mice were housed in sterile conditions within high-efficiency particulate arrestance filtered micro-isolators, and fed with irradiated food and acidified water. Six-week-old male NSG mice were injected subcutaneously (s.c.) with 2 × 10^6^ cells. When tumour mass became palpable in successfully engrafted mice (around 36 days after the injection of A549 cells and around 70 days after the injection of PC3 cells), animals were randomly divided into different groups and subjected to various treatments as indicated in the figures. Where indicated, mice were subject to two rounds of GGTi-2418 treatment (50 mg kg^−1^) by intratumoural injection for five consecutive days. Tumour growth was monitored daily, and tumour diameters were measured with callipers every other day. The tumour volume was calculated using the following equation: volume = π/6 × *(a* × *b*^2^), where *a* is the major diameter and *b* is the minor diameter. At the end of the experiment, tumour progression was confirmed by either retro-orbital or intravenous injection of fluorescently labelled IRDye 2-deoxyglucose (2-DG), which was detected 24 h after injection using a Pearl Trilogy Imaging System (Li-Cor). All mice that reached the endpoint of the experiment (60 days for A549 or 92 days for PC3 cells) were euthanized and, subsequently, tumours were excised, weighted, and either immunoblotted or sectioned for immunofluorescence.

### Detection of cell death in cell systems

For cell death induction, cells were treated with different apoptotic stimuli as indicated in the text and figure legends. Apoptosis was determined by three different methods: (i) by blotting for different cell death markers, such as cleaved PARP and cleaved CASPASE-3; (ii) by analysing cytochrome *c* release; and (iii) by automated nuclei count analysis. For analysis of cytochrome *c* release, cells were fixed with 4% paraformaldehyde in PBS for 20 min, washed three times with PBS and then incubated for 10 min in PBS. Cells were then permeabilized with 0.1% Triton X-100 in PBS, followed by a 1-h wash with 2% milk in PBS. Cells were then incubated overnight at 4 °C in a wet chamber with a mouse anti-cytochrome *c* antibody followed by incubation with an Alexa 594 goat anti-mouse antibody and DAPI for 1 h at room temperature. After antibody incubation, cells were washed three times with PBS. Images were acquired on an Olympus ScanˆR station using a laser based autofocus and an image-based autofocus. Eighty fields were acquired for each well using a 20× magnification objective, NA 0.75. The different fluorophores were excited by an MT20 illumination system with 377/50, 595/30 excitation filters. Images were collected using an Orca-R2 CCD camera (Hamamatsu Photonics), without binning. The mean fluorescence intensities and standard deviations of nuclear cytochrome *c* were evaluated in comparison to corresponding controls. Automated nuclei count analysis was performed by seeding 50,000 cells on a 25-mm coverslip. Cells were grown for 48 h before treatment with H_2_O_2_ (1–2 mM for 4–16 h as indicated in figure legends) or etoposide (50 μM for 5 h). Coverslips were stained with Hoechst 10 μM, placed in an incubated chamber with controlled temperature, and mounted on a Zeiss Axiovert 200 M microscope equipped with a motorized stage. Images of nuclei (ranging in size from 5–25 μm) were acquired with a 10× Fluor objective (Zeiss) and a CoolSnap HQ CCD camera. Twenty random fields were acquired using the random stage scan tools in MetaMorph and analysed with the nuclei count application.

### Detection of cell death *in vivo*

After a retro orbital injection of 100 μl of CAS-MAP NIR probe (Vergent Bioscience), the reagent was allowed to circulate in mice for 30 min before analysis. Fluorescent *in vivo* images were acquired using a Pearl Trilogy Imaging System (Li-Cor). For the analysis of apoptosis in tumour tissue sections, after a retro orbital injection of 100 μl of SR-FLIVO probe (Immunochemistry Technology), the reagent was allowed to circulate in mice for 30 min. Tumours were excised, frozen, sectioned, and stained for nuclei using DRAQ5, according to the manufacturer's protocol (Cell Signaling Technology). After staining, the samples were mounted on coverslips and analysed using a Zeiss LSM 510 confocal microscope equipped with a Fluor 40 ×/1.30 NA oil-immersion objective. The acquired images were background corrected, and signals were analysed using Fiji software (available at http://fiji.sc/Fiji).

### Sub-cellular fractionation

Cells (approximately 10^9^) were harvested, washed in phosphate-buffered saline medium, pelleted by centrifugation at 500*g* for 5 min, re-suspended in homogenization buffer (0.25 M sucrose and 10 mM HEPES pH 7.4) and gently disrupted by dounce homogenization. The homogenate was centrifuged twice at 600*g* for 5 min to remove cellular debris and nuclei, and the supernatant was centrifuged at 10,300*g* for 10 min to pellet crude mitochondria. The resultant supernatant was centrifuged at 100,000*g* for 1 h in a Beckman 70 Ti rotor at 4 °C to pellet microsomes, which were re-suspended in homogenization buffer^22^. The quality of the preparation was confirmed by immunoblot analysis using different markers for the fractions obtained (that is, calnexin as endoplasmic reticulum marker, and β-actin as cytosolic markers).

### Immunohistochemistry in human tissue specimens

**Figure 2:**
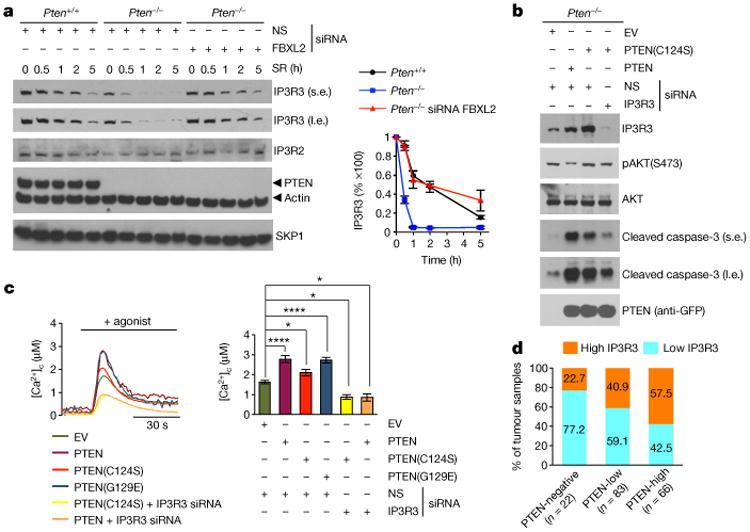
PTEN loss promotes IP3R3 degradation via FBXL2, and the expression of IP3R3 and PTEN directly correlate in human prostate cancer **a**, During 72 h of serum starvation, *Pten*^+/+^ and *Pten*^−/−^ MEFs were transfected with the indicated siRNAs, then re-stimulated with serum, and harvested at the indicated times for immunoblotting. l.e., long exposure; s.e., short exposure. The graph shows the quantification of IP3R3 levels from three independent experiments. Error bars indicate s.e.m. **b**, *Pten*^−/−^ MEFs were transfected with the indicated siRNAs and, after 48 h, with the indicated constructs. After 24 h, cells were harvested and WCLs were immunoblotted as indicated. **c**, *Pten*^−/−^ MEFs were transfected with the indicated siRNAs and, after 48 h, with the indicated constructs. Concentrations of cytosolic Ca^2+^ were measured with aequorin in response to ATP. Left, representative traces. Right, quantification of three independent experiments. *P* values were calculated by oneway ANOVA and multiple-comparisons test. Error bars indicate s.e.m. **d**, The graph shows the percentage of prostate tumours with no, low or high PTEN expression that have low or high IP3R3 expression. Linear regression was determined using *χ*^2^ test *r*^2^ = 0.04041, *P* = 0.0136. Unless otherwise noted, experiments were performed at least three times. For gel source data, see Supplementary Fig. 1.

Expression of PTEN and IP3R3 was assessed by immunohistochemistry using eight available tissue microarrays (TMAs) and one commercially available TMA. The eight TMAs were built generating triplicate cores from radical prostatectomy cases as previously described^23^, and included 89 prostate adenocarcinomas. The commercially available TMA was from USBiomax and included 60 prostate adenocarcinomas. Five-micrometre sections were deparaffinized and subjected to standard avidin-biotin-based immunohistochemistry procedures as reported in ref. 24. Primary antibodies were anti-PTEN (rabbit monoclonal, Cell Signaling Technology, no. 9559, 1:200 dilution) and anti-IP3R3 (rabbit polyclonal, Bethyl Laboratories, Inc., no. IHC-00639, 1:500 dilution). TMAs were scored by determining the percentage of epithelial prostate cells with immunoreactivity (0 = no expression, 1 = mild/ moderate expression, 2 = high and very high expression) for the protein of interest per tissue core. A score was generated for each tissue core by multiplying the percentage of expression by the intensity of expression. The average values of the representative cores from each patient sample were obtained and the median was used as a cut-off to transform protein expression into a qualitative variable. Cases were classified into three categories: ‘negative’ when score was 0, ‘low expression when score was lower than the median and ‘high expression when score was higher than the median. Linear regression analysis was performed with the GraphPad Prism 7.02 software with *χ*^2^ test. Original data are presented in Source Data for Fig. 2d.

### Live-cell imaging

HeLa cells were grown in dishes with a glass base (Thermo Scientific no. 150682) in DMEM medium supplemented with 10% FBS. Cells were transfected with GFP-FBXL2 cDNA using Lipofactemine 3000 reagent. Two hours post-transfection, cells were incubated in fresh medium containing GGTi-2418 (15 μM) for 16 h at 37 °C supplemented with 5% CO_2_. Live-cell imaging was carried out with a Zeiss LSM-510 META confocal microscope using a 63× oil-based objective in an incubation chamber at 37 °C supplemented with 5% CO_2_. Images were captured and processed with ZEN/ZEN lite imaging software from Zeiss.

### CRISPR genome editing

**Extended Data Figure 1:**
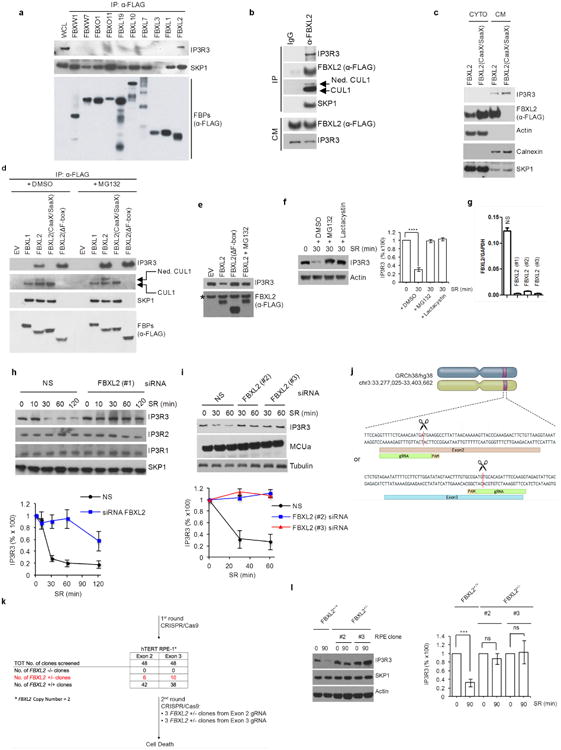
IP3R3 is targeted for proteasomal degradation by FBXL2 **a**, HEK293T cells were transfected with the indicated Flag-tagged F-box proteins (FBPs). 24 h post-transfection, cells were treated with MG132 for 3 h before harvesting for immunoprecipitation and immunoblotting as indicated. WCL, whole-cell lysate from untransfected cells. **b**, Flag-tagged FBXL2 was stably expressed in HeLa cells. After harvesting, cells were lysed, and cell membranes (CM) were isolated and immunoprecipitated with either a normal mouse IgG or an anti-Flag antibody. Subsequently, immunoprecipitates were analysed by immunoblotting as indicated. The results of these experiments (*n* = 2) indicate that FBXL2 is incorporated into a complete SCF ligase that binds its substrate(s) at cellular membranes. **c**, Flag-tagged FBXL2 or Flag-tagged FBXL2(CaaX/SaaX) were transiently expressed in HeLa cells. After harvesting, cells were lysed and cytoplasmic (CYTO) and cell membrane (CM) fractions were isolated and analysed by immunoblotting as indicated. Actin and calnexin are used as markers for cytoplasmic and cell membrane fractions, respectively. This experiment was performed twice. **d**, HEK293T cells were transfected with either an empty vector (EV) or the indicated Flag-tagged proteins. 24 h post-transfection, where indicated, cells were treated with MG132 for 3 h before harvesting for immunoprecipitation and immunoblotting. Ned. CUL1, neddylated CUL1. This experiment shows that FBXL2, but not FBXL2(CaaX/SaaX), interacts with endogenous, neddylated CUL1. Since the covalent linkage of NEDD8 to CUL1 stimulates the ubiquitin ligase activity of SCFs and is promoted by the binding of the substrate to the F-box protein subunit, this result suggests that FBXL2 localization to cell membranes is required for substrate binding, which in turn stimulates CUL1 neddylation and SCF activation. Moreover, FBXL2(ΔF-box) bound more IP3R3 than wild-type FBXL2, and this difference could be abolished by treatment with MG132, supporting the hypothesis that FBXL2(ΔF-box) cannot mediate the degradation of IP3R3 since it does not form an active SCF complex. **e**, HEK293T cells were transfected with either an empty vector or the indicated Flag-tagged proteins. The experiment was performed in the presence or absence of MG132 as indicated. Whole-cell lysates were immunoblotted as indicated. The asterisk indicates a non-specific band. This experiment was performed twice. The fact that proteasome inhibitors prevented the decrease of IP3R3 levels upon re-addition of serum suggests that IP3R3 is degraded by the proteasome in response to mitogens, in agreement with previous studies reporting IP3Rs as substrates of the proteasome^26,27^. **f**, Normal, non-transformed, non-immortalized, human diploid fibroblasts (NHFs) (passage 2) were serum-starved for 72 h and then re-stimulated with serum (SR) for 30 min in the absence or presence of either MG132 (a proteasome inhibitor) or lactacystin (another proteasome inhibitor) as indicated. The graph on the right shows the quantification of IP3R3 levels from three independent experiments. *P* values were calculated by one-way ANOVA. Error bars indicate s.e.m. **g**, NHFs (passage 2) were transfected with either three different siRNAs targeting FBXL2 (each independently) or a non-silencing siRNA (NS). The graph shows FBXL2 mRNA levels analysed using realtime PCR in triplicate measurements. Error bars indicate s.e.m. The values represent the ratios between FBXL2 and GAPDH mRNAs. **h**, During a 72 h serum starvation, NHFs (passage 2 or 3) were transfected with either an siRNA targeting FBXL2 (#1) or a non-silencing siRNA (NS). Cells were subsequently stimulated with medium containing serum and harvested at the indicated times for immunoblotting. The graph shows the quantification of IP3R3 levels from three independent experiments. Error bars indicate s.e.m. **i**, During a 72 h serum starvation, NHFs (passage 3 or 4) were transfected with either siRNAs targeting FBXL2 (oligo #2 or #3) or a non-silencing siRNA (NS). Cells were subsequently stimulated with medium containing serum and harvested at the indicated time points for immunoblotting. The graph shows the quantification of IP3R3 levels from three independent experiments. Error bars indicate s.e.m. **j**, Schematic representation of the *FBXL2* genomic locus and gRNAs target location. Exon 2 and exon 3 refer to the human *FBXL2* gene in NC_000003.12 (GRCh38.p7 (Gene Bank ID: 3129728)). **k**, Schematic representation of *FBXL2* CRISPR–Cas9 mutagenesis outcomes. A first round of CRISPR– Cas9 gene editing yielded no homozygous *FBXL2*-knockout clones. A secondary round of CRISPR–Cas9 gene editing was carried out in three *FBXL2*^+/−^ hTERT RPE-1 clones, which resulted in cell death, suggesting that *FBXL2* is required for cell fitness and it is not possible to generate *FBXL2*-knockout cells. Similar results were obtained in A549 cells (data not shown). **l**, *FBXL2*^+/+^ and *FBXL2*^+/−^ RPE-1-hTERT cells (clones 2 and 3) were serum-starved for 72 h and subsequently stimulated with medium containing serum for 90 min, after which cell extracts were immunoblotted for the indicated proteins. The graph shows the quantification of IP3R3 levels from three independent experiments. Unless otherwise noted, experiments were performed at least three times. For gel source data, see Supplementary Fig. 1.

To generate *ITPR3* Q550A;F553A;R554A, an optimal gRNA target sequence closest to the genomic target site and a 2 kb homologous recombination (HR) donor template were designed using the Benchling CRISPR Genome Engineering tool. The HR donor template was designed to introduce alanine substitutions at position Q550, F553 and R554, silent base-pair mutations to disrupt the PAM site, and an XhoI restriction site for bulk population screening, and was purchased as a synthetic gene from IDT. *ITPR3* gRNA target sequence (see Extended Data Fig. 7a) was cloned into pSpCas9(BB)-2A-GFP (PX458), a gift from F. Zhang (Addgene plasmid no. 48138). Similarly, to generate *FBXL2* deletions, two optimal gRNA target sequences (see Extended Data Fig. 1f) closest to the genomic target sites in either exon 2 or exon 3 were designed using the Benchling CRISPR Genome Engineering tool and cloned into the pSpCas9(BB)-2A-GFP (PX458) (ref. 25). RPE-1, A549 and PC3 cells were seeded into 10-cm dishes at approximately 70% confluency, and transfected with 5 μg of gRNA-containing PX458 plasmid and HR donor template, using lipofectamine 3000 (Life Technologies). The transfection was performed according to the manufacturer's recommended protocol, using a 2:1 ratio of lipofectamine:DNA. Two days after transfection, GFP-positive cells were sorted using the Beckman Coulter MoFlo XDP cell sorter (100 μm nozzle), and 15,000 cells were plated on a 15 cm dish. For *ITPR3* Q550A;F553A;R554A knock-in, a GFP-sorted population sample was also collected for subsequent bulk population genotyping by amplification of the target region and digestion with XhoI. 8–10 days later, single cell clones were picked, trypsinized in 0.25% Trypsin-EDTA for 5 min, and plated into individual wells of a 96-well plate for genotyping. Genomic DNA was collected using QuickExtract (Epicentre). Genotyping PCRs were performed with MangoTaq DNA Polymerase (Bioline), using primers surrounding the genomic target sites (see Extended Data Fig. 1f and Extended Data Fig. 7a). The resulting PCR products were then purified. For *ITPR3* Q550A;F553A;R554A knock-in, PCR products were also digested with XhoI. Positive clones were sequenced to determine the presence of a indel event (*FBXL2* knockout) or complete recombination event (*ITPR3* Q550A;F553A;R554A knock-in). To further validate the mutational status of candidate clones, the PCR products were subjected to TOPO-TA Cloning (Invitrogen), and sequenced in order to distinguish the amplified products of distinct alleles. Fifty bacterial colonies for each TOPO-TA cloning reaction were sequenced and aligned to the corresponding wild-type template in Benchling to confirm that all alleles were correctly targeted.

### Statistics analyses

All data were analysed by Prism 6 (GraphPad). Unless otherwise noted in figure legends, data are representative of at least three biologically independent experiments. Two-group datasets were analysed by Student's unpaired *t*-test. For three or more group analysis, one-way ANOVA Tukey's multiple comparison test was used. Linear regression analysis was performed with the GraphPad Prism 7.02 software *χ*^2^ test. One asterisk was used for *P* < 0.05, two asterisks for *P* < 0.01, three asterisks for *P* < 0.001, and four asterisks for *P* < 0.0001. Statistical analyses from independent experiments are reported in the Source Data files and in Supplementary Table 1.

### Data availability

Most data generated or analysed during this study are included in this published article and its supplementary information files. Additional datasets generated during and/or analysed during the current study and relevant information are available from the corresponding authors upon reasonable request.

## Extended Data

**Extended Data Figure 2:**
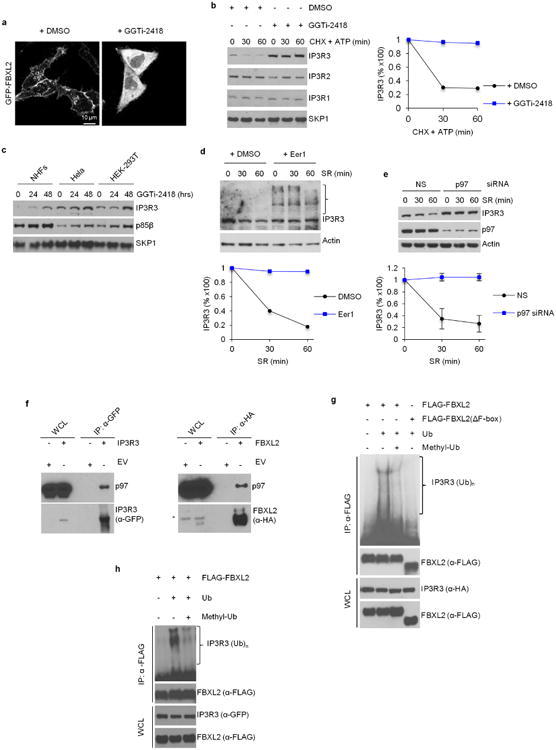
The degradation of IP3R3 is dependent on FBXL2 localization to cell membranes and on the segregase activity of p97 **a**, HeLa cells were transfected with GFP-tagged FBXL2 and then treated with either DMSO or GGTI-2418 for 16 h. Live cell imaging was carried out with an LSM510 confocal microscope using a 63× objective. Scale bars, 10 μm. **b**, NHFs were incubated with GGTi-2418 for 30 h and then with cycloheximide (CHX) and ATP. Cells were subsequently harvested at the indicated times for immunoblotting. The graph shows the quantification of IP3R3 levels from two independent experiments. **c**, NHFs (passage 3), HeLa and HEK293T cells were incubated with GGTi-2418 for the indicated times. Cells were subsequently harvested for immunoblotting. This experiment was performed once. **d**, NHFs (passage 3) were serum-starved for 72 h, treated with either DMSO or Eer1, and then re-stimulated with serum (SR) for the indicated times. The graph shows the quantification of IP3R3 levels from two independent experiments. The bracket on the right marks a ladder of bands which, presumably, are ubiquitinated species of IP3R3 that are not degraded when p97 is inhibited. **e**, During a 72 h serum starvation, NHFs (passage 3 and 4) were transfected with either an siRNA targeting p97 or a non-silencing siRNA (NS). Cells were subsequently stimulated with medium containing serum and harvested at the indicated time points for immunoblotting. The graph shows the quantification of IP3R3 levels from three independent experiments. Error bars indicate s.e.m. These results, together with those shown in **d**, are in agreement with the findings that IP3Rs are ubiquitinated while they are membrane-associated, and those showing that p97 promotes the degradation of IP3Rs^27,28^. Thus, we propose that, after its FBXL2-mediated ubiquitination, IP3R3 is extracted from cell membranes by the segregase activity of p97 to be degraded by the proteasome. **f**, HEK293T cells were transfected with either an empty vector (EV) or the indicated GFP-tagged and HA-tagged proteins. 24 h post-transfection, cells were treated with MG132 for 3 h before harvesting for immunoprecipitation and immunoblotting as indicated. This experiment was performed twice. **g, h**, HEK293T cells were transfected with Flag-tagged FBXL2 and the indicated versions of tagged IP3R3. After immunopurification with an anti-Flag resin, *in vitro* ubiquitination of IP3R3 was performed in the presence of UAE1, Ubch3, Ubch5 and ubiquitin (Ub). Where indicated, an excess of methylated ubiquitin (methyl-Ub), which blocks chain extension, was added to the *in vitro* reactions. The presence of methyl-Ub resulted in the disappearance of the highest molecular weight forms of IP3R3, demonstrating that the high molecular weight forms of IP3R3 are indeed polyubiquitinated species of the protein. Samples were analysed by immunoblotting with the indicated antibodies. The bracket on the right marks a ladder of bands corresponding to ubiquitinated IP3R3. Immunoblots of whole-cell lysates (WCL) are shown at the bottom. Unless otherwise noted, experiments were performed at least three times. For gel source data, see Supplementary Fig. 1.

**Extended Data Figure 3:**
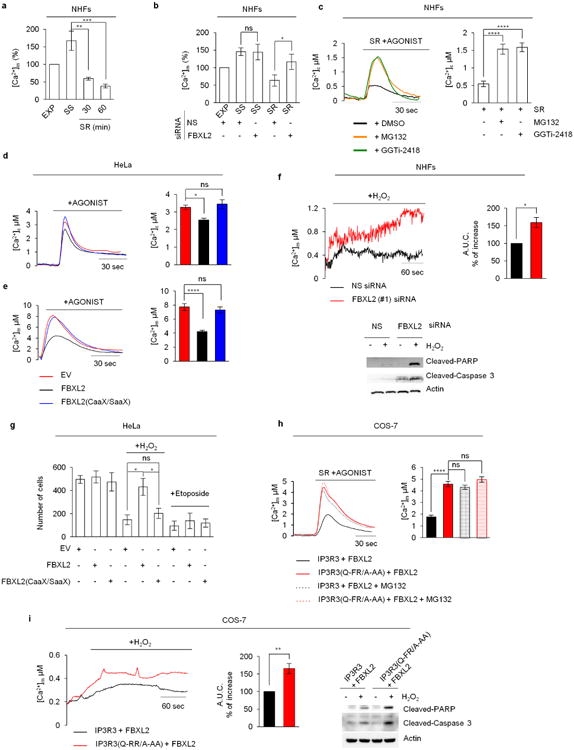
FBXL2 controls Ca^2+^ mobilization and Ca^2+^-mediated apoptosis **a**, Concentrations of mitochondrial Ca^2+^ were measured with mitochondria-targeted aequorin in response to agonist stimulation (ATP, a purinergic GPCR (G-protein-coupled receptor) agonist) in exponentially growing (EXP), serum-starved (SS), and serum re-stimulated (SR) NHFs (passage 4 or 5). Quantification of three independent experiments is shown and represented as percentage change compared to EXP cells, which were set as 100%. *P* values were calculated by one-way ANOVA and multiple-comparisons test. Error bars indicate s.e.m. **b**, Concentrations of mitochondrial Ca^2+^ were measured as in **a** in exponentially growing (EXP), serum-starved (SS), and serum re-stimulated for one hour (SR) NHFs (passage 5) transfected with either an siRNA targeting FBXL2 (#1) or a non-silencing siRNA (NS). Quantifications and *P* value analyses was performed as in **a**. **c**, Concentrations of cytosolic Ca^2+^ were measured with aequorin in response to agonist stimulation (ATP) in NHFs (passage 2) re-stimulated with serum for one hour (SR) in the absence or presence of MG132 or GGTi2418. On the left, representative traces. On the right, quantification of three independent experiments. *P* values were calculated by a one-way ANOVA and multiple-comparisons test. Error bars indicate s.e.m. **d, e**, HeLa cells were transfected with either an empty vector (EV), FBXL2 or FBXL2(CaaX/SaaX). Concentrations of cytosolic (**d**) and mitochondrial (**e**) Ca^2+^ were measured with the appropriate aequorin in response to agonist stimulation (ATP). On the left, representative traces. On the right, quantifications of three independent experiments. *P* values were calculated by one-way ANOVA and multiple-comparisons test. Error bars indicate s.e.m. **f**, Concentrations of mitochondrial Ca^2+^ were measured with mitochondrial targeted aequorin upon treatment with H_2_O_2_ in NHFs (passage 2) transfected with either an siRNA targeting FBXL2 (#1) or a non-silencing siRNA (NS). On the left, representative traces. On the right, quantifications of areas under the curve (AUC) are represented as percentage increase compared to NS-transfected cells, which were set as 100%. *P* values were calculated by unpaired *t*-test. Error bars indicate s.e.m. At the bottom, immunoblots of cell lysates of a representative experiment upon treatment with H_2_O_2_ for 3 h. **g**, HeLa cells transfected with either an empty vector (EV), FBXL2 or FBXL2(CaaX/SaaX) were treated with either H_2_O_2_ (for 5 h) or etoposide (for 5 h). Induction of cell death was evaluated using automated nuclei count analysis of twenty randomly chosen fields. *P* values were calculated by one-way ANOVA and multiple-comparisons test. Error bars indicate s.e.m. **h**, COS-7 cells expressing either IP3R3 or IP3R3(Q-FR/A-AA) in combination with Flag-tagged FBXL2 were serum-starved for 20 h, re-stimulated with serum (SR) for 4 h with or without MG132, as indicated, and treated with ATP. Left, representative traces showing concentrations of mitochondrial Ca^2+^ measured with mitochondrial targeted aequorin. Right, quantification of three independent experiments. *P* values were calculated by one-way ANOVA and multiple-comparisons test. Error bars indicate s.e.m. **i**, Concentrations of mitochondrial Ca^2+^ were measured with mitochondria-targeted aequorin upon treatment with H_2_O_2_ in COS-7 cells expressing either IP3R3 or IP3R3(Q-FR/A-AA) in combination with Flag-tagged FBXL2. Left, representative traces. Middle, quantifications of areas under the curve represented as percentage increase compared to NS-transfected cells, which were set as 100%. *P* values were calculated using an unpaired *t*-test. Error bars indicate s.e.m. Right, immunoblots of cell lysates of a representative experiment upon treatment with H_2_O_2_ for 3 h. Unless otherwise noted, experiments were performed at least three times. For gel source data, see Supplementary Fig. 1.

**Extended Data Figure 4:**
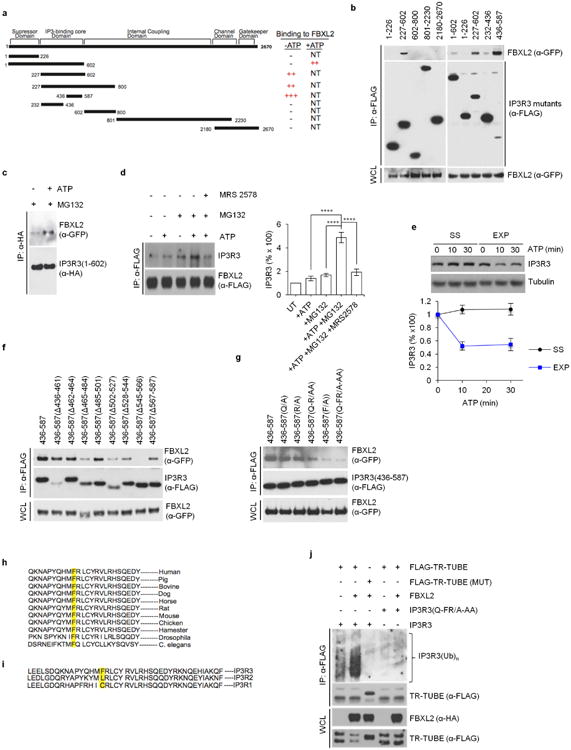
Mapping of the FBXL2-binding domain in IP3R3, and evidence that the N-terminal suppressor domain inhibits the IP3R3-FBXL2 interaction **a**, Schematic representation of IP3R3 mutants. Binding of IP3R3 to FBXL2 is indicated with the symbol (+). **b**, HEK293T cells were transfected with GFP-tagged FBXL2 and the indicated Flag-tagged IP3R3 truncated mutants. Whole-cell lysates (WCL) were immunoprecipitated (IP) with anti-Flag resin and proteins were immunoblotted as indicated. This experiment was performed twice. **c**, HEK293T cells were transfected with GFP-tagged FBXL2 and HA-tagged In IP3R3(1-602) constructs. Sixteen hours after transfection, cells were incubated with MG132 for 3 h before stimulation with ATP for 30 min. Whole-cell lysates were immunoprecipitated (IP) with an anti-HA resin and proteins were immunoblotted as indicated. This experiment was performed twice. **d**, HeLa cells stably transfected with Flag-tagged FBXL2 under the control of a doxycycline-inducible promoter were treated with doxycycline (0.4 μ*g* ml^−1^) for 16 h and incubated with or without MG132 (during the last 3 h), in the presence or absence of MRS 2578, an antagonist of P2Y6 receptor (during the last 90 min), as indicated. Cells were subsequently treated with or without ATP for 30 min. Whole-cell lysates were immunoprecipitated (IP) with an anti-Flag resin and proteins were immunoblotted as indicated. Right panel shows quantifications of IP3R3 levels compared to untreated cells (UT), which were set as 100%. *P* values were calculated by one-way ANOVA and multiple-comparisons test. Error bars indicate s.e.m. The fact (shown in **b**) that fragments encoding IP3R3(436–587) and IP3R3(227–602) interact with FBXL2 better than the IP3R3(1–602) fragment suggests that the N terminus of IP3R3 inhibits the interaction between FBXL2 and IP3R3. It has been shown that removal of the N-terminal suppressor domain (amino acids 1–226) increases the binding of IP3 to the IP3 binding core domain (amino acids 227–579), and IP3 binding evokes conformational changes that open the suppressor domain^29–32^. These conformational changes may allow FBXL2 to access the degron of IP3R3 (that is, the amino acid region required for binding to FBXL2). Thus, in agreement with previous data indicating that IP3 causes the ubiquitination and downregulation of IP3Rs (ref. 27), our results suggest that IP3 promotes the binding of FBXL2 to IP3R3 and that FBXL2 preferentially binds IP3R3 in its open conformation (upon IP3 binding). Accordingly, treatment of cells with ATP, which induces IP3 production and repositioning of the N-terminal suppressor domain, increased the binding between FBXL2 and IP3R3, particularly if proteasomal degradation was inhibited by MG132. This suggests that once IP3 (produced after ATP stimulation) unmasks the IP3R3 degron, FBXL2 binds IP3R3 and this interaction is preserved when FBXL2-mediated degradation of IP3R3 is inhibited. So, ATP and MG132 appear to synergize with each other in promoting and preserving the FBXL2–IP3R3 interaction, respectively. **e**, Serum-starved (SS) and exponentially (EXP) growing NHFs (passage 2 and 3) were treated with or without ATP as indicated. Cells were subsequently harvested at the indicated time points for immunoblotting. The graph shows the quantification of IP3R3 levels. Error bars indicate s.e.m. **f**, HEK293T cells were transfected with GFP-tagged FBXL2 and the indicated Flag-tagged IP3R3 deletion mutants (in the context of the 436–587 domain of IP3R3). Whole-cell lysates (WCL) were immunoprecipitated (IP) with anti-Flag resin, and proteins were immunoblotted as indicated. This experiment was performed twice. **g**, The experiment was performed twice as in **f**, except that different IP3R3 mutants were used. **h**, Alignment of the amino acid regions containing the FBXL2-binding motif in IP3R3 orthologues. **i**, Alignment of the amino acid regions containing the FBXL2-binding motif in human IP3R3 with the corresponding region in IP3R1 and IP3R2. **j**, HEK293T cells were transfected with the indicated constructs. Whole-cell lysates (WCL) were immunoprecipitated (IP) with anti-Flag resin and immunoblotted as indicated. The bracket on the right marks a ladder of bands corresponding to polyubiquitinated IP3R3. This experiment was performed twice. Unless otherwise noted, experiments were performed at least three times. For gel source data, see Supplementary Fig. 1.

**Extended Data Figure 5:**
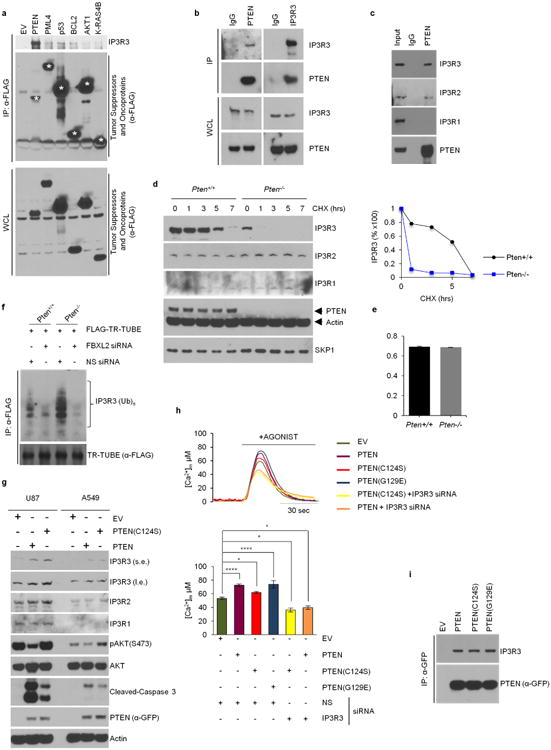
IP3R3 degradation is prevented by PTEN, independently of its phosphatase activity **a**, HEK293T cells were transfected with the indicated Flag-tagged constructs encoding tumour suppressors and oncoproteins with established roles in Ca^2+^ homeostasis and that are known to localize to the endoplasmic reticulum-mitochondria interface (AKT1, BCL2, p53, PML4, PTEN and KRAS4B). Of those, AKT1, BCL2, PML4 and PTEN are known IP3R3 interactors^33–39^. Twenty-four hours post-transfection, cells were harvested for anti-Flag immunoprecipitations and immunoblotting as indicated. Asterisks denote Flag-tagged proteins. This experiment was performed twice. **b, c**, Whole-cell lysates (WCL) from HEK293T cells were immunoprecipitated with the indicated antibodies and immunoblotted as indicated. This experiment was performed twice. **d**, *Pten^+/+^* and *Pten*^−/−^ MEFs were incubated with cycloheximide (CHX) for the indicated times. Cells were subsequently harvested for immunoblotting as indicated. The graph on the right shows the quantification of IP3R3 levels from two independent experiments. **e**, FBXL2 mRNA levels in *Pten^+/+^* and *Pten*^−/−^ MEFs analysed using real-time PCR in triplicate measurements (± s.e.m.). The values represent the ratios between FBXL2 and GAPDH mRNAs. This experiment was performed twice. **f**, *Pten^+/+^* and *Pten*^−/−^ MEFs were transfected for 48 h with either an siRNA targeting FBXL2 (#1) or a non-silencing siRNA (NS). Subsequently, MEFs were transfected with Flag-TR-TUBE cDNA. Whole-cell lysates (WCL) were immunoprecipitated (IP) with anti-Flag resin and immunoblotted as indicated. The bracket on the right marks a ladder of bands corresponding to polyubiquitinated IP3R3. This experiment was performed twice. **g**, U87 and A549 cells were transfected with either GFP-tagged PTEN, GFP-tagged PTEN(C124S), or an empty vector (EV) as indicated. Cells were subsequently harvested and whole-cell lysates were immunoblotted as indicated. Long (l.e.) and short (s.e.) exposures are shown for IP3R3. **h**, *Pten*^−/−^ MEFs were processed as in (Fig. 2c). Concentrations of mitochondrial Ca^2+^ were measured with mitochondria-targeted aequorin in response to agonist stimulation (ATP). Top, representative traces. Bottom, quantification of three independent experiments. *P* values were calculated by one-way ANOVA and multiple comparisons test. Error bars indicate s.e.m. **i**, HEK293T cells were transfected with either GFP-tagged wild-type PTEN or the indicated GFP-tagged cancer-associated PTEN mutants. Whole-cell lysates (WCL) were immunoprecipitated (IP) with anti-GFP resin and proteins were immunoblotted as indicated. This experiment was performed twice. Unless otherwise noted, experiments were performed at least three times. For gel source data, see Supplementary Fig. 1.

**Extended Data Figure 6:**
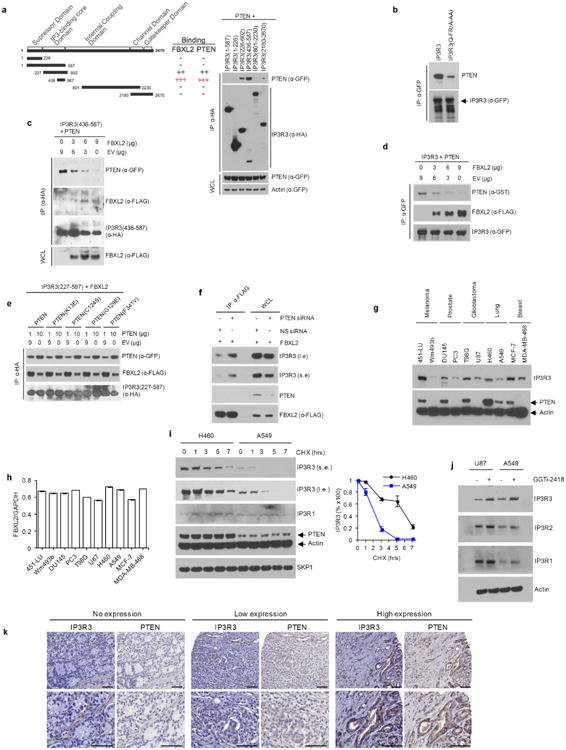
PTEN competes with FBXL2 for the binding to IP3R3, and the levels of IP3R3 and PTEN directly correlate in cancer cell lines and human prostate tumours **a**, Left, schematic representation of IP3R3 mutants used for binding site mapping. Binding of IP3R3 to FBXL2 and PTEN is indicated with the symbol (+). Right, HEK293T cells were transfected with GFP-tagged PTEN and the indicated HA-tagged IP3R3 truncated mutants. Whole-cell lysates (WCL) were immunoprecipitated (IP) with anti-HA resin and immunocomplexes were probed with antibodies to the indicated proteins. This experiment was performed twice. **b**, HEK293T cells were transfected with GFP-tagged IP3R3 or IP3R3(Q-FR/A-AA). Whole-cell lysates were immunoprecipitated (IP) with an anti-GFP resin and proteins were immunoblotted as indicated. c, HEK293T cells were co-transfected, as indicated, with GFP-tagged PTEN, HA-IP3R3(436–587), and increasing amounts of Flag-tagged FBXL2. Whole-cell lysates (WCL) were immunoprecipitated (IP) with anti-HA resin and proteins were immunoblotted as indicated. This experiment was performed twice. **d**, HEK293T cells were co-transfected, as indicated, with GST-tagged PTEN, GFP-tagged IP3R3, and increasing amounts of Flag-tagged FBXL2. Whole-cell lysates were immunoprecipitated (IP) with anti-GFP resin and proteins were immunoblotted as indicated. This experiment was performed twice. **e**, HEK293T cells were co-transfected, as indicated, with Flag-tagged FBXL2, HA-tagged IP3R3(436–587), and increasing amounts of either GFP-tagged PTEN or the indicated GFP-tagged cancer-associated PTEN mutants. Whole-cell lysates were immunoprecipitated (IP) with anti-HA resin and proteins were immunoblotted as indicated. This experiment was performed twice. **f**, HEK293T cells were transfected with either an siRNA targeting PTEN or a non-silencing siRNA (NS), as indicated. After 48 h, cells were transfected with Flag-tagged FBXL2. Sixteen hours after the second transfection, whole-cell lysates were immunoprecipitated (IP) with anti-Flag resin. Immunocomplexes and WCLs were immunoblotted as indicated. **g**, Whole-cell lysates from the indicated cancer cell lines were immunoblotted as indicated. This experiment was performed twice. **h**, FBXL2 mRNA levels in the indicated cell lines analysed using real-time PCR in triplicate measurements (± s.e.m.). The values represent the ratios between FBXL2 and GAPDH mRNAs. This experiment was performed once. **i**, H460 and A549 cells were incubated with cycloheximide (CHX) for the indicated times. Cells were subsequently harvested and processed for immunoblotting as indicated. Long (l.e.) and short (s.e.) exposures are shown for IP3R3. The graph shows the quantification of IP3R3 levels from three independent experiments. Error bars indicate s.e.m. **j**, U87 and A549 cells were treated with GGTi-2418 for 16 h where indicated. Cells were subsequently harvested and whole-cell lysates were immunoblotted as indicated. This experiment was performed twice. **k**, Representative immunohistochemistry staining images of human prostate tumour specimens with no, low or high levels of PTEN protein. Levels of IP3R3 in consecutive tissue slides are shown. Scale bars correspond to 50 μm. Unless otherwise noted, experiments were performed at least three times. For gel source data, see Supplementary Fig. 1.

**Extended Data Figure 7:**
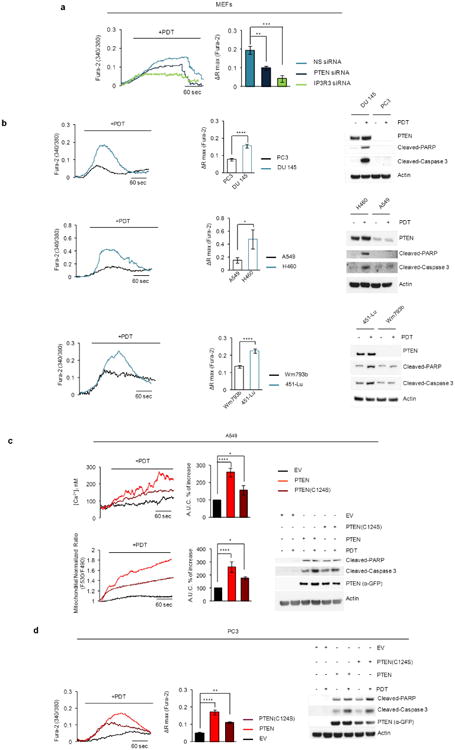
Both wild-type PTEN and a phosphatase dead PTEN mutant sensitize cells to photodynamic therapy **a**, MEFs were transfected for 48 h with either a non-silencing siRNA (NS) or siRNAs targeting PTEN or IP3R3 as indicated. Cells were loaded with Fura-2 dye for Ca^2+^ mobilization analysis upon treatment with PDT. Representative traces (left panel) show cytosolic calcium mobilization. Quantifications are shown in the right panel. *P* values were calculated by one-way ANOVA and multiple-comparisons test. ΔRmax, maximum variation in peak values of 340/380 ratiometric analysis. Error bars indicate s.e.m. **b**, Matched-pair cell lines expressing wild-type PTEN or displaying low or no PTEN expression (that is, DU145 and PC3 (prostate cancer cell lines); H460 and A549 (lung cancer cell lines); 451-LU and Wm493b (melanoma cell lines), respectively) were loaded with Fura-2 dye for Ca^2+^ mobilization analysis upon PDT treatment. Representative traces (left panels) show cytosolic calcium mobilization. Bar graphs (middle panels) show the quantification of three independent experiments. Right panels show corresponding whole cell extracts immunoblotted as indicated. *P* values were calculated by unpaired *t*-test. Error bars indicate s.e.m. **c**, A549 cells were transiently transfected with either GFP-tagged PTEN, GFP-tagged PTEN(C124S), or an empty vector (EV) as indicated. Cells were treated with phthalocyanine, a photosensitizer used for PDT in patients with cancer. Top panels show cytosolic Ca^2+^ concentrations measured with Fura-2. Bottom (left and middle panels), mitochondrial Ca^2+^ mobilization in cells expressing the Ca^2+^ sensitive probe 4mtD3cpv. Left panels show representative traces, and panels on their right show quantifications of areas under the curve represented as percentage increase compared to empty-vector-transfected cells, which were set as 100%. Right bottom panel shows immunoblots of cell lysates of a representative experiment. *P* values were calculated by oneway ANOVA and multiple-comparisons test. Error bars indicate s.e.m. **d**, PC3 cells were transiently transfected with either GFP-tagged PTEN, GFP-tagged PTEN(C124S), or an empty vector (EV) as indicated. Cells were loaded with Fura-2 dye for Ca^2+^ mobilization analysis upon PDT treatment. Representative traces show cytosolic calcium mobilization. Bar graphs show the quantification of three independent experiments. Right panel shows immunoblots of cell lysates of a representative experiment. *P* values were calculated by one-way ANOVA and multiple-comparisons test. Error bars indicate s.e.m. Unless otherwise noted, experiments were performed at least three times. For gel source data, see Supplementary Fig. 1.

**Extended Data Figure 8:**
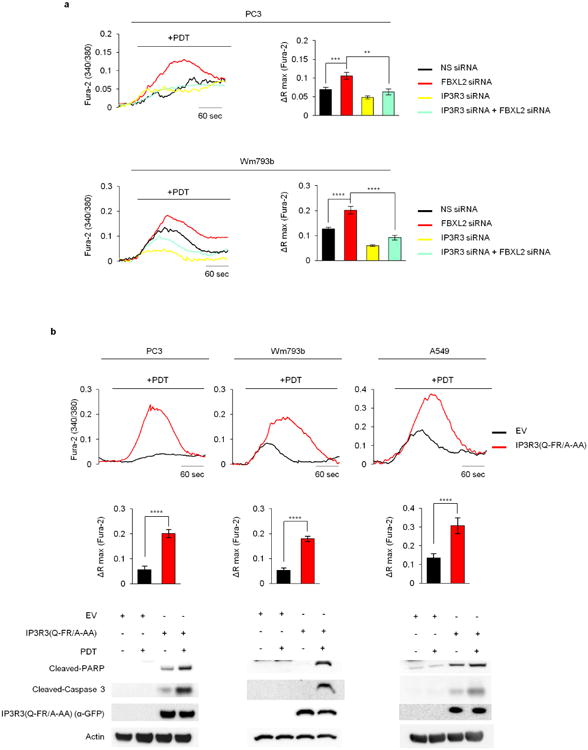
Stabilization of IP3R3 sensitizes cells to photodynamic therapy **a**, PC3 cells (top panels) and Wm493b cells (bottom panels) were transfected with a non-silencing siRNA (NS) or siRNAs targeting either FBXL2, IP3R3 or both FBXL2 and IP3R3. Cells were loaded with Fura-2 dye for Ca^2+^ mobilization analysis upon PDT treatment. Representative traces show cytosolic calcium mobilization. Bar graphs show the quantification of three independent experiments. ΔRmax, maximum variation in peak values of 340/380 ratiometric analysis. *P* values were calculated by one-way ANOVA and multiple-comparisons test. Error bars indicate s.e.m. **b**, PC3 cells (left panels), Wm493b cells (middle panels) and A549 cells (right panels) expressing either GFP-tagged IP3R3(Q-FR/A-AA) or an empty vector (EV) were loaded with Fura-2 dye for Ca^2+^ mobilization analysis upon PDT treatment. Representative traces show cytosolic calcium mobilization. Bar graphs show the quantification of three independent experiments. Statistical analysis was performed with unpaired *t*-tests. Error bars indicate s.e.m. Bottom panels show corresponding immunoblots of cell lysates of representative experiments. Unless otherwise noted, experiments were performed at least three times. For gel source data, see Supplementary Fig. 1.

**Extended Data Figure 9:**
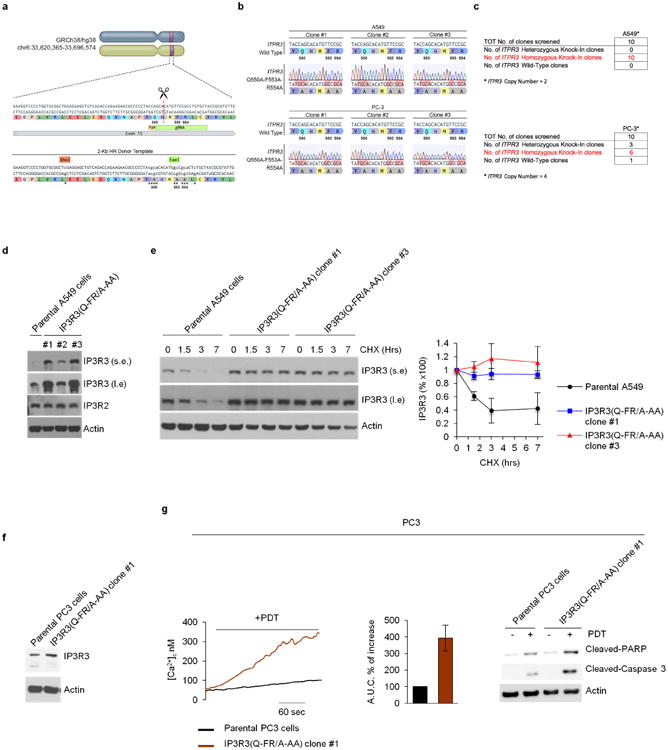
A non-degradable IP3R3 mutant sensitizes tumour cells to photodynamic therapy **a**, Schematic representation of the *ITPR3* genomic locus and gRNA target location. Exon 15 refers to human *ITPR3* gene (GRCh38.p7 (Gene Bank ID: 31297280)). Silent mutations are shown as lower case letters and indicated by asterisks. **b**, Wild-type genomic DNA template and knock-in mutant sequences identified by TOPO-TA cloning of *ITPR3* PCR from three independent A549 and PC3 clones are depicted. **c**, Schematic representation of the *ITPR3* CRISPR–Cas9 mutagenesis outcomes for A549 and PC3 cells. **d**, Whole-cell lysates from A549 parental cells and three independent IP3R3(Q-FR/A-AA) knock-in clones were immunobloted as indicated. **e**, A549 parental cells and IP3R3(Q-FR/A-AA) knock-in cells (clones 1 and 3) were incubated with cycloheximide (CHX) for the indicated times. Cells were subsequently harvested for immunoblotting as indicated. The graph shows the quantification of IP3R3 levels from three independent experiments. Error bars indicate s.e.m. **f**, Whole-cell lysates from PC3 parental cells and an IP3R3(Q-FR/A-AA) knock-in clone (#1) were immunoblotted as indicated. **g**, PC3 parental cells and the IP3R3(Q-FR/A-AA) knock-in PC3 clone 1 were treated with phthalocyanine, a photosensitizer used for PDT in patients with cancer. Left panel, cytosolic Ca^2+^ concentrations measured with Fura-2. Middle panel, quantifications of areas under the curve represented as percentage increase compared to empty-vector-transfected cells, which were set as 100%. Right panel, immunoblots of cell lysates from a representative experiment. The *P* value was calculated by unpaired *t*-test. Error bars indicate s.e.m. Unless otherwise noted, experiments were performed at least three times. For gel source data, see Supplementary Fig. 1.

**Extended Data Figure 10:**
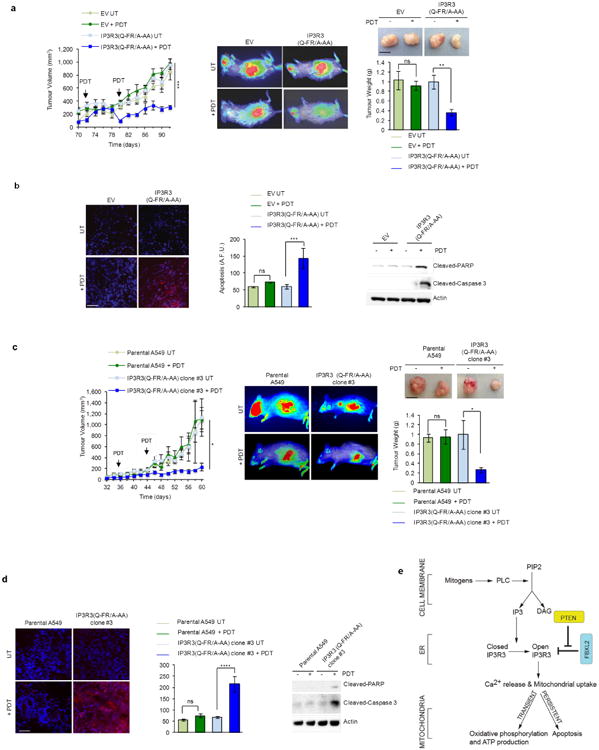
A non-degradable IP3R3 mutant and GGTi-2418 sensitize tumours to photodynamic therapy **a**, Tumour growth of PC3 cell xenografts analysed as in (Fig. 4a). Error bars indicate s.e.m. **b**, Apoptosis of PC3 cell xenografts analysed as in (Fig. 4b). Error bars indicate s.e.m. **c**, A549 parental cells and IP3R3(Q-FR/A-AA) knock-in clone no. 3 were processed as in (Fig. 4a). **d**, A549 parental cells and IP3R3(Q-FR/A-AA) knock-in clone no. 3 were processed as in (Fig. 4b). **e**, A model of the FBXL2- and PTEN-dependent regulation of IP3R3 function in energy production and cell death. In response to IP3 production, IP3R3 releases calcium from the endoplasmic reticulum (ER) to mitochondria, stimulating oxidative phosphorylation and ATP production. To avoid persistent calcium flux and consequent cell death, IP3R3 is degraded via FBXL2. PTEN competes with FBXL2 for IP3R3 binding, thus increasing the stability of IP3R3 and promoting apoptosis. The fact that PTEN(C124S), a catalytically dead mutant, binds IP3R3, competes with FBXL2 for IP3R3 binding, and stabilizes IP3R3 in a manner that is identical to wild-type PTEN, strongly indicates that neither the lipid phosphatase activity nor the protein phosphatase activity of PTEN are required to positively affect IP3R3 stability. PTEN(G129E), a mutant displaying a greatly reduced lipid phosphatase activity, but retaining protein phosphatase activity, also binds IP3R3 and competes with FBXL2 in a manner that is indistinguishable from wild-type PTEN. However, PTEN(C124S) induces an effect on Ca^2+^ mobilization that is significant, but not as high as that evoked by wild-type PTEN and PTEN(G129E). This suggests that, in addition to its phosphatase-independent ability to stabilize IP3R3,the protein phosphatase activity of PTEN may contribute to Ca^2+^ flux, as suggested by Bononi *et al*.^40^. Our findings reveal the molecular basis (that is, the competition with FBXL2 for IP3R3 binding) by which PTEN(C124S) is able to promote both a mitochondrial Ca^2+^ response and apoptosis. Importantly, according to this model, FBXL2 is a pro-survival factor, which complements its known role in the efficient activation of the PI3K cascade^9^. Finally, our results show that both FBXL2 and PTEN do not affect the levels and stability of IP3R1 and IP3R2. We note that one peptide corresponding to IP3R2 was identified in the original purification of the FBXL2 complex (ftp://odr.stowers.org/LIBPB-484). Moreover, the FBXL2 complex purified by the Harper group contained one peptide corresponding to IP3R1 (ref. 41). FBXL2 binds both p85α and p85β; but it targets only p85β for degradation^9^. We speculate that the binding to p85α is indirect and occurs because of the presence in the cell of p85α-p85β heterodimers. Since IP3R1, IP3R2, and IP3R3 also form heteromers^42–44^, it is possible that FBXL2 indirectly binds one or both IP3R3 paralogues, but only targets IP3R3 for degradation. Unless otherwise noted, experiments were performed at least three times. For gel source data, see Supplementary Fig. 1.

## Supplementary Material

Supplemental 1

Supplemental 2

## Figures and Tables

**Figure 1:**
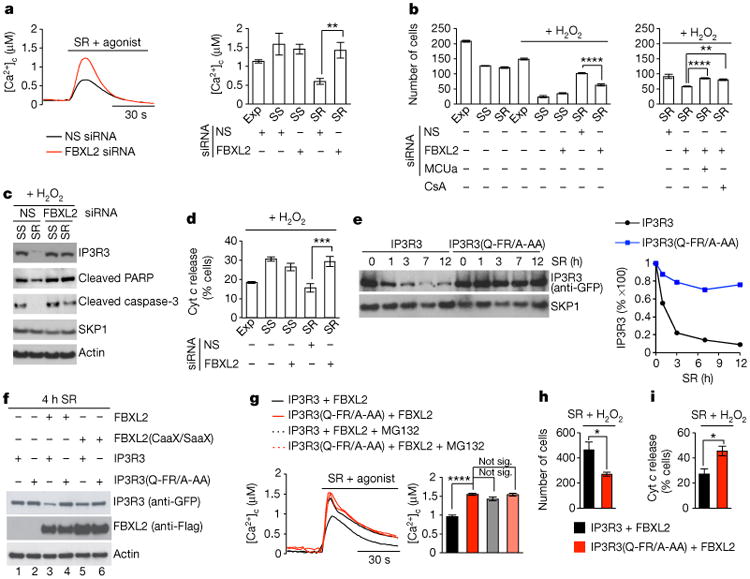
FBXL2-mediated degradation of IP3R3 controls Ca^2+^ flux and sensitivity to apoptosis **a**, Concentrations of cytosolic Ca^2+^ ([Ca^2+^]_c_) were measured with aequorin in response to agonist stimulation (ATP) in NHFs (passage 2 and 3) exponentially growing (Exp), serum-starved (SS), or re-stimulated with serum (SR), which were transfected with an siRNA targeting FBXL2 or a non-silencing (NS) siRNA. Left, two representative traces. Right, quantification of three independent experiments. *P* values were calculated by one-way ANOVA and multiple-comparisons test. Error bars indicate s.e.m. **b-d**, Apoptosis was evaluated after treatment with H_2_O_2_ using automated nuclei count analysis of twenty randomly chosen fields following a 16 h treatment (**b**), immunoblot detection of cleaved PARP and cleaved caspase-3 following a 3 h treatment (**c**), and automated analysis of cells with released cytochrome *c* (Cyt *c*) from 80 randomly chosen fields following a 3 h treatment (**d**). NHFs were transfected with the indicated siRNAs. Where indicated, cells were pre-treated for 30 min with cyclosporin A (CsA). *P* values were calculated by one-way ANOVA and multiple-comparisons test. Error bars indicate s.e.m. **e**, COS-7 cells were transfected with either GFP-tagged IP3R3 or GFP-tagged IP3R3(Q-FR/A-AA). 16 h post-transfection, cells were serum-starved for 48 h, and then re-stimulated with serum for the indicated times. Cells were harvested, and whole-cell lysates (WCLs) were immunoblotted as indicated. The graph shows the quantification of IP3R3 levels from two independent experiments. **f**, COS-7 cells transfected with the indicated constructs were serum-starved for 20 h and then re-stimulated with serum for 4 h. WCLs were immunoblotted as indicated. **g**, COS-7 cells transfected with the indicated constructs were serum-starved for 20 h, re-stimulated for 4 h with or without MG132, and treated with ATP. Left, representative traces show concentrations of cytosolic Ca^2+^ measured with aequorin. Right, quantification of three independent experiments. *P* values were calculated by one-way ANOVA and multiple-comparisons test. Error bars indicate s.e.m. **h**, **i**, COS-7 cells transfected with the indicated constructs were serum-starved for 20 h, re-stimulated with serum for 4 h, and then treated with H_2_O_2_. Apoptosis shown in **h** was evaluated as in **b**, except that H_2_O_2_ treatment was for 5 h. Analysis of cytochrome *c* release shown in i was evaluated as in **d**. *P* values were calculated by unpaired *t-*test. Error bars indicate s.e.m. Unless otherwise noted, experiments were performed at least three times. For gel source data, see Supplementary Fig. 1.

**Figure 3:**
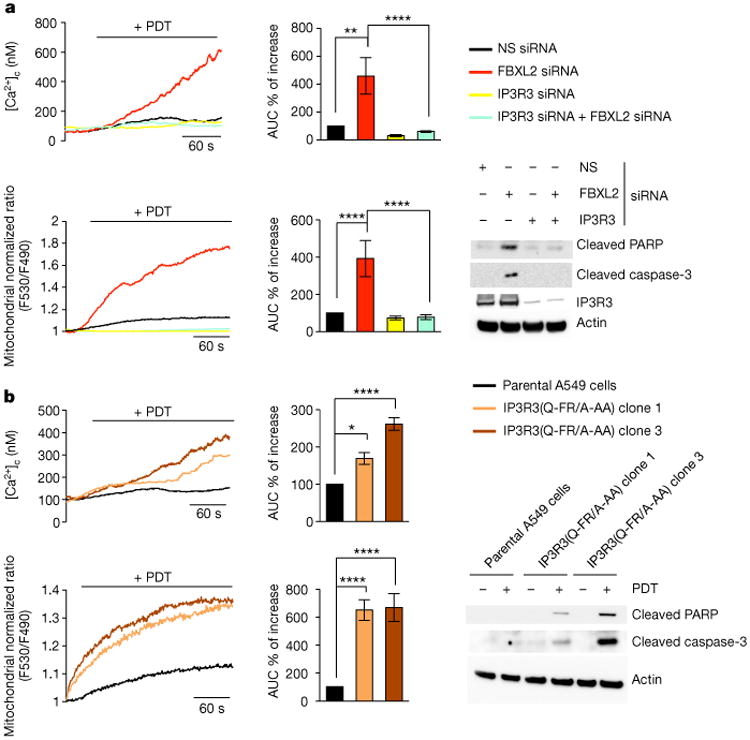
Failure to degrade IP3R3 results in sensitization to PDT-induced apoptosis. a, A549 cells were transfected with the indicated siRNAs and, after 48 h, treated with phthalocyanine, a photosensitizer used for PDT in patients with cancer. Top, cytosolic Ca^2+^ concentrations measured with Fura-2. Bottom (left and middle panels), mitochondrial Ca^2+^ mobilization in cells expressing the Ca^2+^-sensitive probe 4mtD3cpv. Left panels show representative traces, and panels on their right show quantifications of areas under the curve represented as percentage change compared to empty-vector-transfected cells, which were set as 100%. Bottom (right panel), immunoblots of a representative experiment. *P* values were calculated by one-way ANOVA and multiple-comparisons test. Error bars indicate s.e.m. b, Parental A459 and two IP3R3(Q-FR/A-AA) A549 knock-in clones (1 and 3) were processed and analysed as in a. Unless otherwise noted, experiments were performed at least three times. For gel source data, see Supplementary Fig. 1.

**Figure 4:**
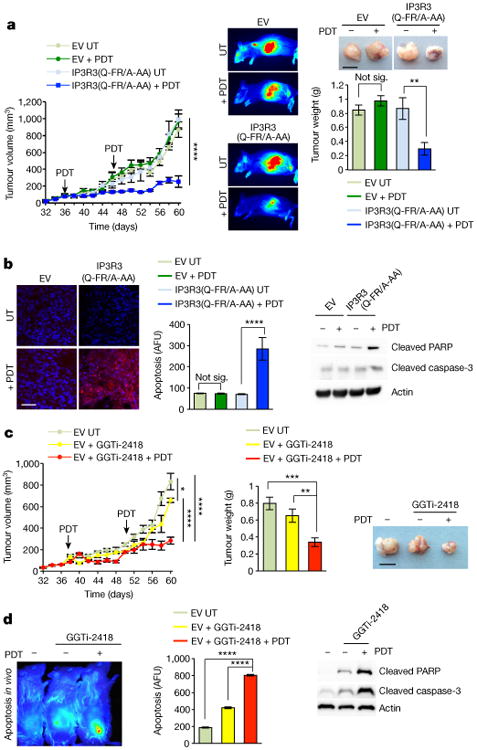
Both a non-degradable IP3R3 mutant and GGTi-2418 sensitize tumours to PDT **a**, NOD/SCID gamma mice were subcutaneously inoculated with 2 × 10^6^ A549 cells stably transfected with either an empty vector or IP3R3(Q-FR/A-AA). Implanted xenotransplanted mice, chosen randomly, were subjected to two rounds of PDT (see arrows) or left untreated (UT); *n* = 4–6 mice per group. Left panel, tumour growth kinetics at the indicated time points. Middle panel, representative tumours imaged with IRDye 2-DG at the end of the experiment. Top right panel, representative excised tumours imaged 60 days post-injection. Bottom right panel, quantification of tumour weights. *P* values for the tumour volume and weight at day 60 were calculated by one-way ANOVA and multiple-comparisons test. Error bars indicate s.e.m. Scale bar, 1 cm. **b**, Left panel, representative images of tumour sections from mice, which were injected with SR-FLIVO fluorescent probes to measure apoptosis. Middle panel, quantification of apoptosis. Right panel, corresponding immunoblots from homogenized tumours. AFU, arbitrary fluorescence units. *P* values were calculated by one-way ANOVA and multiple-comparisons test. Error bars indicate s.e.m. Scale bar, 50 |im. **c**, NOD/SCID gamma mice were subcutaneously injected with A549 cells. Implanted xenotransplanted mice, chosen randomly, were treated with GGTi-2418 alone or in combination with PDT; *n* = 5–6 mice per group. Left panel, tumour growth kinetics for the indicated time points. Middle panel, quantification of tumour weights. Right panel, representative excised tumours imaged 60 days post-injection. *P* values for the tumour volume and weight at day 60 were calculated by one-way ANOVA and multiple-comparisons test. Error bars indicate s.e.m. Scale bar, 1 cm. **d**, Left panel, PDT-induced apoptosis detected by NIR-FLIVO fluorescently labelled probes in the subcutaneous tumour masses of mice treated with or without GGTi-2418. Middle panel, quantification of apoptosis. Right panel, corresponding immunoblots from homogenized tumours. *P* values were calculated by one-way ANOVA and multiple-comparisons test. Error bars indicate s.e.m. Unless otherwise noted, experiments were performed at least three times. For gel source data, see Supplementary Fig. 1.
