# Supplementary material for: PTEN counteracts FBXL2 to promote IP3R3– and Ca2+–mediated apoptosis limiting tumour growth
Source: Nature. Author manuscript; Available in PMC 2017 Oct 4. (PMC5627969; doi:10.1038/nature22965)
Supplement: Supplemental 1 [file NIHMS906616-supplement-Supplemental_1.pdf]

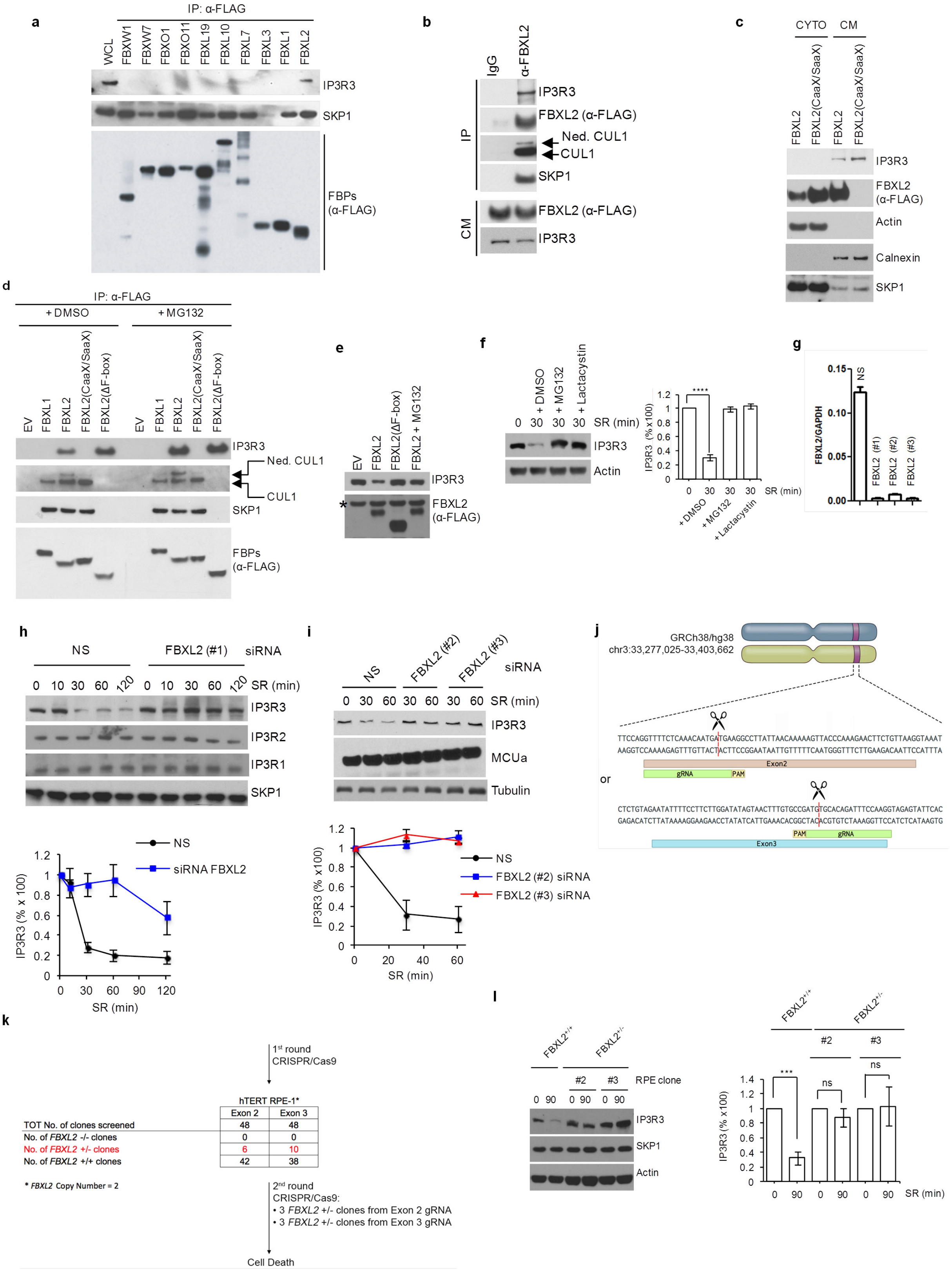

	Exon 2	Exon 3
TOT No. of clones screened	48	48
No. of *FBXL2* -/- clones	0	0
No. of *FBXL2* +/- clones	6	10
No. of *FBXL2* +/+ clones	42	38

Extended Data Figure 1 | See next page for caption.

© 2017 Macmillan Publishers Limited, part of Springer Nature. All rights reserved.

Extended Data Figure 1 | IP3R3 is targeted for proteasomal degradation by FBXL2. a, HEK293T cells were transfected with the indicated Flag-tagged F-box proteins (FBPs). 24 h post-transfection, cells were treated with MG132 for 3 h before harvesting for immunoprecipitation and immunoblotting as indicated. WCL, whole-cell lysate from untransfected cells. **b**, Flag-tagged FBXL2 was stably expressed in HeLa cells. After harvesting, cells were lysed, and cell membranes (CM) were isolated and immunoprecipitated with either a normal mouse IgG or an anti-Flag antibody. Subsequently, immunoprecipitates were analysed by immunoblotting as indicated. The results of these experiments ($n=2$) indicate that FBXL2 is incorporated into a complete SCF ligase that binds its substrate(s) at cellular membranes. **c**, Flag-tagged FBXL2 or Flag-tagged FBXL2(CaaX/SaaX) were transiently expressed in HeLa cells. After harvesting, cells were lysed and cytoplasmic (CYTO) and cell membrane (CM) fractions were isolated and analysed by immunoblotting as indicated. Actin and calnexin are used as markers for cytoplasmic and cell membrane fractions, respectively. This experiment was performed twice. **d**, HEK293T cells were transfected with either an empty vector (EV) or the indicated Flag-tagged proteins. 24 h post-transfection, where indicated, cells were treated with MG132 for 3 h before harvesting for immunoprecipitation and immunoblotting. Ned. CUL1, neddylated CUL1. This experiment shows that FBXL2, but not FBXL2(CaaX/SaaX), interacts with endogenous, neddylated CUL1. Since the covalent linkage of NEDD8 to CUL1 stimulates the ubiquitin ligase activity of SCFs and is promoted by the binding of the substrate to the F-box protein subunit, this result suggests that FBXL2 localization to cell membranes is required for substrate binding, which in turn stimulates CUL1 neddylation and SCF activation. Moreover, FBXL2(ΔF-box) bound more IP3R3 than wild-type FBXL2, and this difference could be abolished by treatment with MG132, supporting the hypothesis that FBXL2(ΔF-box) cannot mediate the degradation of IP3R3 since it does not form an active SCF complex. **e**, HEK293T cells were transfected with either an empty vector or the indicated Flag-tagged proteins. The experiment was performed in the presence or absence of MG132 as indicated. Whole-cell lysates were immunoblotted as indicated. The asterisk indicates a non-specific band. This experiment was performed twice. The fact that proteasome inhibitors prevented the decrease of IP3R3 levels upon re-addition of serum suggests that IP3R3 is degraded by the proteasome in response to mitogens, in agreement with previous studies reporting IP3Rs as substrates of the proteasome[26,27]. **f**, Normal, non-transformed, non-immortalized, human diploid fibroblasts (NHFs) (passage 2) were serum-starved for 72 h and then re-stimulated with serum (SR) for 30 min in the absence or presence of either MG132 (a proteasome inhibitor) or lactacystin (another proteasome inhibitor) as indicated. The graph on the right shows the quantification of IP3R3 levels from three independent experiments. *P* values were calculated by one-way ANOVA. Error bars indicate s.e.m. **g**, NHFs (passage 2) were transfected with either three different siRNAs targeting FBXL2 (each independently) or a non-silencing siRNA (NS). The graph shows FBXL2 mRNA levels analysed using real-time PCR in triplicate measurements. Error bars indicate s.e.m. The values represent the ratios between FBXL2 and GAPDH mRNAs. **h**, During a 72 h serum starvation, NHFs (passage 2 or 3) were transfected with either an siRNA targeting FBXL2 (#1) or a non-silencing siRNA (NS). Cells were subsequently stimulated with medium containing serum and harvested at the indicated times for immunoblotting. The graph shows the quantification of IP3R3 levels from three independent experiments. Error bars indicate s.e.m. **i**, During a 72 h serum starvation, NHFs (passage 3 or 4) were transfected with either siRNAs targeting FBXL2 (oligo #2 or #3) or a non-silencing siRNA (NS). Cells were subsequently stimulated with medium containing serum and harvested at the indicated time points for immunoblotting. The graph shows the quantification of IP3R3 levels from three independent experiments. Error bars indicate s.e.m. **j**, Schematic representation of the *FBXL2* genomic locus and gRNAs target location. Exon 2 and exon 3 refer to the human *FBXL2* gene in NC_000003.12 (GRCh38.p7 (Gene Bank ID: 3129728)). **k**, Schematic representation of *FBXL2* CRISPR–Cas9 mutagenesis outcomes. A first round of CRISPR–Cas9 gene editing yielded no homozygous *FBXL2*-knockout clones. A secondary round of CRISPR–Cas9 gene editing was carried out in three *FBXL2*$^{+/-}$ hTERT RPE-1 clones, which resulted in cell death, suggesting that *FBXL2* is required for cell fitness and it is not possible to generate *FBXL2*-knockout cells. Similar results were obtained in A549 cells (data not shown). **l**, *FBXL2*$^{+/+}$ and *FBXL2*$^{+/-}$ RPE-1-hTERT cells (clones 2 and 3) were serum-starved for 72 h and subsequently stimulated with medium containing serum for 90 min, after which cell extracts were immunoblotted for the indicated proteins. The graph shows the quantification of IP3R3 levels from three independent experiments. Unless otherwise noted, experiments were performed at least three times. For gel source data, see Supplementary Fig. 1.

© 2017 Macmillan Publishers Limited, part of Springer Nature. All rights reserved.

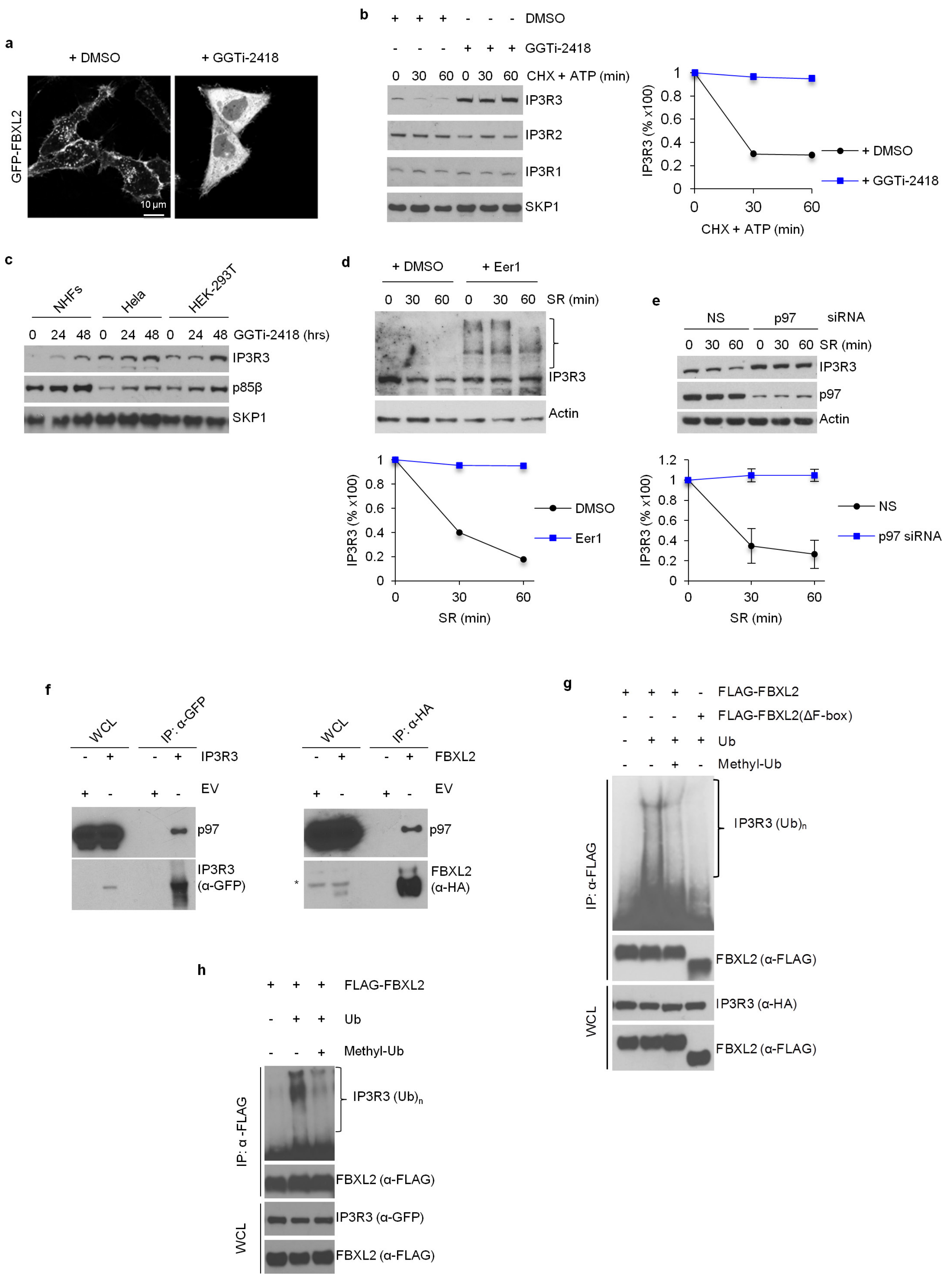

Extended Data Figure 2 | See next page for caption.

© 2017 Macmillan Publishers Limited, part of Springer Nature. All rights reserved.

Extended Data Figure 2 | The degradation of IP3R3 is dependent on FBXL2 localization to cell membranes and on the segregase activity of p97. **a**, HeLa cells were transfected with GFP-tagged FBXL2 and then treated with either DMSO or GGTI-2418 for 16 h. Live cell imaging was carried out with an LSM510 confocal microscope using a 63× objective. Scale bars, 10 μm. **b**, NHFs were incubated with GGTi-2418 for 30 h and then with cycloheximide (CHX) and ATP. Cells were subsequently harvested at the indicated times for immunoblotting. The graph shows the quantification of IP3R3 levels from two independent experiments. **c**, NHFs (passage 3), HeLa and HEK293T cells were incubated with GGTi-2418 for the indicated times. Cells were subsequently harvested for immunoblotting. This experiment was performed once. **d**, NHFs (passage 3) were serum-starved for 72 h, treated with either DMSO or Eer1, and then re-stimulated with serum (SR) for the indicated times. The graph shows the quantification of IP3R3 levels from two independent experiments. The bracket on the right marks a ladder of bands which, presumably, are ubiquitinated species of IP3R3 that are not degraded when p97 is inhibited. **e**, During a 72 h serum starvation, NHFs (passage 3 and 4) were transfected with either an siRNA targeting p97 or a non-silencing siRNA (NS). Cells were subsequently stimulated with medium containing serum and harvested at the indicated time points for immunoblotting. The graph shows the quantification of IP3R3 levels from three independent experiments. Error bars indicate s.e.m. These results, together with those shown in **d**, are in agreement with the findings that IP3Rs are ubiquitinated while they are membrane-associated, and those showing that p97 promotes the degradation of IP3Rs[27,28]. Thus, we propose that, after its FBXL2-mediated ubiquitination, IP3R3 is extracted from cell membranes by the segregase activity of p97 to be degraded by the proteasome. **f**, HEK293T cells were transfected with either an empty vector (EV) or the indicated GFP-tagged and HA-tagged proteins. 24 h post-transfection, cells were treated with MG132 for 3 h before harvesting for immunoprecipitation and immunoblotting as indicated. This experiment was performed twice. **g**, **h**, HEK293T cells were transfected with Flag-tagged FBXL2 and the indicated versions of tagged IP3R3. After immunopurification with an anti-Flag resin, *in vitro* ubiquitination of IP3R3 was performed in the presence of UAE1, Ubch3, Ubch5 and ubiquitin (Ub). Where indicated, an excess of methylated ubiquitin (methyl-Ub), which blocks chain extension, was added to the *in vitro* reactions. The presence of methyl-Ub resulted in the disappearance of the highest molecular weight forms of IP3R3, demonstrating that the high molecular weight forms of IP3R3 are indeed polyubiquitinated species of the protein. Samples were analysed by immunoblotting with the indicated antibodies. The bracket on the right marks a ladder of bands corresponding to ubiquitinated IP3R3. Immunoblots of whole-cell lysates (WCL) are shown at the bottom. Unless otherwise noted, experiments were performed at least three times. For gel source data, see Supplementary Fig. 1.

© 2017 Macmillan Publishers Limited, part of Springer Nature. All rights reserved.

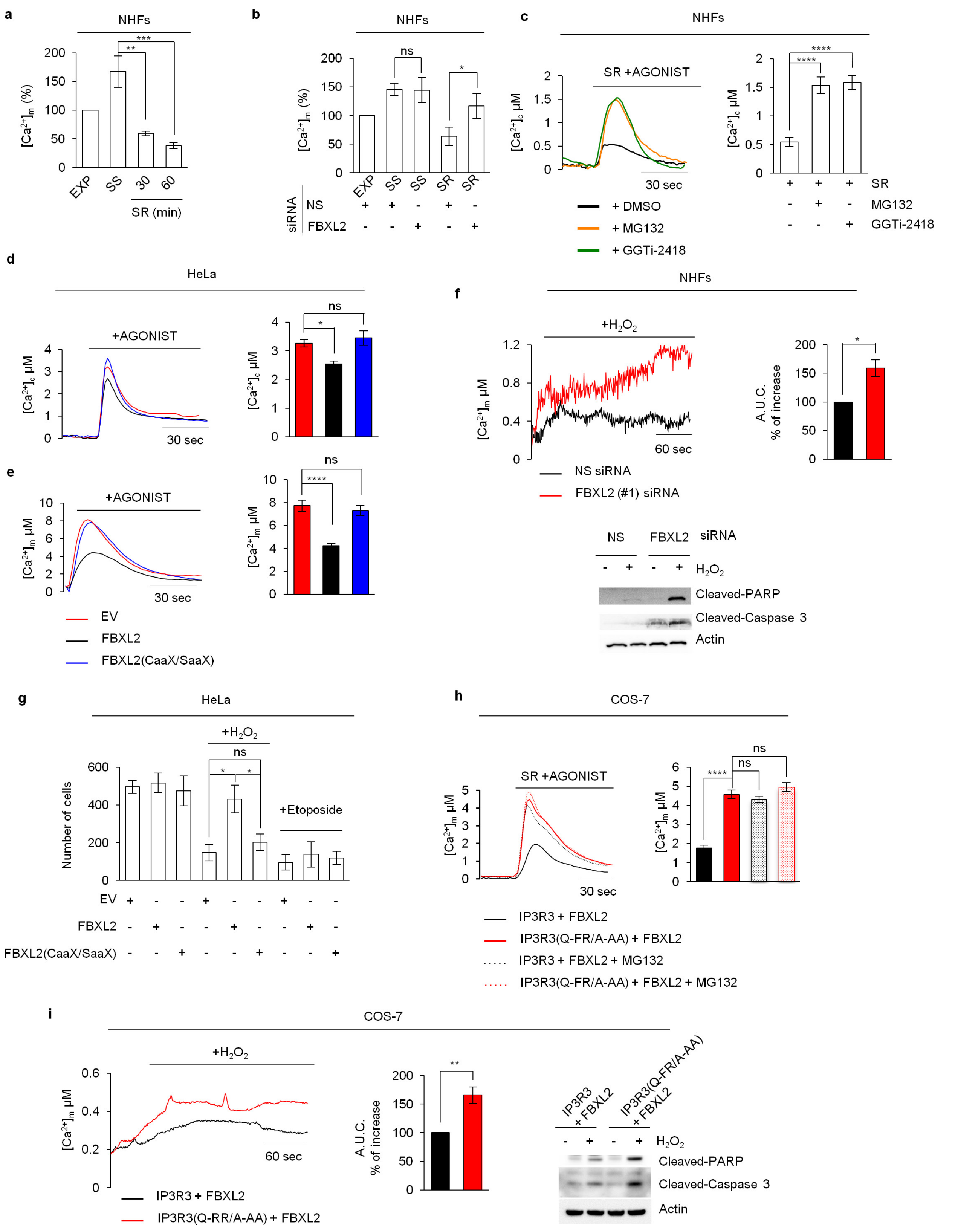

Extended Data Figure 3 | See next page for caption.

© 2017 Macmillan Publishers Limited, part of Springer Nature. All rights reserved.

Extended Data Figure 3 | FBXL2 controls Ca^{2+} mobilization and Ca^{2+}-mediated apoptosis. **a**, Concentrations of mitochondrial Ca^{2+} were measured with mitochondria-targeted aequorin in response to agonist stimulation (ATP, a purinergic GPCR (G-protein-coupled receptor) agonist) in exponentially growing (EXP), serum-starved (SS), and serum re-stimulated (SR) NHFs (passage 4 or 5). Quantification of three independent experiments is shown and represented as percentage change compared to EXP cells, which were set as 100%. *P* values were calculated by one-way ANOVA and multiple-comparisons test. Error bars indicate s.e.m. **b**, Concentrations of mitochondrial Ca^{2+} were measured as in **a** in exponentially growing (EXP), serum-starved (SS), and serum re-stimulated for one hour (SR) NHFs (passage 5) transfected with either an siRNA targeting FBXL2 (#1) or a non-silencing siRNA (NS). Quantifications and *P* value analyses was performed as in **a**. **c**, Concentrations of cytosolic Ca^{2+} were measured with aequorin in response to agonist stimulation (ATP) in NHFs (passage 2) re-stimulated with serum for one hour (SR) in the absence or presence of MG132 or GGTi2418. On the left, representative traces. On the right, quantification of three independent experiments. *P* values were calculated by a one-way ANOVA and multiple-comparisons test. Error bars indicate s.e.m. **d**, **e**, HeLa cells were transfected with either an empty vector (EV), FBXL2 or FBXL2(CaaX/SaaX). Concentrations of cytosolic (**d**) and mitochondrial (**e**) Ca^{2+} were measured with the appropriate aequorin in response to agonist stimulation (ATP). On the left, representative traces. On the right, quantifications of three independent experiments. *P* values were calculated by one-way ANOVA and multiple-comparisons test. Error bars indicate s.e.m. **f**, Concentrations of mitochondrial Ca^{2+} were measured with mitochondrial targeted aequorin upon treatment with H_2O_2 in NHFs (passage 2) transfected with either an siRNA targeting FBXL2 (#1) or a non-silencing siRNA (NS). On the left, representative traces. On the right, quantifications of areas under the curve (AUC) are represented as percentage increase compared to NS-transfected cells, which were set as 100%. *P* values were calculated by unpaired *t*-test. Error bars indicate s.e.m. At the bottom, immunoblots of cell lysates of a representative experiment upon treatment with H_2O_2 for 3 h. **g**, HeLa cells transfected with either an empty vector (EV), FBXL2 or FBXL2(CaaX/SaaX) were treated with either H_2O_2 (for 5 h) or etoposide (for 5 h). Induction of cell death was evaluated using automated nuclei count analysis of twenty randomly chosen fields. *P* values were calculated by one-way ANOVA and multiple-comparisons test. Error bars indicate s.e.m. **h**, COS-7 cells expressing either IP3R3 or IP3R3(Q-FR/A-AA) in combination with Flag-tagged FBXL2 were serum-starved for 20 h, re-stimulated with serum (SR) for 4 h with or without MG132, as indicated, and treated with ATP. Left, representative traces showing concentrations of mitochondrial Ca^{2+} measured with mitochondrial targeted aequorin. Right, quantification of three independent experiments. *P* values were calculated by one-way ANOVA and multiple-comparisons test. Error bars indicate s.e.m. **i**, Concentrations of mitochondrial Ca^{2+} were measured with mitochondria-targeted aequorin upon treatment with H_2O_2 in COS-7 cells expressing either IP3R3 or IP3R3(Q-FR/A-AA) in combination with Flag-tagged FBXL2. Left, representative traces. Middle, quantifications of areas under the curve represented as percentage increase compared to NS-transfected cells, which were set as 100%. *P* values were calculated using an unpaired *t*-test. Error bars indicate s.e.m. Right, immunoblots of cell lysates of a representative experiment upon treatment with H_2O_2 for 3 h. Unless otherwise noted, experiments were performed at least three times. For gel source data, see Supplementary Fig. 1.

© 2017 Macmillan Publishers Limited, part of Springer Nature. All rights reserved.

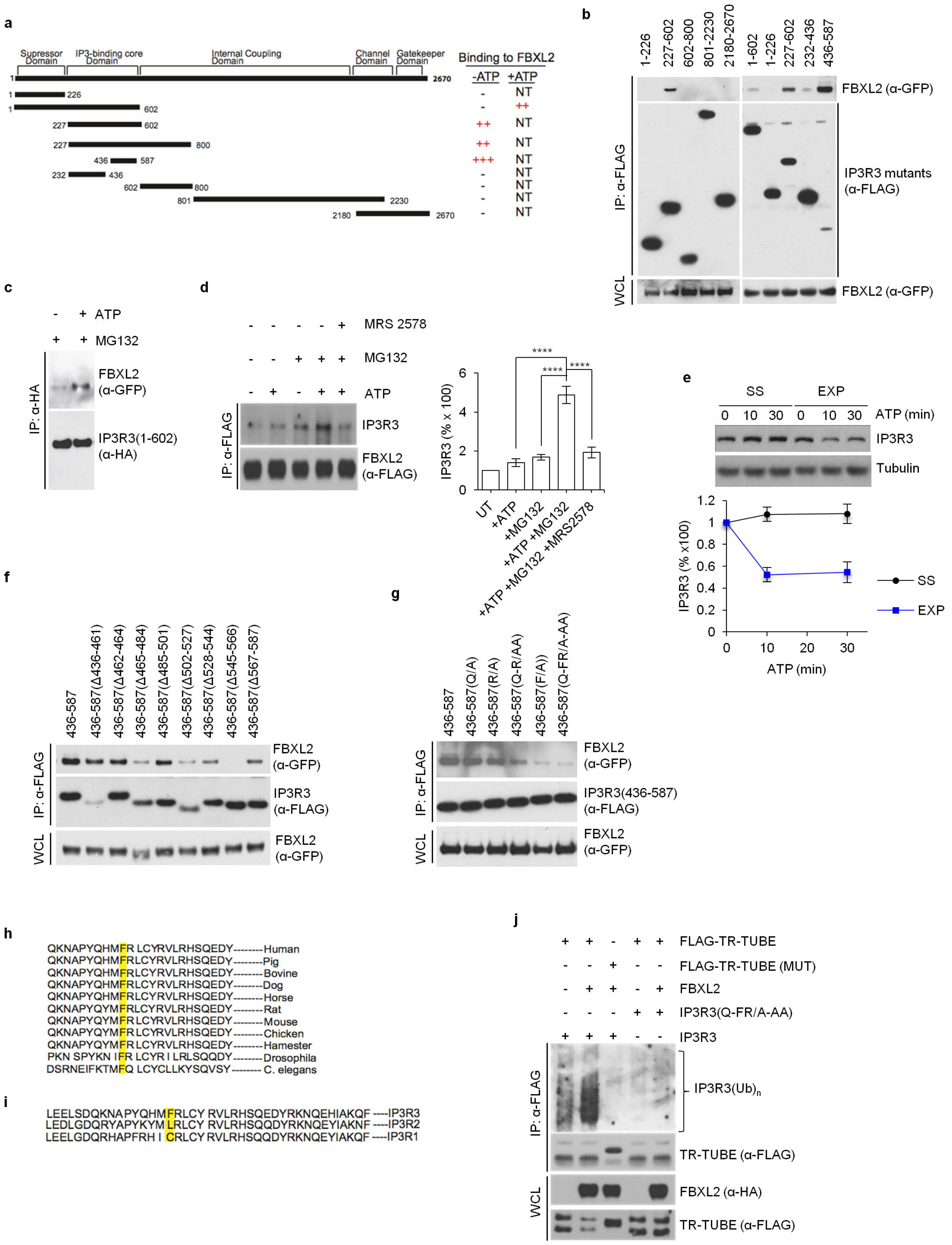

Extended Data Figure 4 | See next page for caption.

© 2017 Macmillan Publishers Limited, part of Springer Nature. All rights reserved.

Extended Data Figure 4 | Mapping of the FBXL2-binding domain in IP3R3, and evidence that the N-terminal suppressor domain inhibits the IP3R3–FBXL2 interaction. **a**, Schematic representation of IP3R3 mutants. Binding of IP3R3 to FBXL2 is indicated with the symbol (+). **b**, HEK293T cells were transfected with GFP-tagged FBXL2 and the indicated Flag-tagged IP3R3 truncated mutants. Whole-cell lysates (WCL) were immunoprecipitated (IP) with anti-Flag resin and proteins were immunoblotted as indicated. This experiment was performed twice. **c**, HEK293T cells were transfected with GFP-tagged FBXL2 and HA-tagged In IP3R3(1–602) constructs. Sixteen hours after transfection, cells were incubated with MG132 for 3 h before stimulation with ATP for 30 min. Whole-cell lysates were immunoprecipitated (IP) with an anti-HA resin and proteins were immunoblotted as indicated. This experiment was performed twice. **d**, HeLa cells stably transfected with Flag-tagged FBXL2 under the control of a doxycycline-inducible promoter were treated with doxycycline ($0.4\,\mu g\,ml^{-1}$) for 16 h and incubated with or without MG132 (during the last 3 h), in the presence or absence of MRS 2578, an antagonist of P2Y6 receptor (during the last 90 min), as indicated. Cells were subsequently treated with or without ATP for 30 min. Whole-cell lysates were immunoprecipitated (IP) with an anti-Flag resin and proteins were immunoblotted as indicated. Right panel shows quantifications of IP3R3 levels compared to untreated cells (UT), which were set as 100%. *P* values were calculated by one-way ANOVA and multiple-comparisons test. Error bars indicate s.e.m. The fact (shown in **b**) that fragments encoding IP3R3(436–587) and IP3R3(227–602) interact with FBXL2 better than the IP3R3(1–602) fragment suggests that the N terminus of IP3R3 inhibits the interaction between FBXL2 and IP3R3. It has been shown that removal of the N-terminal suppressor domain (amino acids 1–226) increases the binding of IP3 to the IP3 binding core domain (amino acids 227–579), and IP3 binding evokes conformational changes that open the suppressor domain[29–32]. These conformational changes may allow FBXL2 to access the degron of IP3R3 (that is, the amino acid region required for binding to FBXL2). Thus, in agreement with previous data indicating that IP3 causes the ubiquitination and downregulation of IP3Rs (ref. 27), our results suggest that IP3 promotes the binding of FBXL2 to IP3R3 and that FBXL2 preferentially binds IP3R3 in its open conformation (upon IP3 binding). Accordingly, treatment of cells with ATP, which induces IP3 production and repositioning of the N-terminal suppressor domain, increased the binding between FBXL2 and IP3R3, particularly if proteasomal degradation was inhibited by MG132. This suggests that once IP3 (produced after ATP stimulation) unmasks the IP3R3 degron, FBXL2 binds IP3R3 and this interaction is preserved when FBXL2-mediated degradation of IP3R3 is inhibited. So, ATP and MG132 appear to synergize with each other in promoting and preserving the FBXL2–IP3R3 interaction, respectively. **e**, Serum-starved (SS) and exponentially (EXP) growing NHFs (passage 2 and 3) were treated with or without ATP as indicated. Cells were subsequently harvested at the indicated time points for immunoblotting. The graph shows the quantification of IP3R3 levels. Error bars indicate s.e.m. **f**, HEK293T cells were transfected with GFP-tagged FBXL2 and the indicated Flag-tagged IP3R3 deletion mutants (in the context of the 436–587 domain of IP3R3). Whole-cell lysates (WCL) were immunoprecipitated (IP) with anti-Flag resin, and proteins were immunoblotted as indicated. This experiment was performed twice. **g**, The experiment was performed twice as in **f**, except that different IP3R3 mutants were used. **h**, Alignment of the amino acid regions containing the FBXL2-binding motif in IP3R3 orthologues. **i**, Alignment of the amino acid regions containing the FBXL2-binding motif in human IP3R3 with the corresponding region in IP3R1 and IP3R2. **j**, HEK293T cells were transfected with the indicated constructs. Whole-cell lysates (WCL) were immunoprecipitated (IP) with anti-Flag resin and immunoblotted as indicated. The bracket on the right marks a ladder of bands corresponding to polyubiquitinated IP3R3. This experiment was performed twice. Unless otherwise noted, experiments were performed at least three times. For gel source data, see Supplementary Fig. 1.

© 2017 Macmillan Publishers Limited, part of Springer Nature. All rights reserved.

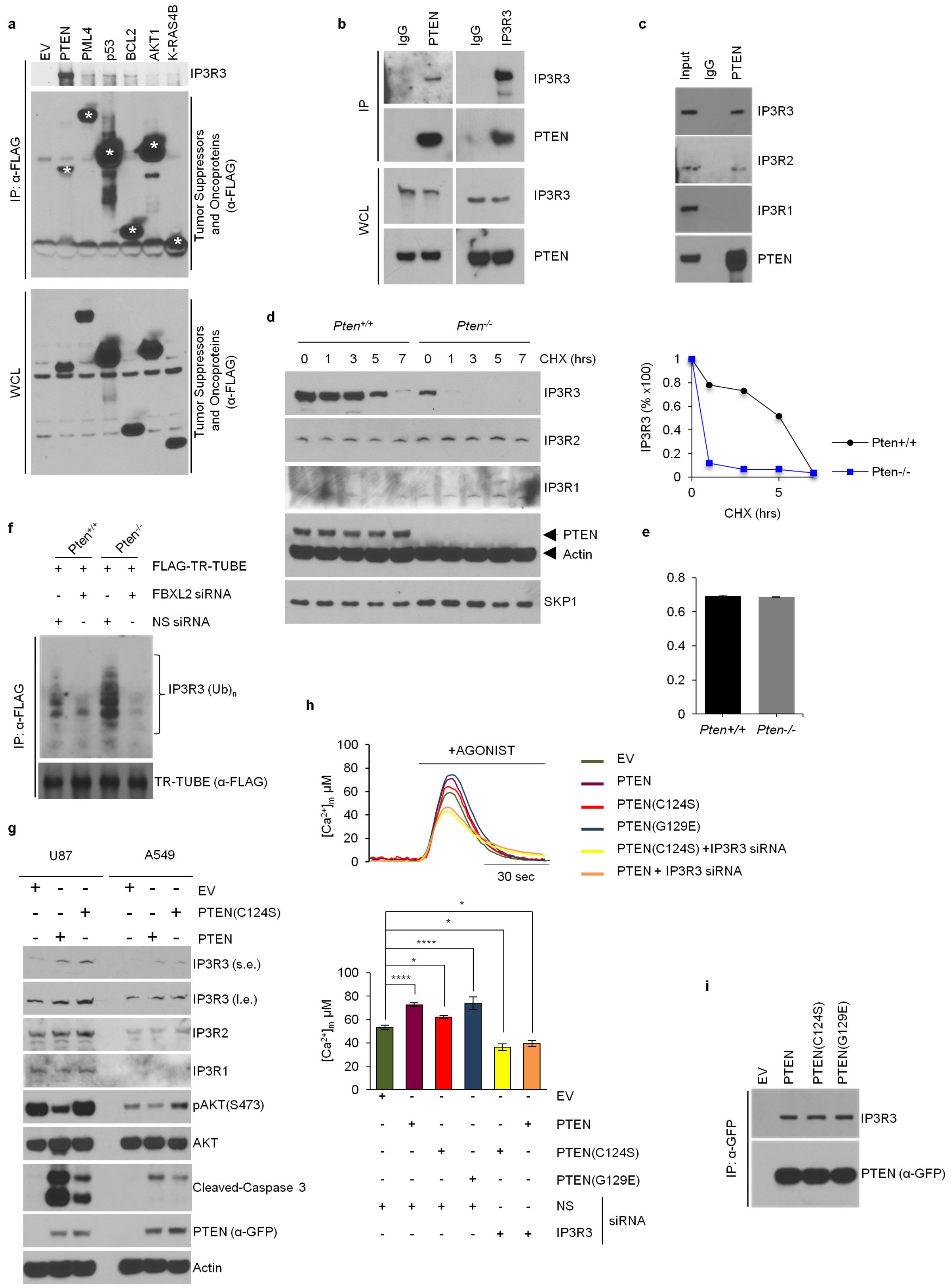

Extended Data Figure 5 | See next page for caption.

© 2017 Macmillan Publishers Limited, part of Springer Nature. All rights reserved.

Extended Data Figure 5 | IP3R3 degradation is prevented by PTEN, independently of its phosphatase activity. a, HEK293T cells were transfected with the indicated Flag-tagged constructs encoding tumour suppressors and oncoproteins with established roles in Ca^{2+} homeostasis and that are known to localize to the endoplasmic reticulum–mitochondria interface (AKT1, BCL2, p53, PML4, PTEN and KRAS4B). Of those, AKT1, BCL2, PML4 and PTEN are known IP3R3 interactors[33–39]. Twenty-four hours post-transfection, cells were harvested for anti-Flag immunoprecipitations and immunoblotting as indicated. Asterisks denote Flag-tagged proteins. This experiment was performed twice. **b**, **c**, Whole-cell lysates (WCL) from HEK293T cells were immunoprecipitated with the indicated antibodies and immunoblotted as indicated. This experiment was performed twice. **d**, *Pten*$^{+/+}$ and *Pten*$^{-/-}$ MEFs were incubated with cycloheximide (CHX) for the indicated times. Cells were subsequently harvested for immunoblotting as indicated. The graph on the right shows the quantification of IP3R3 levels from two independent experiments. **e**, FBXL2 mRNA levels in *Pten*$^{+/+}$ and *Pten*$^{-/-}$ MEFs analysed using real-time PCR in triplicate measurements (± s.e.m.). The values represent the ratios between FBXL2 and GAPDH mRNAs. This experiment was performed twice. **f**, *Pten*$^{+/+}$ and *Pten*$^{-/-}$ MEFs were transfected for 48 h with either an siRNA targeting FBXL2 (#1) or a non-silencing siRNA (NS). Subsequently, MEFs were transfected with Flag-TR-TUBE cDNA. Whole-cell lysates (WCL) were immunoprecipitated (IP) with anti-Flag resin and immunoblotted as indicated. The bracket on the right marks a ladder of bands corresponding to polyubiquitinated IP3R3. This experiment was performed twice. **g**, U87 and A549 cells were transfected with either GFP-tagged PTEN, GFP-tagged PTEN(C124S), or an empty vector (EV) as indicated. Cells were subsequently harvested and whole-cell lysates were immunoblotted as indicated. Long (l.e.) and short (s.e.) exposures are shown for IP3R3. **h**, *Pten*$^{-/-}$ MEFs were processed as in (Fig. 2c). Concentrations of mitochondrial Ca^{2+} were measured with mitochondria-targeted aequorin in response to agonist stimulation (ATP). Top, representative traces. Bottom, quantification of three independent experiments. *P* values were calculated by one-way ANOVA and multiple comparisons test. Error bars indicate s.e.m. **i**, HEK293T cells were transfected with either GFP-tagged wild-type PTEN or the indicated GFP-tagged cancer-associated PTEN mutants. Whole-cell lysates (WCL) were immunoprecipitated (IP) with anti-GFP resin and proteins were immunoblotted as indicated. This experiment was performed twice. Unless otherwise noted, experiments were performed at least three times. For gel source data, see Supplementary Fig. 1.

© 2017 Macmillan Publishers Limited, part of Springer Nature. All rights reserved.

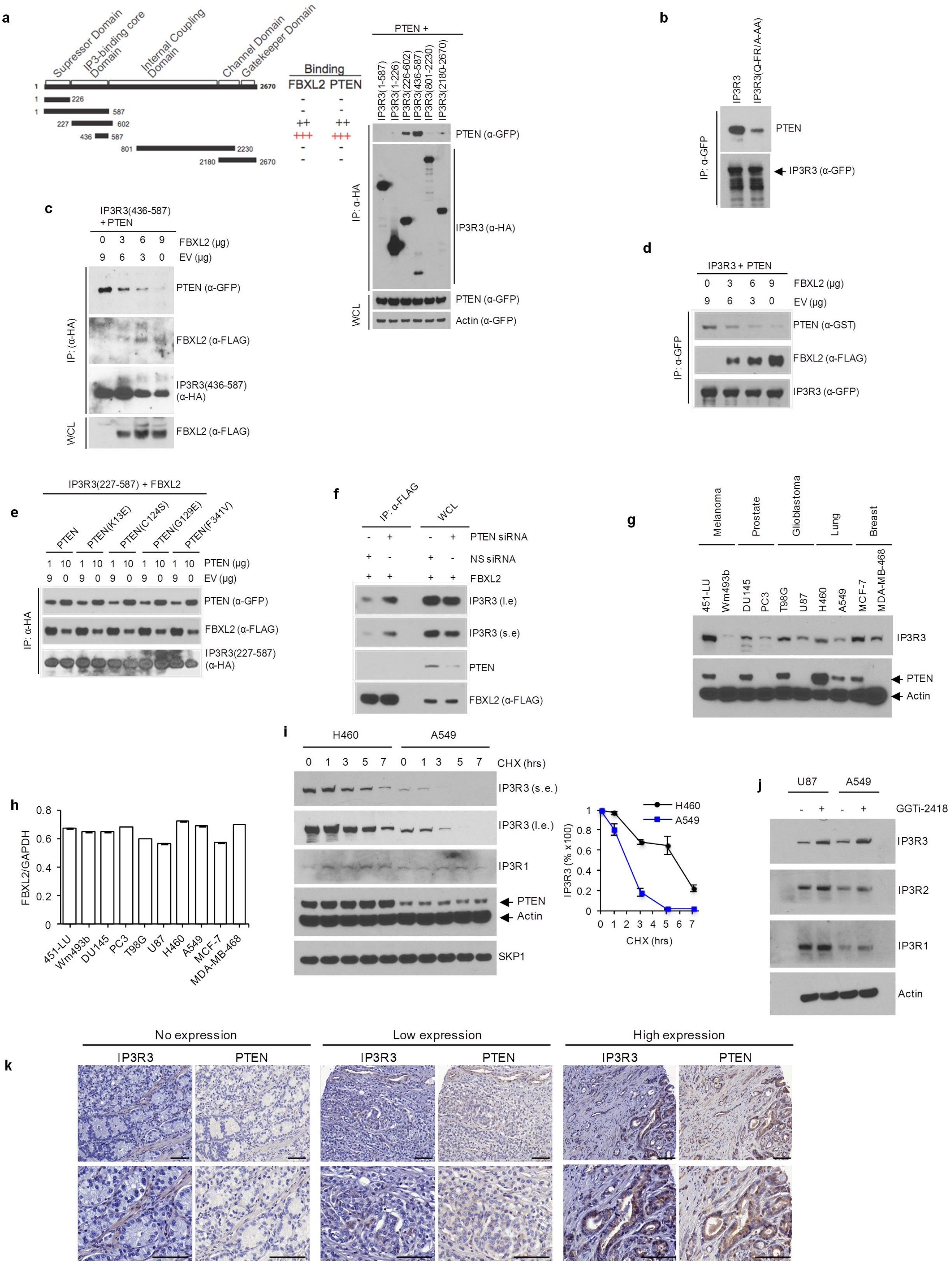

Extended Data Figure 6 | See next page for caption.

© 2017 Macmillan Publishers Limited, part of Springer Nature. All rights reserved.

Extended Data Figure 6 | PTEN competes with FBXL2 for the binding to IP3R3, and the levels of IP3R3 and PTEN directly correlate in cancer cell lines and human prostate tumours. a, Left, schematic representation of IP3R3 mutants used for binding site mapping. Binding of IP3R3 to FBXL2 and PTEN is indicated with the symbol (+). Right, HEK293T cells were transfected with GFP-tagged PTEN and the indicated HA-tagged IP3R3 truncated mutants. Whole-cell lysates (WCL) were immunoprecipitated (IP) with anti-HA resin and immunocomplexes were probed with antibodies to the indicated proteins. This experiment was performed twice. **b**, HEK293T cells were transfected with GFP-tagged IP3R3 or IP3R3(Q-FR/A-AA). Whole-cell lysates were immunoprecipitated (IP) with an anti-GFP resin and proteins were immunoblotted as indicated. **c**, HEK293T cells were co-transfected, as indicated, with GFP-tagged PTEN, HA-IP3R3(436–587), and increasing amounts of Flag-tagged FBXL2. Whole-cell lysates (WCL) were immunoprecipitated (IP) with anti-HA resin and proteins were immunoblotted as indicated. This experiment was performed twice. **d**, HEK293T cells were co-transfected, as indicated, with GST-tagged PTEN, GFP-tagged IP3R3, and increasing amounts of Flag-tagged FBXL2. Whole-cell lysates were immunoprecipitated (IP) with anti-GFP resin and proteins were immunoblotted as indicated. This experiment was performed twice. **e**, HEK293T cells were co-transfected, as indicated, with Flag-tagged FBXL2, HA-tagged IP3R3(436–587), and increasing amounts of either GFP-tagged PTEN or the indicated GFP-tagged cancer-associated PTEN mutants. Whole-cell lysates were immunoprecipitated (IP) with anti-HA resin and proteins were immunoblotted as indicated. This experiment was performed twice. **f**, HEK293T cells were transfected with either an siRNA targeting PTEN or a non-silencing siRNA (NS), as indicated. After 48 h, cells were transfected with Flag-tagged FBXL2. Sixteen hours after the second transfection, whole-cell lysates were immunoprecipitated (IP) with anti-Flag resin. Immunocomplexes and WCLs were immunoblotted as indicated. **g**, Whole-cell lysates from the indicated cancer cell lines were immunoblotted as indicated. This experiment was performed twice. **h**, FBXL2 mRNA levels in the indicated cell lines analysed using real-time PCR in triplicate measurements (± s.e.m.). The values represent the ratios between FBXL2 and GAPDH mRNAs. This experiment was performed once. **i**, H460 and A549 cells were incubated with cycloheximide (CHX) for the indicated times. Cells were subsequently harvested and processed for immunoblotting as indicated. Long (l.e.) and short (s.e.) exposures are shown for IP3R3. The graph shows the quantification of IP3R3 levels from three independent experiments. Error bars indicate s.e.m. **j**, U87 and A549 cells were treated with GGTi-2418 for 16 h where indicated. Cells were subsequently harvested and whole-cell lysates were immunoblotted as indicated. This experiment was performed twice. **k**, Representative immunohistochemistry staining images of human prostate tumour specimens with no, low or high levels of PTEN protein. Levels of IP3R3 in consecutive tissue slides are shown. Scale bars correspond to 50 μm. Unless otherwise noted, experiments were performed at least three times. For gel source data, see Supplementary Fig. 1.

© 2017 Macmillan Publishers Limited, part of Springer Nature. All rights reserved.

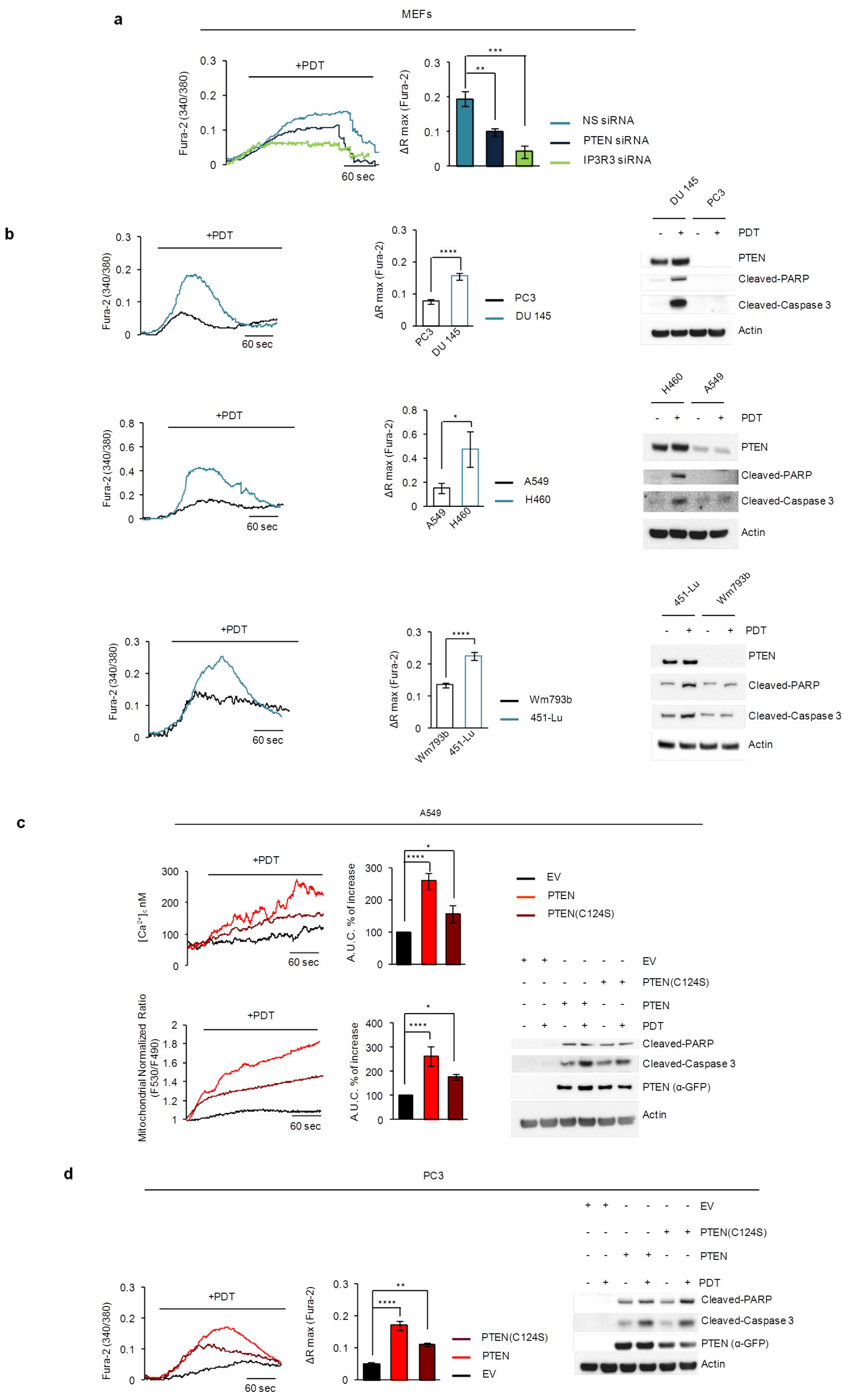

Extended Data Figure 7 | See next page for caption.

© 2017 Macmillan Publishers Limited, part of Springer Nature. All rights reserved.

Extended Data Figure 7 | Both wild-type PTEN and a phosphatase dead PTEN mutant sensitize cells to photodynamic therapy. a, MEFs were transfected for 48 h with either a non-silencing siRNA (NS) or siRNAs targeting PTEN or IP3R3 as indicated. Cells were loaded with Fura-2 dye for Ca^{2+} mobilization analysis upon treatment with PDT. Representative traces (left panel) show cytosolic calcium mobilization. Quantifications are shown in the right panel. *P* values were calculated by one-way ANOVA and multiple-comparisons test. ΔRmax, maximum variation in peak values of 340/380 ratiometric analysis. Error bars indicate s.e.m. **b**, Matched-pair cell lines expressing wild-type PTEN or displaying low or no PTEN expression (that is, DU145 and PC3 (prostate cancer cell lines); H460 and A549 (lung cancer cell lines); 451-LU and Wm493b (melanoma cell lines), respectively) were loaded with Fura-2 dye for Ca^{2+} mobilization analysis upon PDT treatment. Representative traces (left panels) show cytosolic calcium mobilization. Bar graphs (middle panels) show the quantification of three independent experiments. Right panels show corresponding whole cell extracts immunoblotted as indicated. *P* values were calculated by unpaired *t*-test. Error bars indicate s.e.m. **c**, A549 cells were transiently transfected with either GFP-tagged PTEN, GFP-tagged PTEN(C124S), or an empty vector (EV) as indicated. Cells were treated with phthalocyanine, a photosensitizer used for PDT in patients with cancer. Top panels show cytosolic Ca^{2+} concentrations measured with Fura-2. Bottom (left and middle panels), mitochondrial Ca^{2+} mobilization in cells expressing the Ca^{2+} sensitive probe 4mtD3cpv. Left panels show representative traces, and panels on their right show quantifications of areas under the curve represented as percentage increase compared to empty-vector-transfected cells, which were set as 100%. Right bottom panel shows immunoblots of cell lysates of a representative experiment. *P* values were calculated by one-way ANOVA and multiple-comparisons test. Error bars indicate s.e.m. **d**, PC3 cells were transiently transfected with either GFP-tagged PTEN, GFP-tagged PTEN(C124S), or an empty vector (EV) as indicated. Cells were loaded with Fura-2 dye for Ca^{2+} mobilization analysis upon PDT treatment. Representative traces show cytosolic calcium mobilization. Bar graphs show the quantification of three independent experiments. Right panel shows immunoblots of cell lysates of a representative experiment. *P* values were calculated by one-way ANOVA and multiple-comparisons test. Error bars indicate s.e.m. Unless otherwise noted, experiments were performed at least three times. For gel source data, see Supplementary Fig. 1.

© 2017 Macmillan Publishers Limited, part of Springer Nature. All rights reserved.

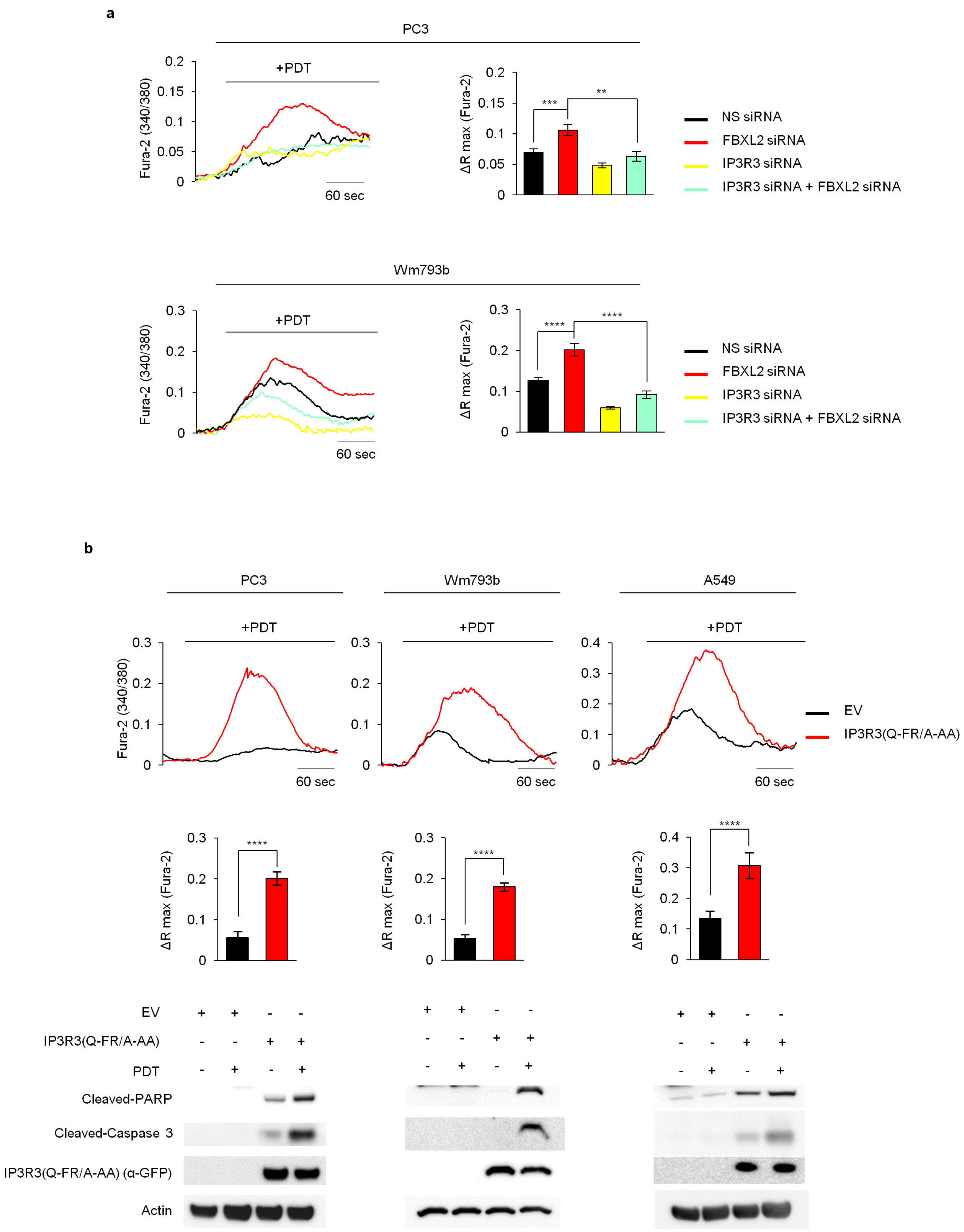

Extended Data Figure 8 | See next page for caption.

© 2017 Macmillan Publishers Limited, part of Springer Nature. All rights reserved.

Extended Data Figure 8 | Stabilization of IP3R3 sensitizes cells to photodynamic therapy. **a**, PC3 cells (top panels) and Wm493b cells (bottom panels) were transfected with a non-silencing siRNA (NS) or siRNAs targeting either FBXL2, IP3R3 or both FBXL2 and IP3R3. Cells were loaded with Fura-2 dye for Ca^{2+} mobilization analysis upon PDT treatment. Representative traces show cytosolic calcium mobilization. Bar graphs show the quantification of three independent experiments. ΔRmax, maximum variation in peak values of 340/380 ratiometric analysis. *P* values were calculated by one-way ANOVA and multiple-comparisons test. Error bars indicate s.e.m. **b**, PC3 cells (left panels), Wm493b cells (middle panels) and A549 cells (right panels) expressing either GFP-tagged IP3R3(Q-FR/A-AA) or an empty vector (EV) were loaded with Fura-2 dye for Ca^{2+} mobilization analysis upon PDT treatment. Representative traces show cytosolic calcium mobilization. Bar graphs show the quantification of three independent experiments. Statistical analysis was performed with unpaired *t*-tests. Error bars indicate s.e.m. Bottom panels show corresponding immunoblots of cell lysates of representative experiments. Unless otherwise noted, experiments were performed at least three times. For gel source data, see Supplementary Fig. 1.

© 2017 Macmillan Publishers Limited, part of Springer Nature. All rights reserved.

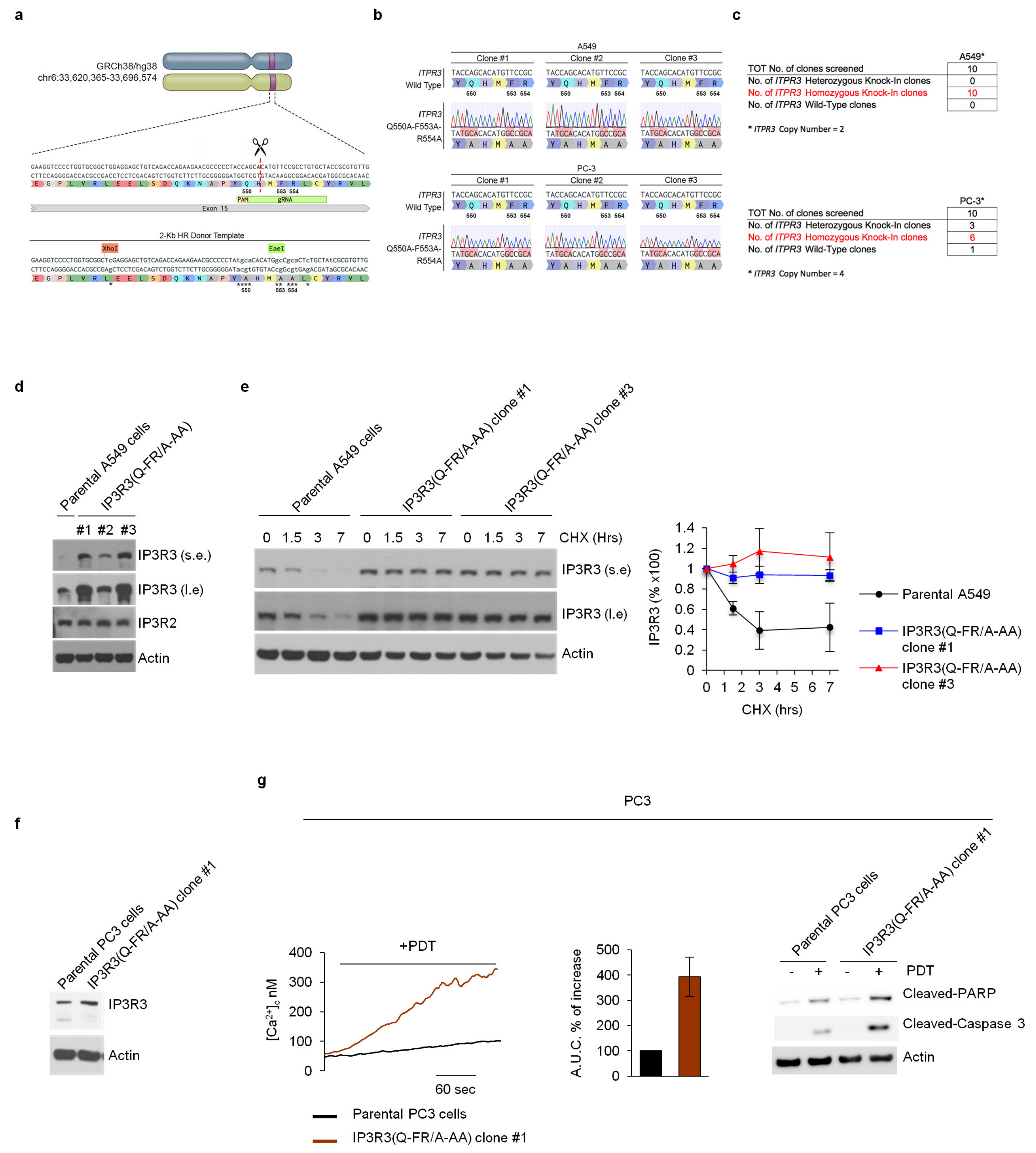

Extended Data Figure 9 | A non-degradable IP3R3 mutant sensitizes tumour cells to photodynamic therapy. a, Schematic representation of the *ITPR3* genomic locus and gRNA target location. Exon 15 refers to human *ITPR3* gene (GRCh38.p7 (Gene Bank ID: 31297280)). Silent mutations are shown as lower case letters and indicated by asterisks. **b**, Wild-type genomic DNA template and knock-in mutant sequences identified by TOPO-TA cloning of *ITPR3* PCR from three independent A549 and PC3 clones are depicted. **c**, Schematic representation of the *ITPR3* CRISPR–Cas9 mutagenesis outcomes for A549 and PC3 cells. **d**, Whole-cell lysates from A549 parental cells and three independent IP3R3(Q-FR/A-AA) knock-in clones were immunobloted as indicated. **e**, A549 parental cells and IP3R3(Q-FR/A-AA) knock-in cells (clones 1 and 3) were incubated with cycloheximide (CHX) for the indicated times. Cells were subsequently harvested for immunoblotting as indicated. The graph shows the quantification of IP3R3 levels from three independent experiments. Error bars indicate s.e.m. **f**, Whole-cell lysates from PC3 parental cells and an IP3R3(Q-FR/A-AA) knock-in clone (#1) were immunoblotted as indicated. **g**, PC3 parental cells and the IP3R3(Q-FR/A-AA) knock-in PC3 clone 1 were treated with phthalocyanine, a photosensitizer used for PDT in patients with cancer. Left panel, cytosolic Ca^{2+} concentrations measured with Fura-2. Middle panel, quantifications of areas under the curve represented as percentage increase compared to empty-vector-transfected cells, which were set as 100%. Right panel, immunoblots of cell lysates from a representative experiment. The *P* value was calculated by unpaired *t*-test. Error bars indicate s.e.m. Unless otherwise noted, experiments were performed at least three times. For gel source data, see Supplementary Fig. 1.

© 2017 Macmillan Publishers Limited, part of Springer Nature. All rights reserved.

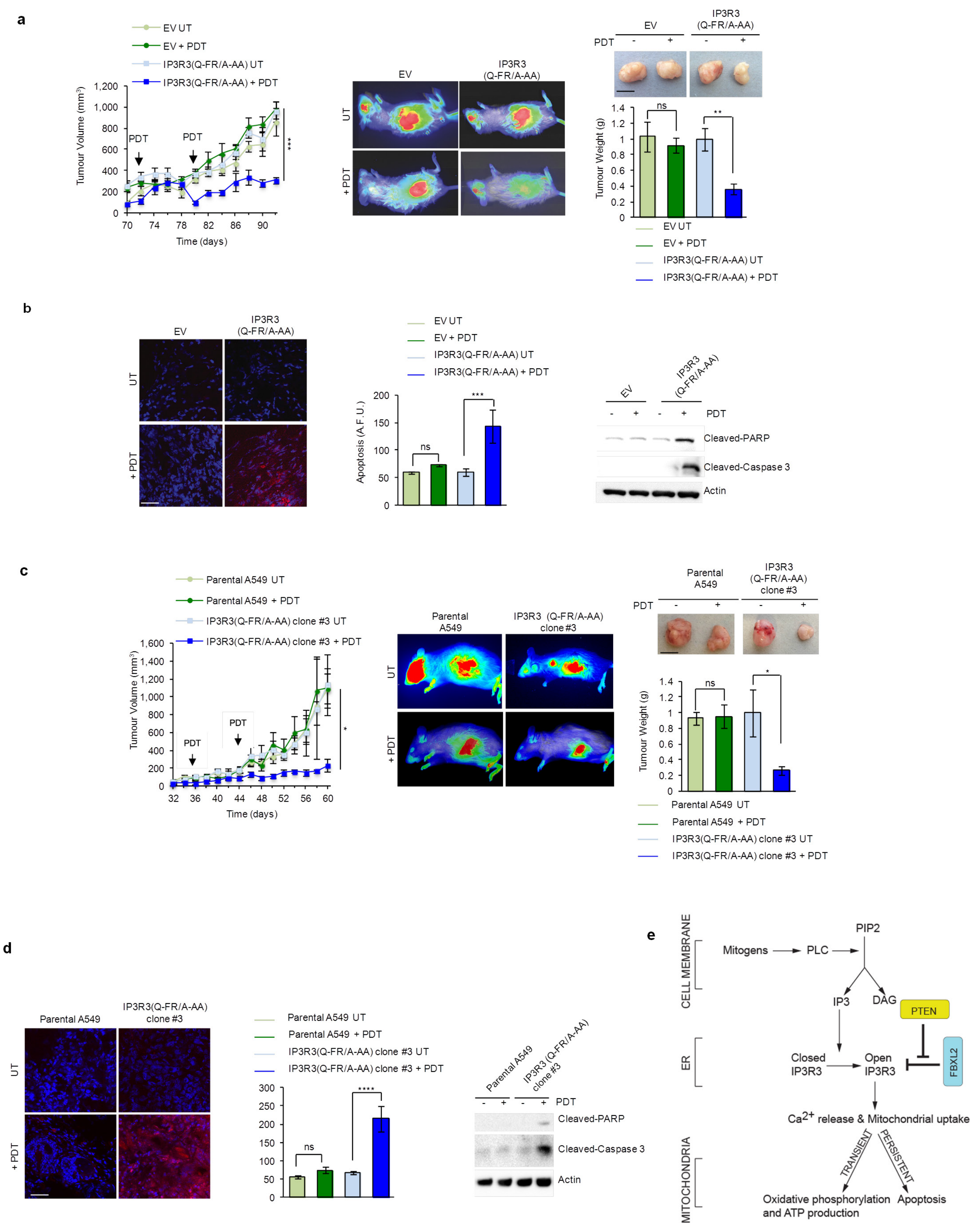

Extended Data Figure 10 | See next page for caption.

© 2017 Macmillan Publishers Limited, part of Springer Nature. All rights reserved.

Extended Data Figure 10 | A non-degradable IP3R3 mutant and GGTi-2418 sensitize tumours to photodynamic therapy. a, Tumour growth of PC3 cell xenografts analysed as in (Fig. 4a). Error bars indicate s.e.m. **b**, Apoptosis of PC3 cell xenografts analysed as in (Fig. 4b). Error bars indicate s.e.m. **c**, A549 parental cells and IP3R3(Q-FR/A-AA) knock-in clone no. 3 were processed as in (Fig. 4a). **d**, A549 parental cells and IP3R3(Q-FR/A-AA) knock-in clone no. 3 were processed as in (Fig. 4b). **e**, A model of the FBXL2- and PTEN-dependent regulation of IP3R3 function in energy production and cell death. In response to IP3 production, IP3R3 releases calcium from the endoplasmic reticulum (ER) to mitochondria, stimulating oxidative phosphorylation and ATP production. To avoid persistent calcium flux and consequent cell death, IP3R3 is degraded via FBXL2. PTEN competes with FBXL2 for IP3R3 binding, thus increasing the stability of IP3R3 and promoting apoptosis. The fact that PTEN(C124S), a catalytically dead mutant, binds IP3R3, competes with FBXL2 for IP3R3 binding, and stabilizes IP3R3 in a manner that is identical to wild-type PTEN, strongly indicates that neither the lipid phosphatase activity nor the protein phosphatase activity of PTEN are required to positively affect IP3R3 stability. PTEN(G129E), a mutant displaying a greatly reduced lipid phosphatase activity, but retaining protein phosphatase activity, also binds IP3R3 and competes with FBXL2 in a manner that is indistinguishable from wild-type PTEN. However, PTEN(C124S) induces an effect on Ca^{2+} mobilization that is significant, but not as high as that evoked by wild-type PTEN and PTEN(G129E). This suggests that, in addition to its phosphatase-independent ability to stabilize IP3R3,the protein phosphatase activity of PTEN may contribute to Ca^{2+} flux, as suggested by Bononi *et al.*[40]. Our findings reveal the molecular basis (that is, the competition with FBXL2 for IP3R3 binding) by which PTEN(C124S) is able to promote both a mitochondrial Ca^{2+} response and apoptosis. Importantly, according to this model, FBXL2 is a pro-survival factor, which complements its known role in the efficient activation of the PI3K cascade[9]. Finally, our results show that both FBXL2 and PTEN do not affect the levels and stability of IP3R1 and IP3R2. We note that one peptide corresponding to IP3R2 was identified in the original purification of the FBXL2 complex (ftp://odr.stowers.org/LIBPB-484). Moreover, the FBXL2 complex purified by the Harper group contained one peptide corresponding to IP3R1 (ref. 41). FBXL2 binds both p85α and p85β; but it targets only p85β for degradation[9]. We speculate that the binding to p85α is indirect and occurs because of the presence in the cell of p85α-p85β heterodimers. Since IP3R1, IP3R2, and IP3R3 also form heteromers[42–44], it is possible that FBXL2 indirectly binds one or both IP3R3 paralogues, but only targets IP3R3 for degradation. Unless otherwise noted, experiments were performed at least three times. For gel source data, see Supplementary Fig. 1.

© 2017 Macmillan Publishers Limited, part of Springer Nature. All rights reserved.
