# Supplementary figures and images for: PTEN counteracts FBXL2 to promote IP3R3– and Ca2+–mediated apoptosis limiting tumour growth
Source: Nature. Author manuscript; Available in PMC 2017 Oct 4. (PMC5627969; doi:10.1038/nature22965)

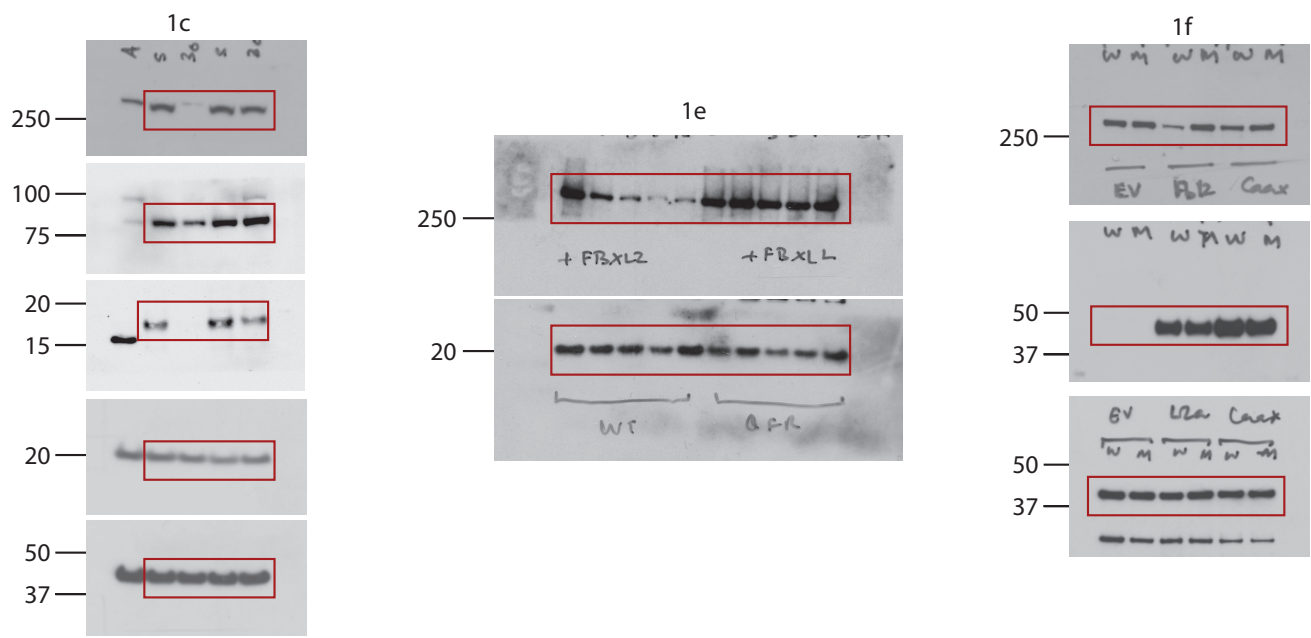

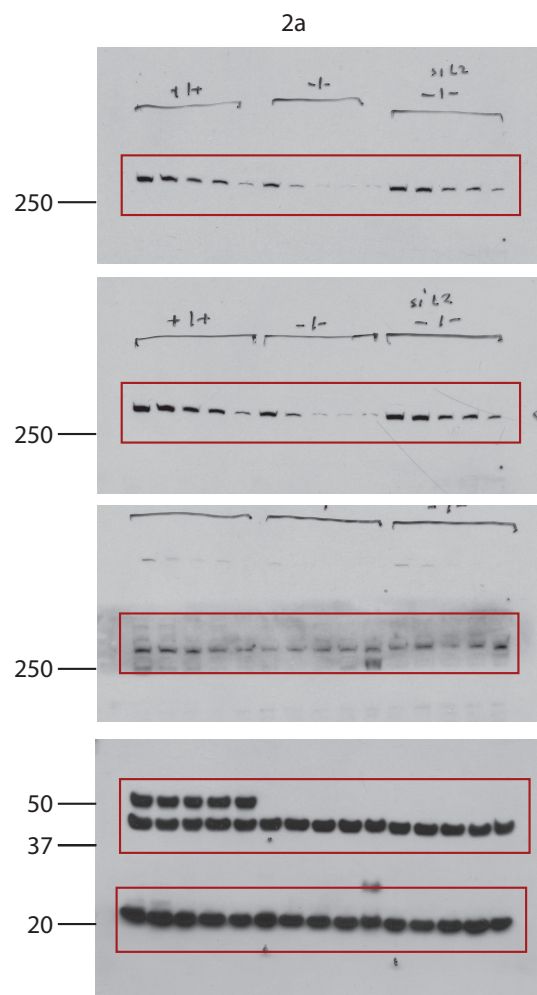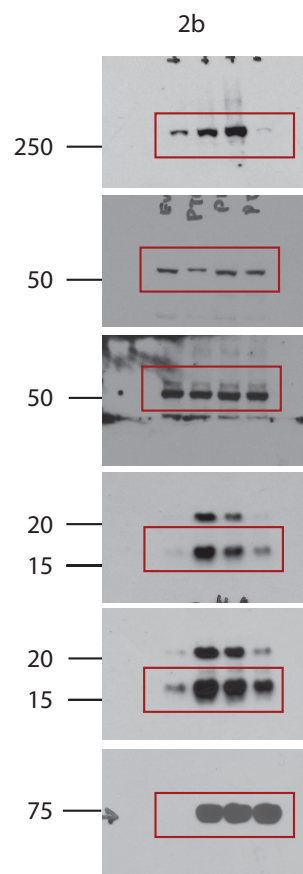

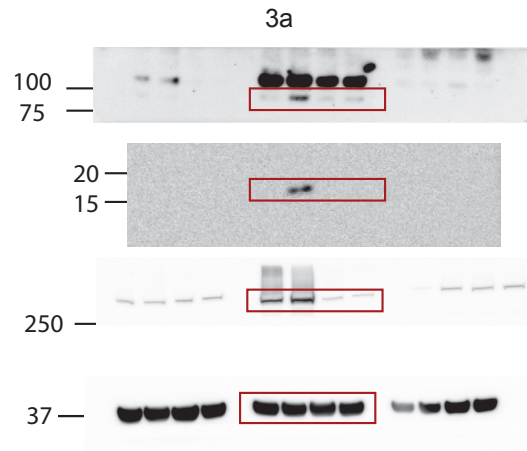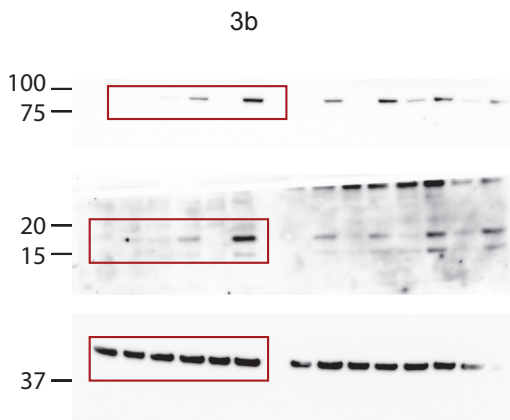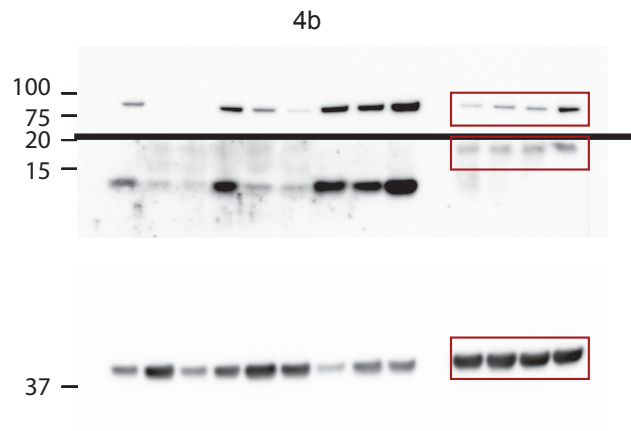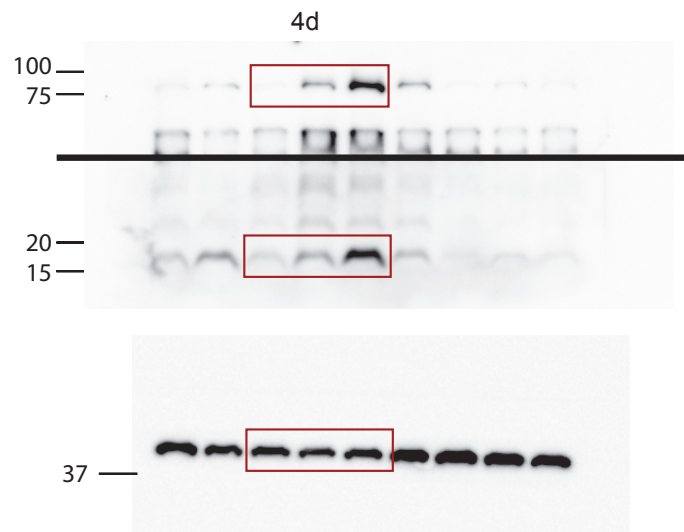

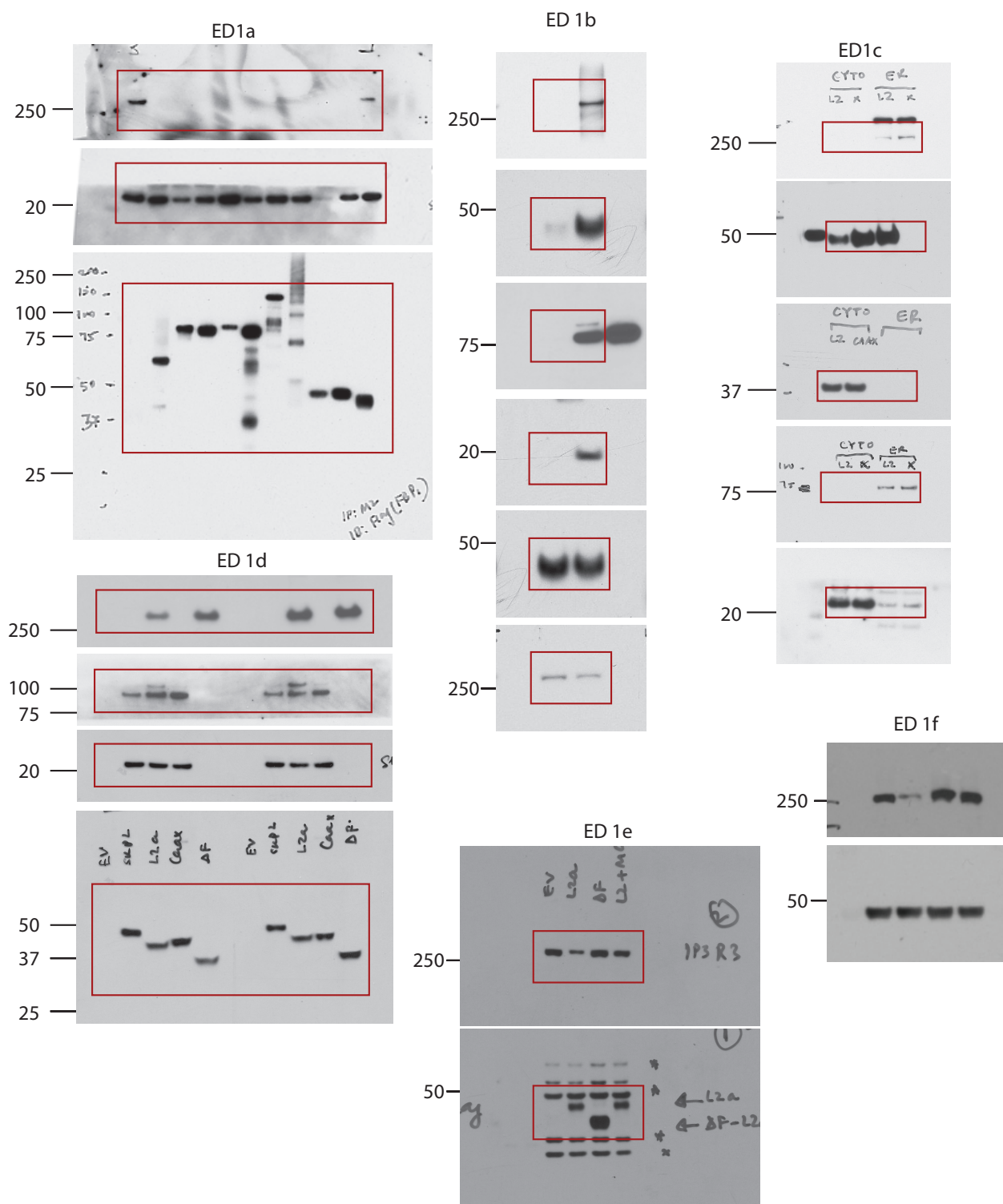

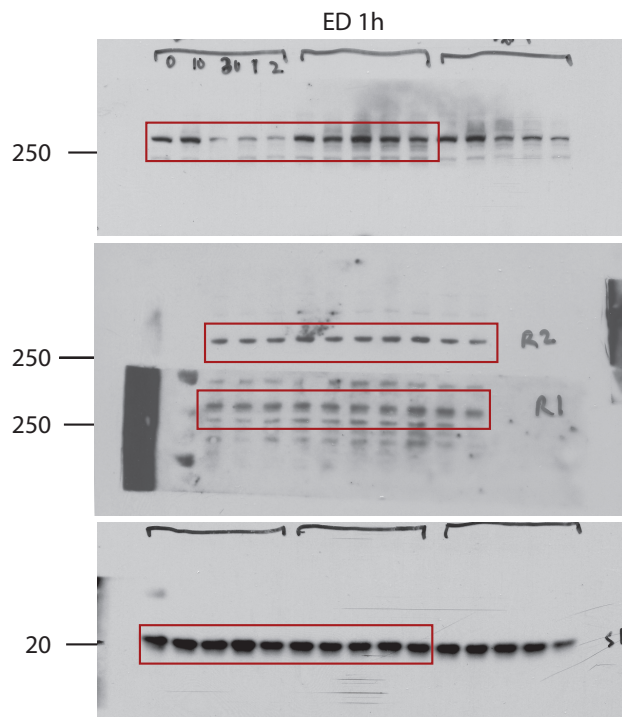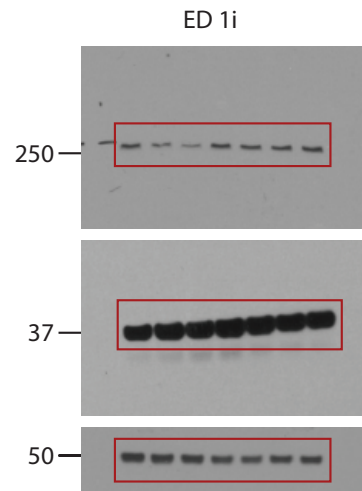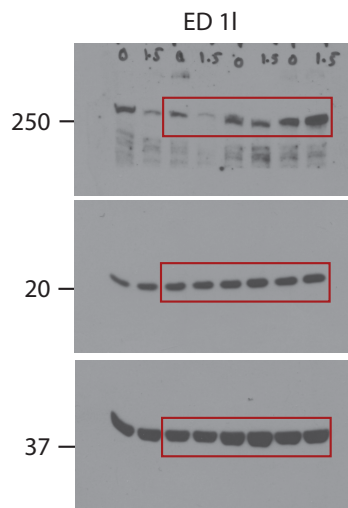

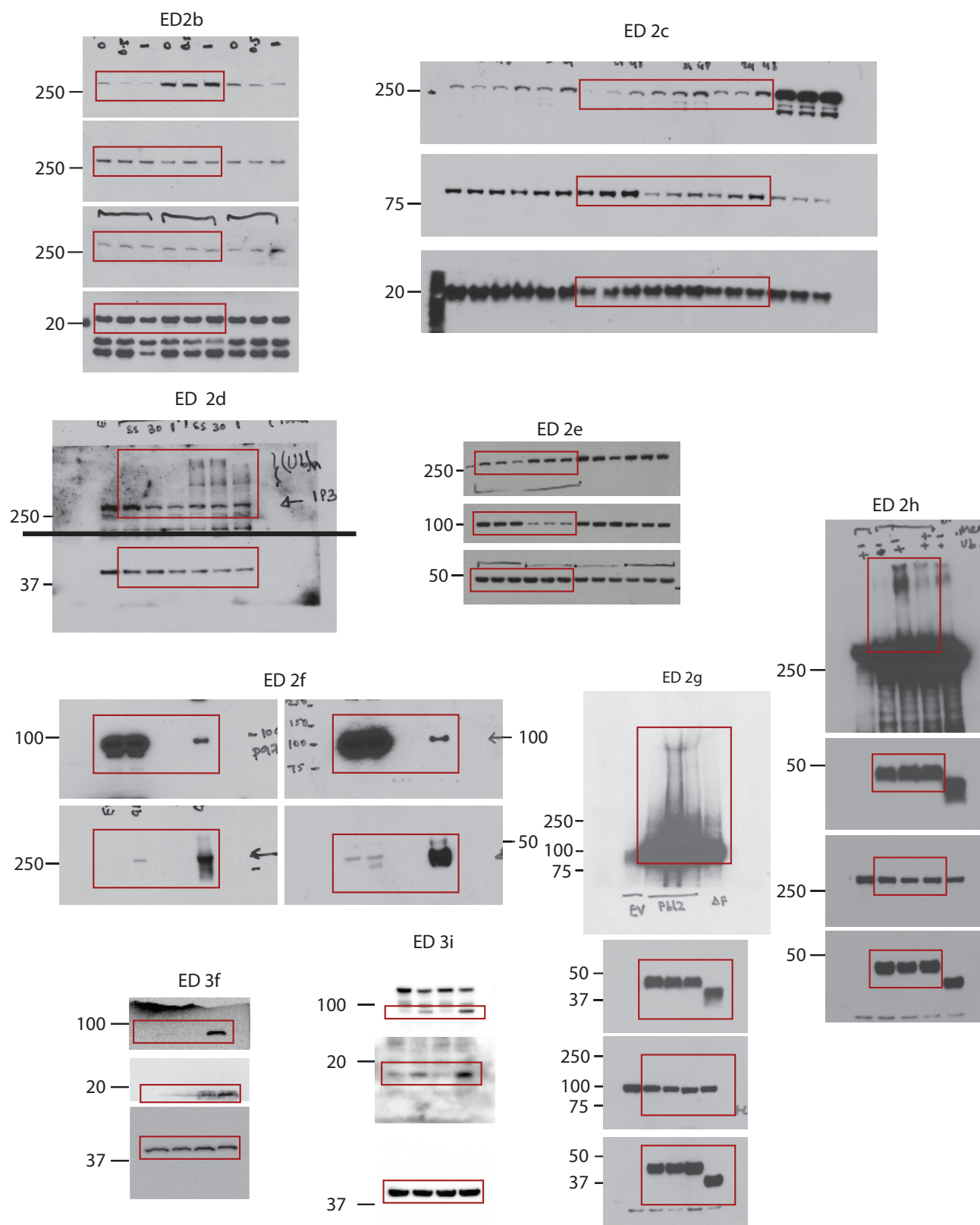

ED 4b

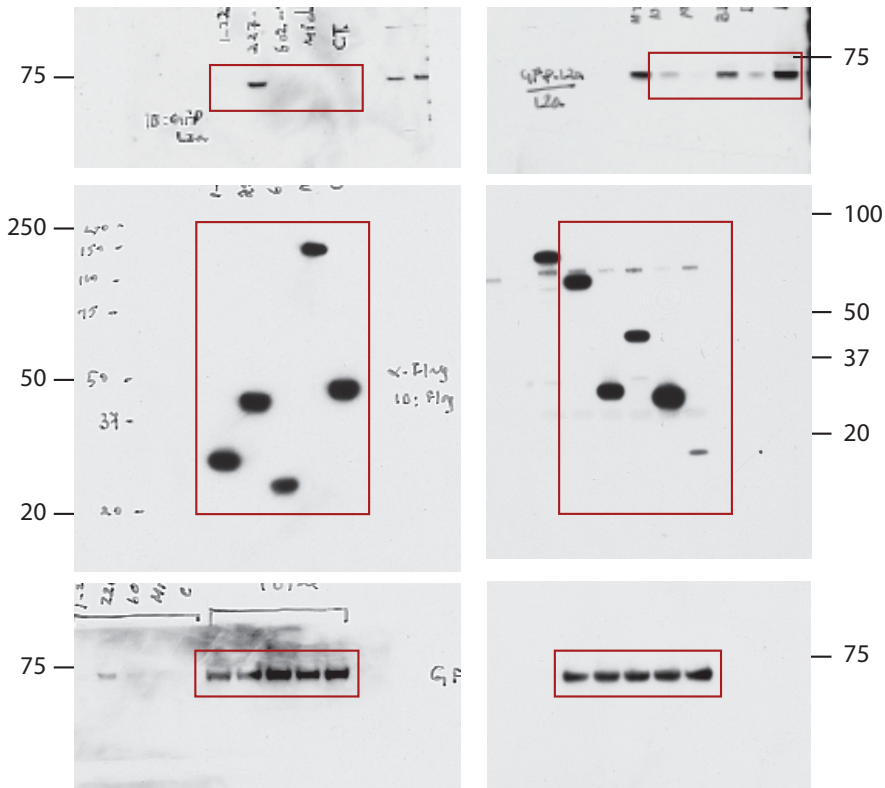

ED 4c

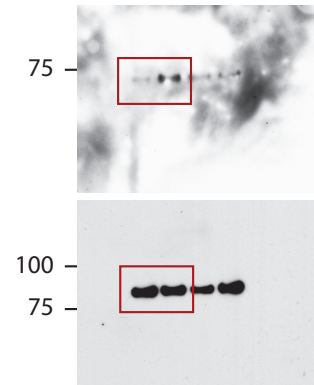

ED 4d

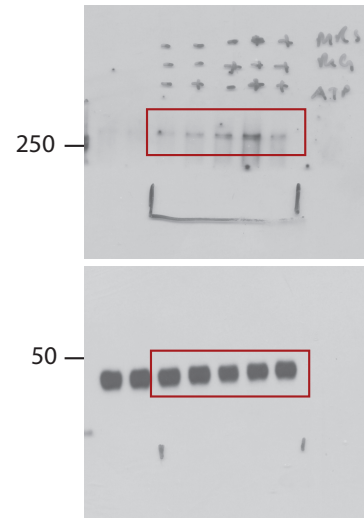

ED 4e

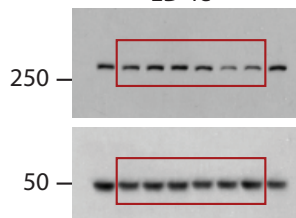

ED 4f

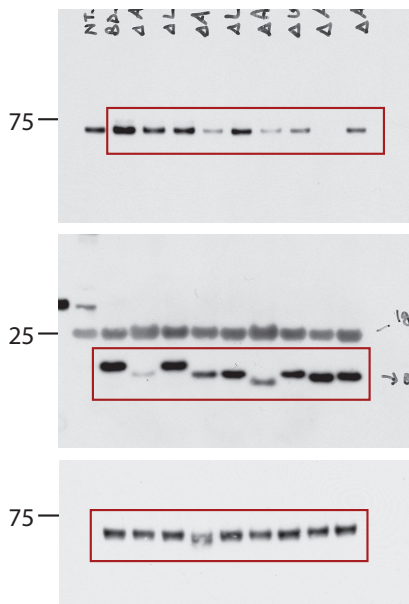

ED 4g

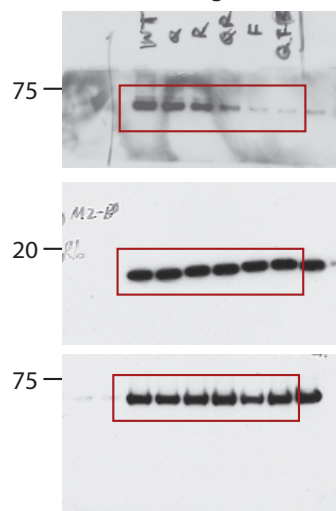

ED 4j

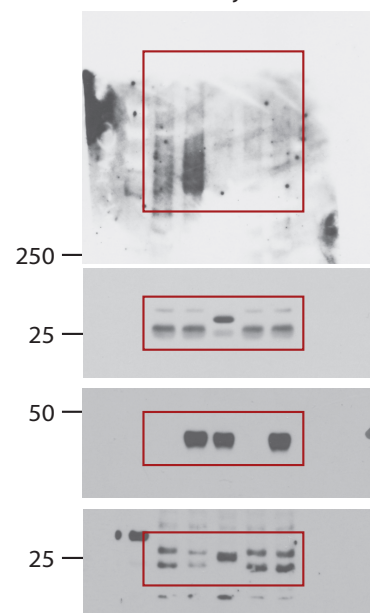

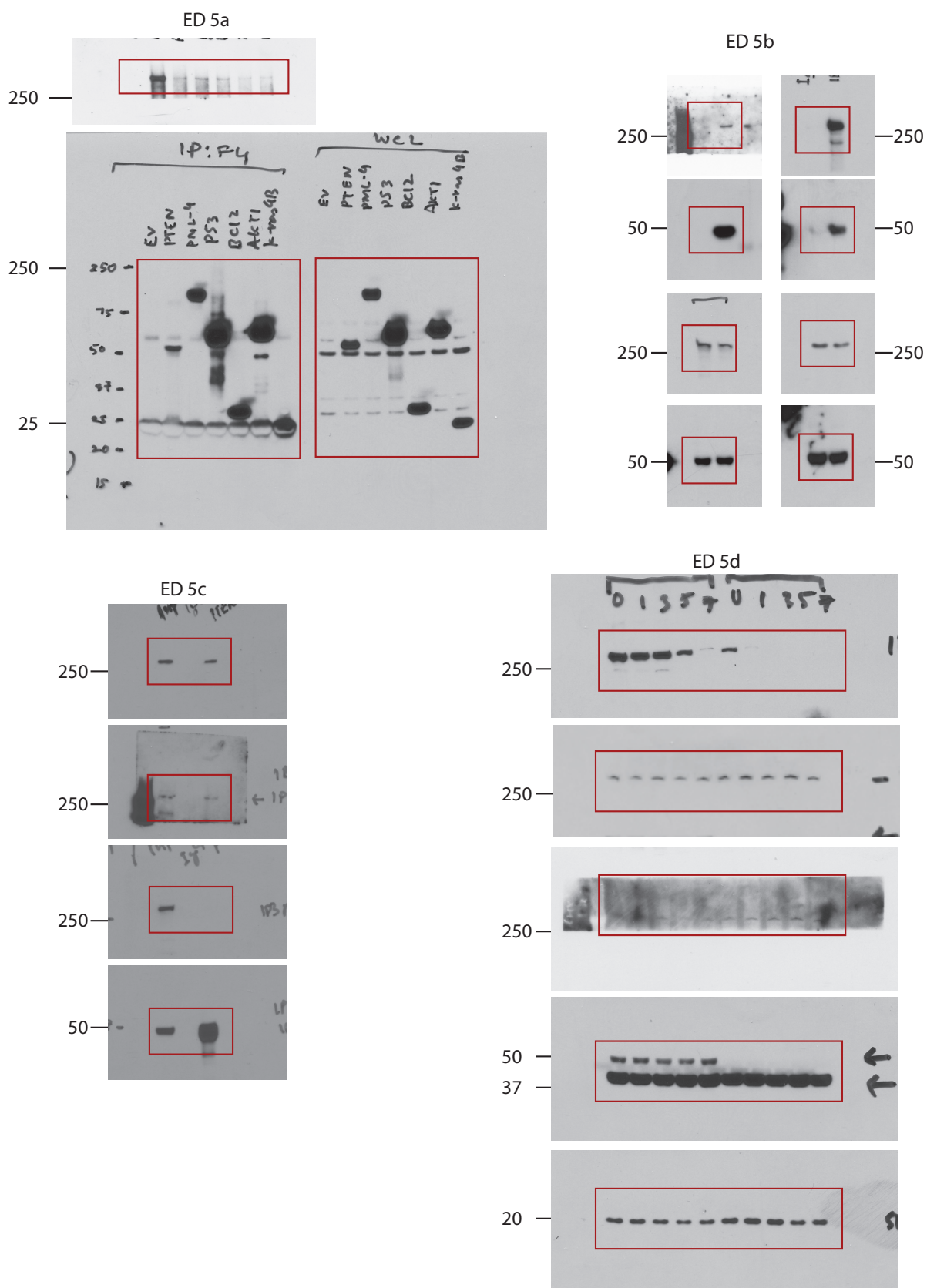

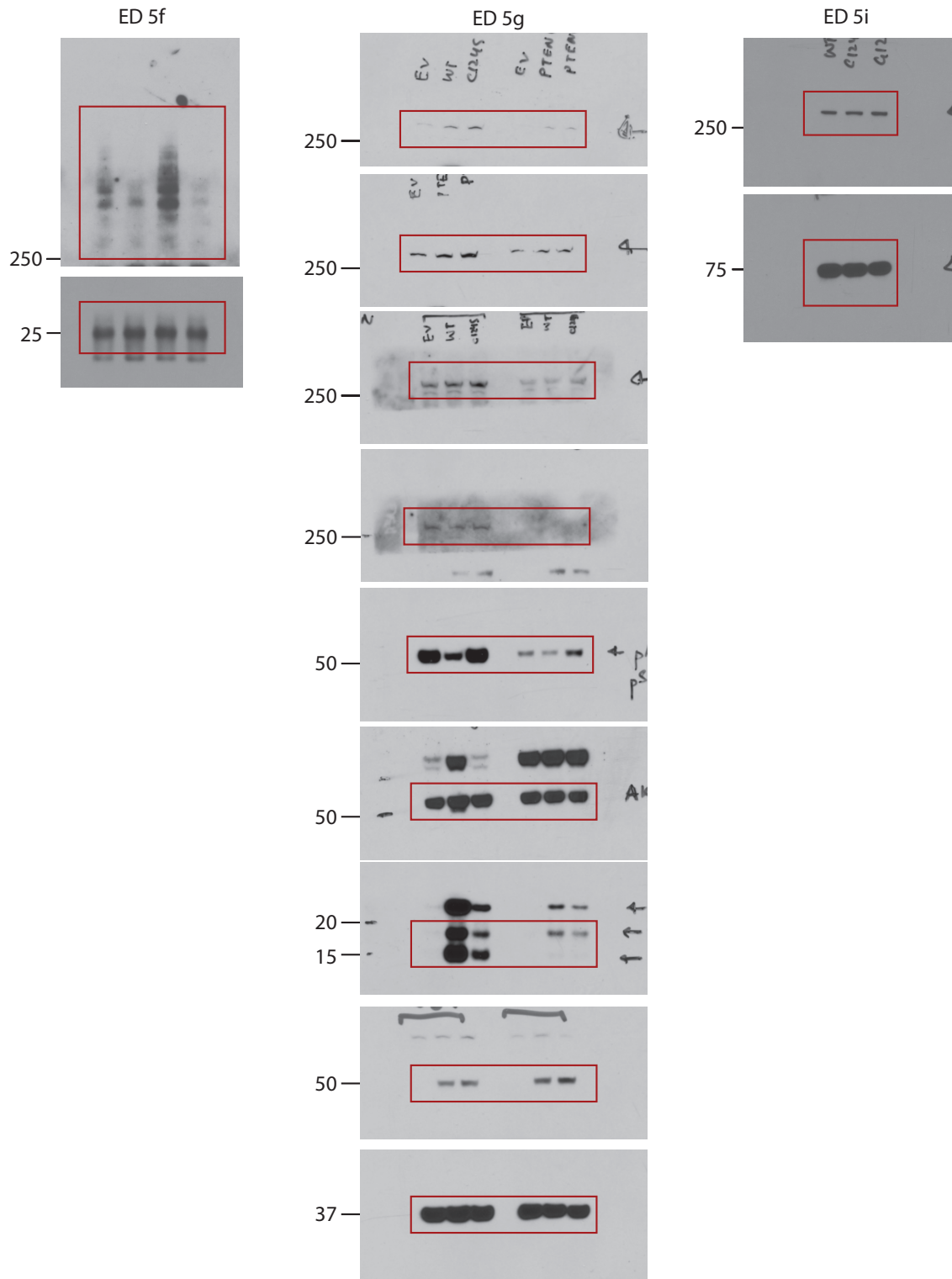

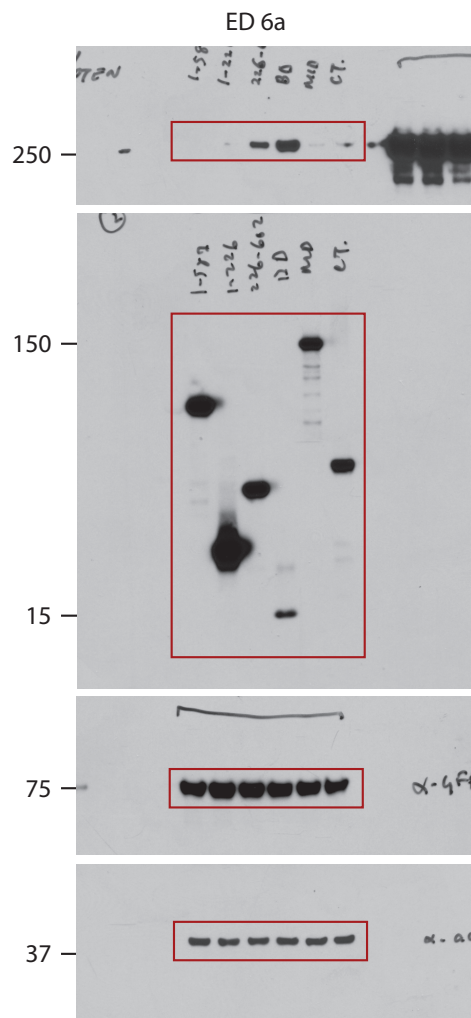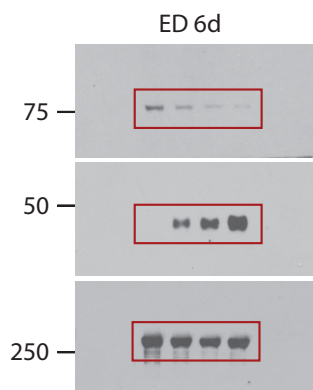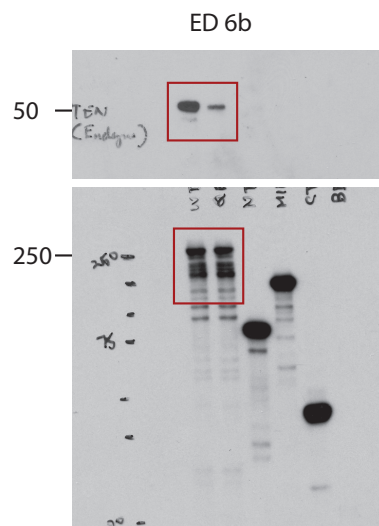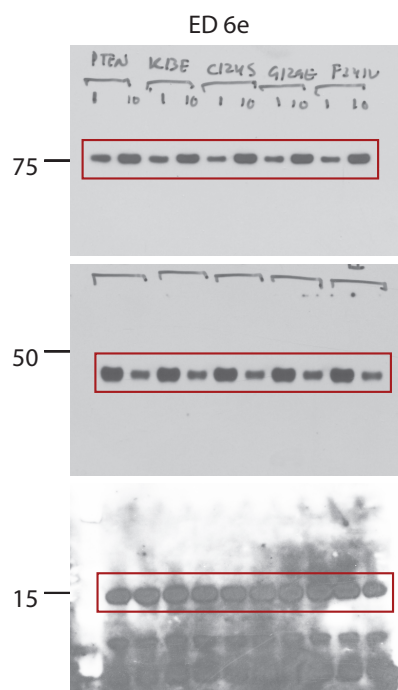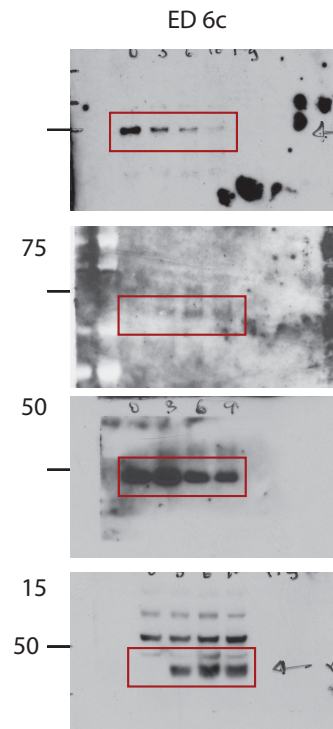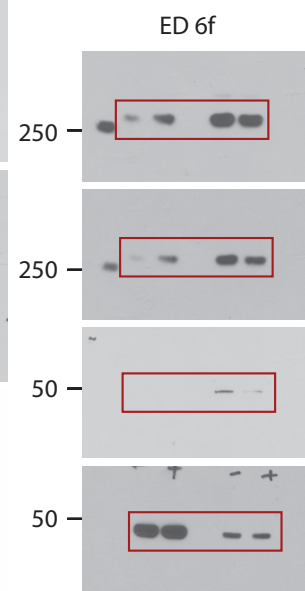

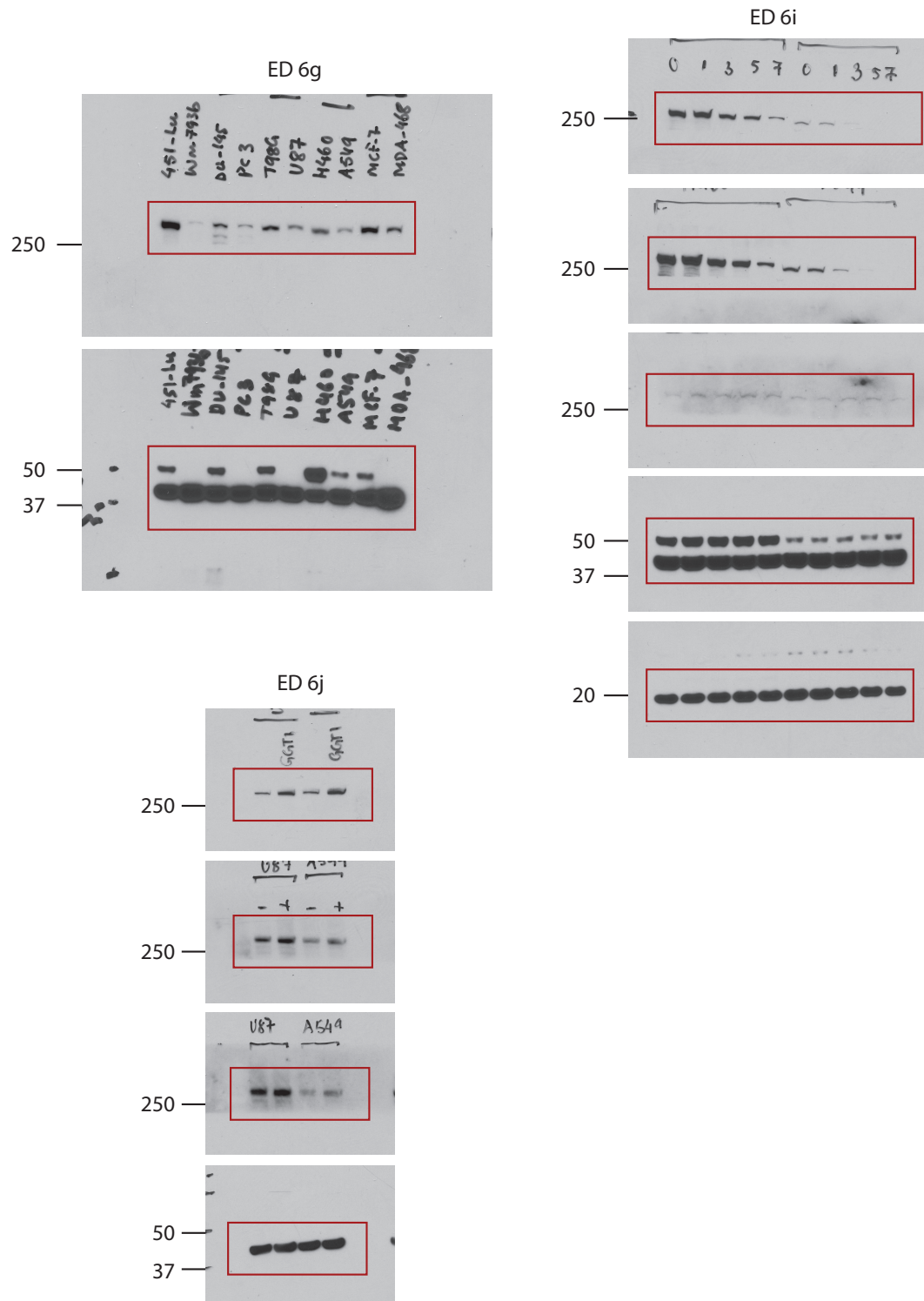

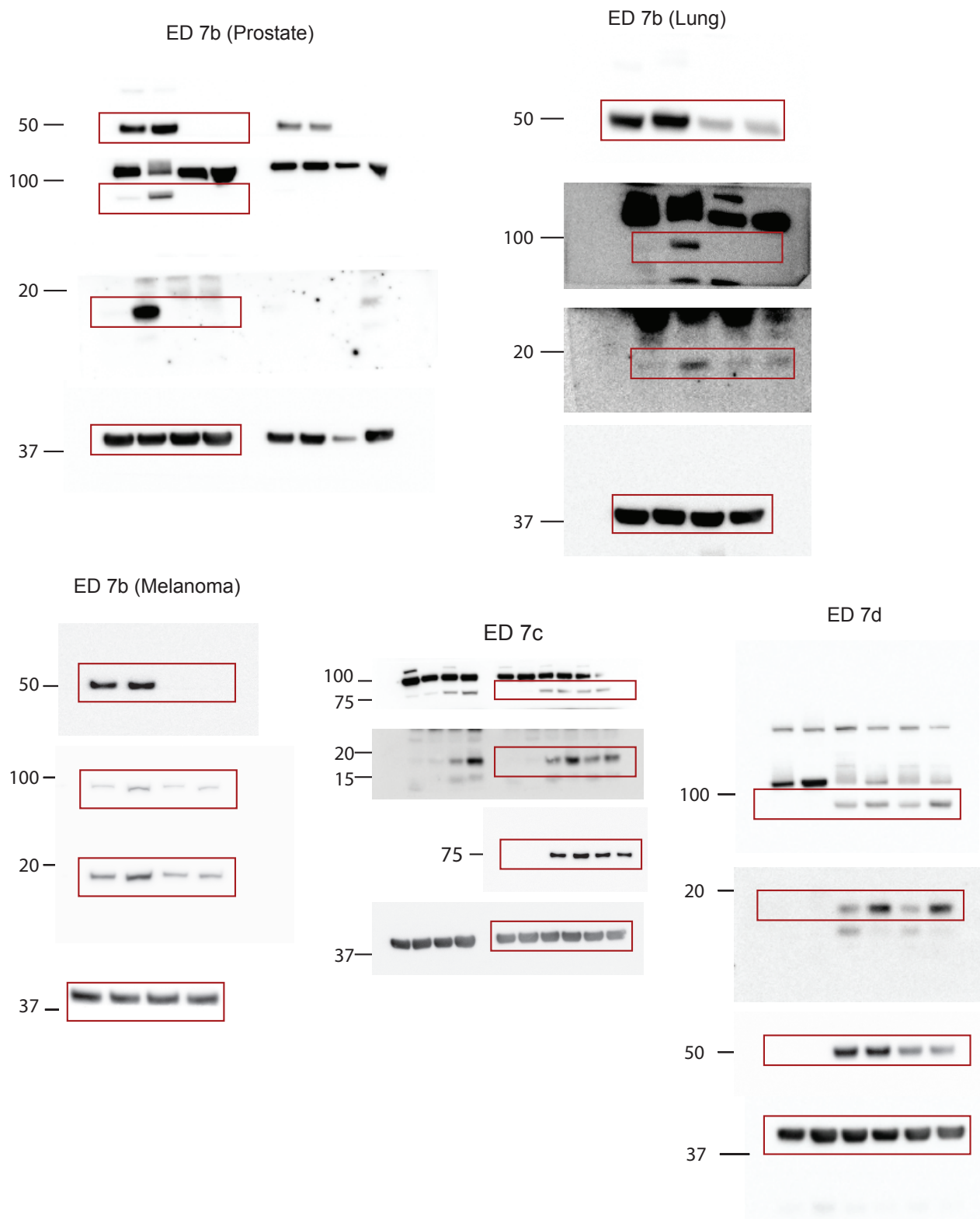

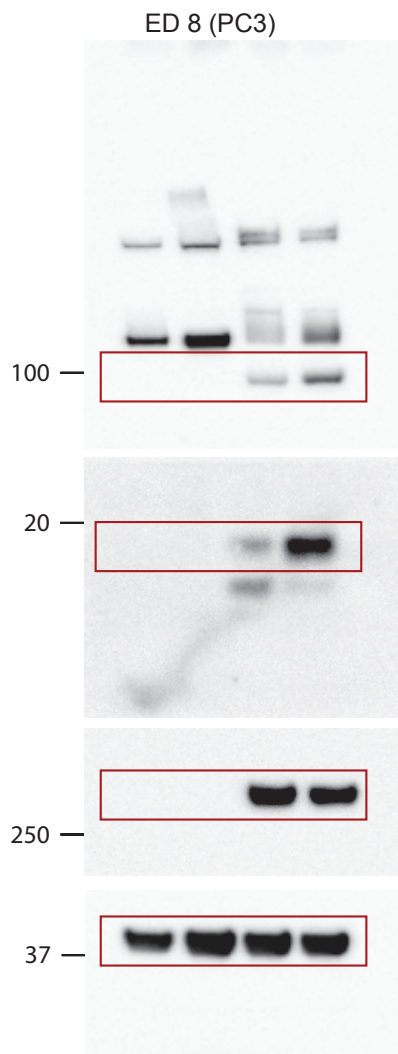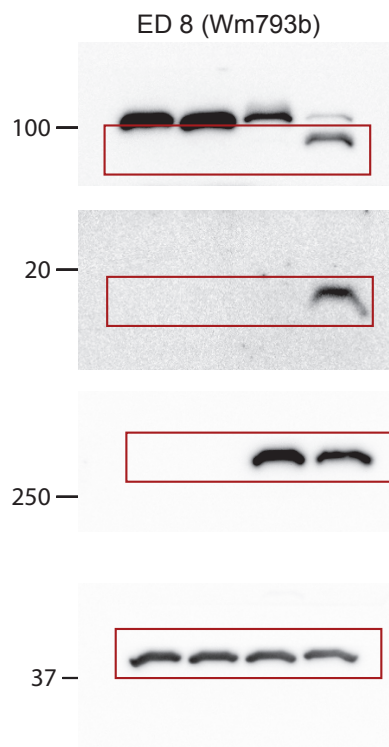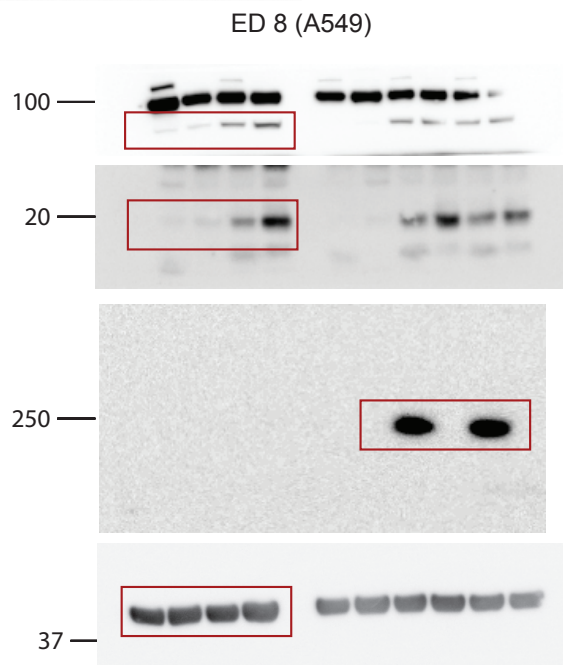

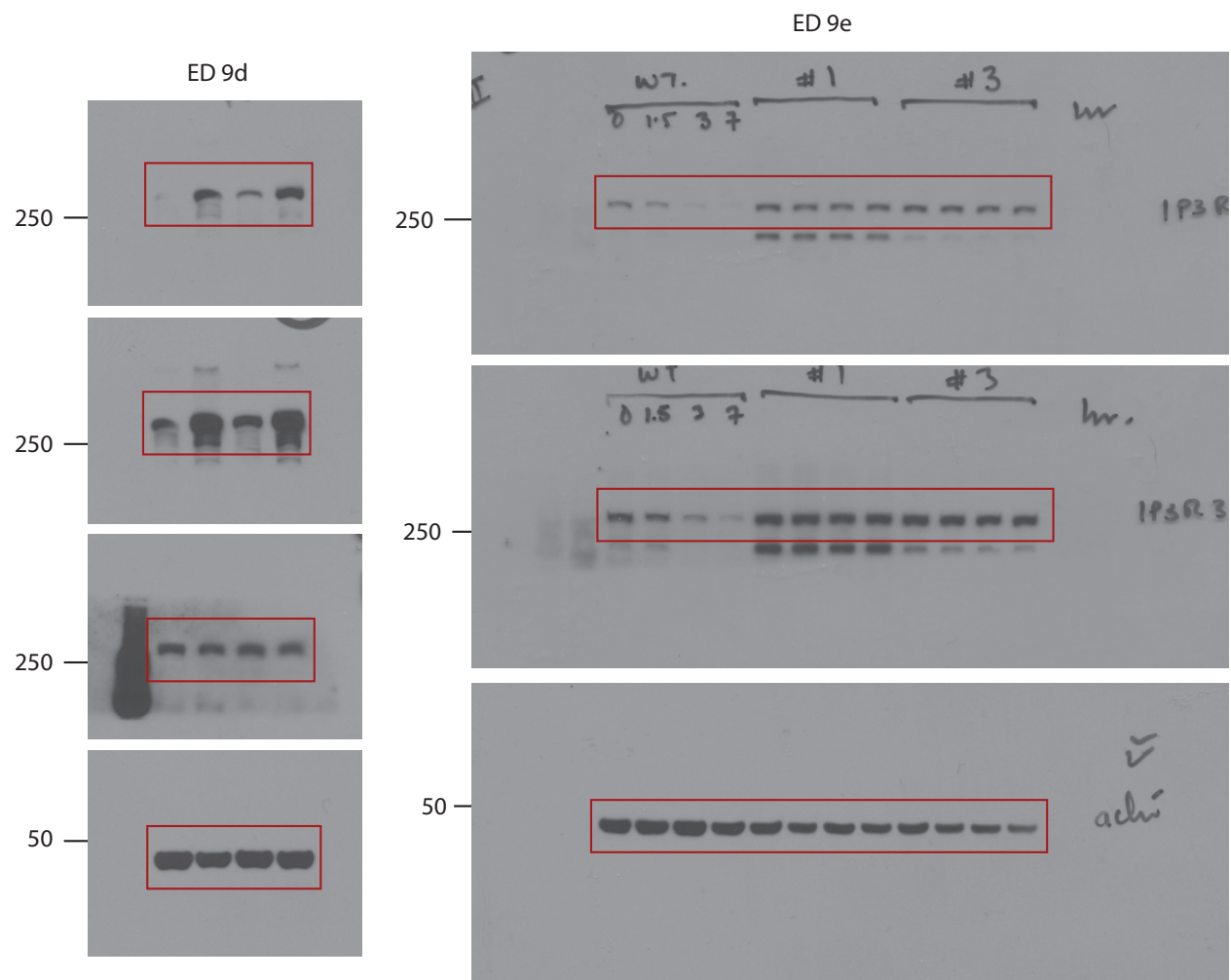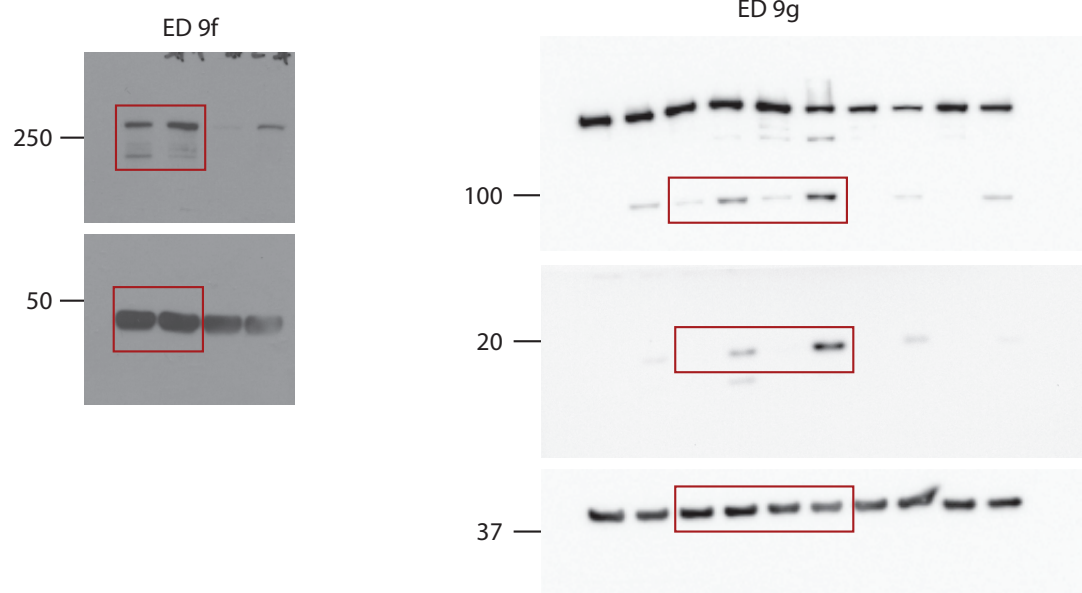

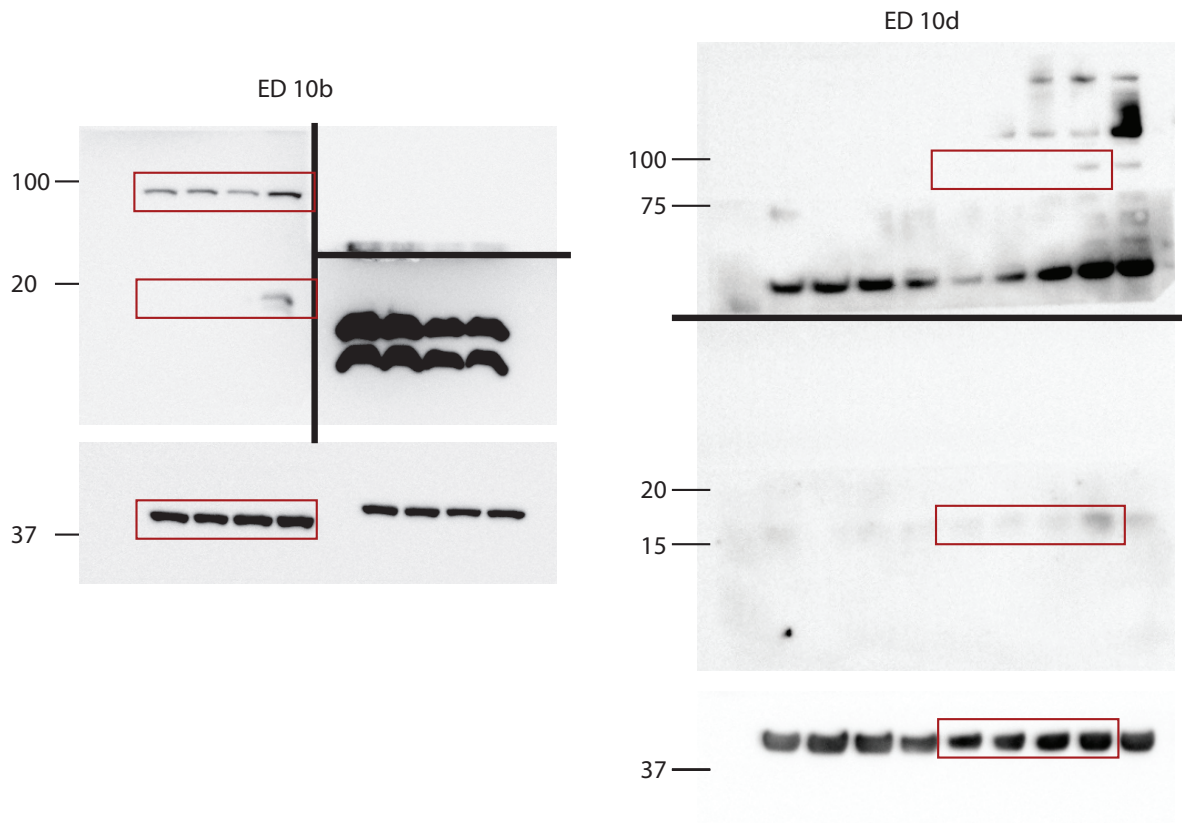

Supplement: Supplemental 2 [file NIHMS906616-supplement-Supplemental_2.pdf]
